# Compensatory Fatty Acid Metabolism and Hepatic Gene Expression in African Catfish (*Clarias gariepinus*) Fed Marine‐Ingredient‐Free Circular Diets Low in EPA and DHA

**DOI:** 10.1155/anu/4162985

**Published:** 2026-06-11

**Authors:** Christopher Shaw, Klaus Knopf, Sven Wuertz, Koushik Roy, Radek Gebauer, Tobias Goldhammer, Viola Schöning, Wibke Kleiner, Marvin Sens, Christian Ulrichs, Werner Kloas

**Affiliations:** ^1^ Leibniz Institute of Freshwater Ecology and Inland Fisheries, Berlin, Germany, igb-berlin.de; ^2^ Albrecht Daniel Thaer Institute of Agricultural and Horticultural Sciences, Humboldt University Berlin, Berlin, Germany, hu-berlin.de; ^3^ Faculty of Fisheries and Protection of Waters, South Bohemian Research Center of Aquaculture and Biodiversity of Hydrocenoses, Institute of Aquaculture and Protection of Waters, University of South Bohemia in Ceske Budejovice, České Budějovice, 370 05, Czech Republic, jcu.cz; ^4^ Faculty of Life Sciences, Division Urban Plant Ecophysiology, Humboldt-University of Berlin, Lentzeallee 55/57, Berlin, 14195, Germany; ^5^ Institute of Biology, Humboldt University Berlin, Berlin, Germany, hu-berlin.de

**Keywords:** circular protein sources, *Clarias gariepinus*, DHA, EPA, fatty acid metabolism, gene expression, marine ingredient replacement

## Abstract

Raw materials rich in EPA (C20:5n‐3) and DHA (C22:6n‐3) such as marine fish oils and meals are becoming increasingly scarce and valuable throughout the aquafeed industry, while human dietary intake of EPA and DHA is deficient in many parts of the world. Therefore, EPA and DHA sources have to be used judiciously and strategically in diets for especially freshwater fish, which, compared to their marine counterparts, tend to have a superior ability to biosynthesize these functionally important fatty acids (FAs) from their C_18_ precursors. Hence, this study tested the effect of a minimal dietary EPA + DHA level on growth performance, fillet and liver FA profile development, gene expression dynamics (*elovl2*, *elovl5*, and *fads2*), and nutrient excretion of African catfish (*Clarias gariepinus*) by replacing salmon oil with rapeseed oil in a marine‐ingredient‐free basal diet formulated with low EPA + DHA circular animal protein sources. Even though growth performance attained with a commercial African catfish diet utilizing the same salmon oil was not achieved with the two experimental diets, low EPA + DHA levels in the rapeseed oil experimental diet did not negatively affect growth. Expression of hepatic *elovl2*, *elovl5*, and *fads2* was significantly elevated in the rapeseed oil‐fed fish, while particularly EPA and DHA in the fillet, and EPA in the liver were consistently and significantly lower compared to the salmon oil‐based diets. Concomitant examination of gene expression and FA profile development indicates the notable capacity of African catfish to de novo synthesize EPA and DHA from ALA (C18:3n‐3), which suggests the potential of low EPA + DHA diets for this species. From a human nutritional point of view, however, this biosynthetic capacity was insufficient to compensate for the effect of the low EPA + DHA rapeseed oil diet on fillet FA profile quality, with EPA + DHA content and n‐6:n‐3 FA ratio being significantly negatively affected. Irrespective of oil source, the phosphorus‐rich experimental diets resulted in a significantly improved ratio of excreted dissolved nitrogen to phosphorus compared to the industrial control from a plant nutrition perspective, confirming prior results on the ability to improve diets for use in aquaponics.

## 1. Introduction

Today, it is widely accepted that omega‐3 long‐chain polyunsaturated fatty acids (n‐3 LC‐PUFAs), especially eicosapentaenoic acid (EPA, C20:5n‐3) and docosahexaenoic acid (DHA, C22:6n‐3), provide important human health benefits such as lowering the risk of a wide range of cardiovascular diseases, improving infant brain development, and ameliorating obesity [1]. Marine capture fisheries are the primary source of EPA and DHA, with half of the extracted amount being directly consumed by humans and the other half primarily by the aquaculture industry via fish meal and oil [2]. However, considering the ongoing growth of global aquaculture [3] and the already deficient global human dietary intake of EPA and DHA [4, 5], sources rich in these fatty acids (FAs) are becoming increasingly scarce and expensive [6, 7]. In the face of this scarcity, the aquaculture industry as a consumer as well as a producer of EPA and DHA faces the challenge of first needing to cover the nutritional requirements of farmed species and secondly providing a healthy product high in these n‐3 LC‐PUFAs to consumers. A case in point is the steadily declining EPA and DHA concentrations in Atlantic salmon fillets caused by the continuous replacement of fish meal and oil predominantly with plant proteins and oils [8]. This challenge needs to be addressed by (a) fostering the recycling of EPA and DHA from fisheries and aquaculture by‐products, particularly of marine origin [9, 10], (b) expanding the availability of and tapping into novel EPA and DHA sources (e.g., microalgae, mesopelagic marine fish, marine zooplankton, and transgenic oilseeds) [11], as well as (c) judiciously and strategically utilizing the limited available supply. On the one hand, the last approach implies limiting the dietary inclusion of EPA and DHA to species‐ and life stage‐specific requirements for optimal growth and health. This especially means minimizing the dietary inclusion of EPA and DHA for freshwater fish species, which, compared to marine species, require only minimal dietary intake due to their superior endogenous ability to transform the C_18_ PUFAs α‐linolenic acid (ALA, C18:3n‐3) and linoleic acid (LA, C18:2n‐6) into their LC‐PUFA counterparts EPA, DHA, and arachidonic acid (ARA, C20:4n‐6) through desaturation and elongation [9, 12]. On the other hand, considering the need for health‐promoting final products, this also implies the option to feed fortified diets higher in n‐3 LC‐PUFAs even to freshwater species for a limited period during the final phase of grow‐out to achieve the desired product quality with minimal EPA and DHA expenditure [13–15].

African catfish (*Clarias gariepinus*), one of the major fish species cultured in Sub‐Saharan Africa [16], has been shown to possess the biochemical machinery to desaturate and elongate the C_18_ precursors ALA and LA characteristic for plant oils into physiologically active EPA, DHA, and ARA [17], i.e., the *fads*2, *elovl*2, and *elovl*5 genes encoding the necessary enzymes, respectively [18]. Several studies have already illustrated that either partly or entirely replacing fish oils with various vegetable oils does not result in a negative growth response of African catfish and, in some cases, even improved growth performance, indicating the potential efficacy of its n‐3 LC‐PUFA biosynthetic pathway. However, most of these feeding trials either utilized diets that still provided notable levels of n‐3 LC‐PUFAs due to considerable inclusion of marine fish meal [19–23], which potentially prevented elucidating the actual efficacy of this biosynthetic pathway and effect of an actually limited dietary n‐3 LC‐PUFA level on growth performance and ultimately flesh quality, or, from a practical aquafeed perspective, used unrealistic purified casein‐gelatin‐dextrin‐based experimental diets [24, 25]. Apart from the fundamental work on the existence of the n‐3 LC‐PUFA biosynthetic pathway in *C. gariepinus* [18, 26]; only a few studies have attempted to elucidate the efficacy of this pathway within practical feeding trials [22, 27]. However, these studies faced serious confounding effects in the form of substantial marine fish meal inclusion and concomitantly high dietary EPA and DHA levels [22] as well as considerable growth depression in the plant protein‐based and low EPA and DHA diets potentially caused by essential amino acid imbalances, reduced digestibility and palatability, or the presence of antinutritional factors [27]. Furthermore, the above studies focused solely on endpoint analyses of gene expression, which can be insufficient when trying to illuminate a process that is inherently dynamic in nature [28, 29].

Taking the above assessments regarding n‐3 LC‐PUFAs into account as well as considering the aquafeed industry’s general need to transition to sustainable, increasingly circular, and economically viable alternatives to marine fish meal and oil from capture fisheries [30–32], it is indispensable to investigate the adequacy of novel marine‐ingredient‐free feed formulations from the perspective of FA metabolism and EPA and DHA savings potential. Therefore, this study set out to elucidate the growth performance, fillet and liver FA profile development as well as hepatic *fads*2, *elovl*2, and *elovl*5 mRNA expression dynamics over the course of a RAS (recirculating aquaculture system) feeding trial with African catfish in which a feed formulation entirely based on low EPA and DHA circular animal protein sources—poultry by‐product meal (PM), freshwater fish by‐product meal (FFM), black soldier fly larvae meal (BSFM), and poultry blood meal (PBM)—and rapeseed oil was compared with the same formulation based on EPA‐ and DHA‐rich salmon oil as well as an industrial standard diet based on the same salmon oil (Alltech Coppens Special Pro EF 4.5 mm; AC). Aside from circularity reasons, this basic feed formulation approach was not only chosen to minimize the EPA and DHA content in the rapeseed oil diet, i.e., to entirely spare EPA and DHA raw materials, but also to achieve an improved ratio of dissolved nitrogen to phosphorus (N:P ratio) in the RAS water along the lines of an aquaponic feed that aims to reduce phosphorus fertilizer dependency in aquaponic plant production [33, 34].

Since the dynamics of *fads*2, *elovl*2, and *elovl*5 expression in conjunction with muscle and liver FA dynamics have not been investigated in African catfish over the course of a trial employing practical low EPA and DHA diets that support adequate growth performance, this study set out to narrow this gap. Such an approach not only aids in better understanding the basic biosynthetic capabilities of African catfish from a fundamental perspective, but results can also help to practically address the issue of global EPA and DHA resource scarcity by highlighting possible pathways towards harnessing such biosynthetic capabilities for more efficient use of these valuable resources. Hence, the main research questions of this study include how the above low EPA and DHA rapeseed oil diet in comparison to EPA‐ and DHA‐rich diets affects (a) the expression of hepatic *fads*2, *elovl*2, and *elovl*5 genes over time, (b) the liver and muscle FA profile over time, and (c) the growth performance, as well as (d) if such circular feed formulations, independent of oil source, can support similar growth performance of African catfish compared to an industrial standard diet while improving the N:P ratio in the RAS process water in favor of hydroponically cultivated plant requirements.

## 2. Materials and Methods

### 2.1. Experimental Diets Formulation

First, the proximate composition as well as the EPA, DHA, LA, and ALA content of the AC diet were analytically determined. On the basis of the circular animal protein sources PM, FFM, BSFM, and PBM, two experimental diets (4.5 mm) were formulated in an isonitrogenous (crude protein [CP]: 42.2%–42.3% as fed) and isolipidic (crude fat [CF]: 11.8%–12.4%) manner compared to the AC diet (CP: 43.5%, CF: 13.6%) (Table 1), both only differing in terms of their supplementary oil source (rapeseed oil vs. salmon oil). Formulation objectives were to minimize the EPA and DHA content in the rapeseed oil experimental diet (LO) while achieving a similar LA and ALA content compared to the AC control diet, to reach a similar EPA and DHA content in the salmon oil‐based experimental diet (HI) compared to the AC control diet, and to maximize the phosphorus content of the two experimental diets with the goal of increasing the fish’s release of dissolved P versus N. The vitamin and mineral premixes, as well as the salmon oil, were provided by Alltech Coppens and identical to what was used in the AC diet. The premixes were included in the experimental diets at the same level as that in the AC diet. Diets were extruded at 140°C and vacuum oil‐coated at Pontus Research Ltd., Aberdare (UK), with an oil inclusion level of 4.6% and the objective of thereby matching the CF content of the AC diet (13.6%) and stored in a dark cooling chamber at 3°C until use. Formulations, proximate compositions, amino acid and elemental compositions, as well as FA composition of the diets are compiled in Tables 1, 2, and 3.

**Table 1:** Diet formulations and proximate composition.

Composition	Experimental diets		
	AC^a^	HI	LO
Ingredient composition (%)			
Poultry by‐product meal		24.8	24.8
Freshwater fish by‐product meal		16.0	16.0
Black soldier fly larvae meal		10.0	10.0
Poultry blood meal		10.0	10.0
Wheat		34.0	34.0
Salmon oil^b^	2.4	4.6	—
Rapeseed oil		—	4.6
Vitamin and mineral premix A^c^	0.3	0.3	0.3
Vitamin and mineral premix B^c^	0.3	0.3	0.3
Proximate composition (%–as fed)			
Dry matter (DM)	90.8	89.7	90.5
Moisture	9.2	10.3	9.5
Crude protein (CP) (N × 6.25)	43.5	42.2	42.3
Crude fat (CF)	13.6	12.4	11.8
Crude fiber	1.5	2.2	2.7
Ash	7.0	9.4	9.6
Nitrogen‐free extract^d^	25.2	23.5	24.1
Gross energy (MJ/kg)^e^	20.0	18.9	18.8
P/E ratio (g protein/MJ GE)	21.8	22.3	22.5

^a^Alltech Coppens Special Pro EF 4.5 mm.

^b^Provided by Alltech Coppens.

^c^Provided by Alltech Coppens; included in the experimental diets at the same level as in the AC diet.

^d^NFE = 100%−(% CP + % CF + % CFB + % ash + % moisture).

^e^Calculated using the factors 17.15, 23.64, 39.54 MJ/kg for NFE (carbohydrates), CP and CF, respectively [35].

**Table 2:** Diet amino acid and elemental compositions.

Amino acids and elements	Experimental diets			Requirements
	AC	HI	LO	IAFFD^a^
Essential amino acids (EAAs) (%–as fed)				
Arginine (Arg)	2.49	2.22	2.22	1.79
Histidine (His)	0.75	1.00	1.00	0.79
Isoleucine (Ile)	1.54	1.33	1.32	1.24
Leucine (Leu)	2.84	2.74	2.75	2.39
Lysine (Lys)	2.22	2.31	2.33	2.06
Methionine (Met)	0.72	0.65	0.65	0.8
Phenylalanine (Phe)	1.67	1.50	1.52	1.27
Threonine (Thr)	1.51	1.47	1.48	1.24
Tryptophan (Trp)	0.43	0.48	0.49	0.3
Valine (Val)	1.85	1.77	1.76	1.51
Met + Cys	1.36	1.00	1.01	1.08
Phe + Tyr	2.75	2.52	2.54	2.2
Sum EAAs	16.02	15.47	15.52	—
Nonessential amino acids (NEAAs) (%–as fed)				
Alanine (Ala)	2.27	2.67	2.61	
Cysteine (Cys)	0.64	0.35	0.36	
Glycine (Gly)	3.1	3.34	3.31	
Proline (Pro)	2.88	2.38	2.41	
Serine (Ser)	2.07	1.52	1.54	
Aspartic acid (Asp) + asparagine (Asn)	3.16	3.06	2.98	
Glutamic acid (Glu) + glutamine (Gln)	6.89	5.16	4.97	
Tyrosine (Tyr)	1.08	1.02	1.02	
Sum NEAAs	22.09	19.5	19.2	
Elements (g/kg–as fed)^b^				
Ca	15.22	26.17	25.82	
P	10.91	15.76	15.54	
K	6.60	5.79	5.95	
S	5.27	4.04	4.11	
Na	3.23	2.62	2.64	
Mg	1.31	1.47	1.51	
Fe	<0.30	0.50	0.52	
Al	<0.03	0.17	0.18	
Zn	0.10	0.10	0.10	
Mn	0.04	0.05	0.05	
Cu	0.01	0.01	0.01	

^a^Essential amino acid requirements for African catfish (200–500 g) according to the IAFFD Nutrition Specification Database (https://app.iaffd.com/asns).

^b^Analyzed in triplicate; values represent the means.

**Table 3:** Diet fatty acid composition.

Fatty acids (g/kg–as fed)^a^	Experimental diets		
	AC	HI	LO
C10:0	0.26	0.22	0.25
C12:0	0.55	1.58	1.72
C13:0	0.11	0.10	0.11
C14:0	1.89	2.07	1.25
C14:1n5	0.16	0.17	0.18
C15:0	0.24	0.28	0.23
C16:0	16.17	19.04	22.58
C16:1n‐9c	3.36	4.25	3.46
C17:0	0.30	0.32	0.29
C17:1n7	0.16	0.13	0.18
C18:0	3.92	5.84	5.83
C18:1n‐9t	0.21	0.30	0.26
C18:1n‐9c	37.54	28.91	41.28
C18:2n‐6c (LA)	22.39	19.16	23.52
C20:0	0.64	0.44	0.64
C18:3n‐6 (GLA)	0.76	0.73	0.82
C20:1n‐9	2.88	2.11	1.14
C18:3n‐3 (ALA)	7.22	4.45	7.74
C20:2n‐6	0.42	0.66	0.32
C22:0	0.34	0.25	0.33
C20:3n‐6 (DGLA)	0.32	0.40	0.36
C22:1n‐9	0.06	0.05	0.06
C20:3n‐3 (ETA)	0.25	0.31	0.21
C20:4n‐6 (ARA)	0.89	1.05	0.94
C22:2n‐6	0.95	0.85	0.84
C24:0	0.34	0.28	0.31
C20:5n‐3 (EPA)	2.15	1.60	0.42
C22:6n‐3 (DHA)	2.89	2.65	0.50
∑ saturated FA	24.76	30.42	33.53
∑ monoene FA	44.36	35.93	46.56
∑ n‐6 FA	25.73	22.84	26.80
∑ n‐3 FA	12.50	9.01	8.88
∑ n‐6:∑ n‐3	2.06	2.54	3.02
EPA + DHA	5.03	4.26	0.93
EPA:DHA	0.74	0.61	0.84

^a^Analyzed in triplicate; values represent the means.

### 2.2. Experimental RAS

The experimental setup included nine independent RAS situated within a greenhouse, each consisting of a circular rearing tank (340 L), a settling tank for sedimentation of larger feces particles (130 L) followed by a drum filter (60 µm mesh), a moving‐bed biofilter (545 L) including a fixed‐bed biofilter placed on top of it and supplied by an auxiliary pump, a main circulation pump, as well as a drip applicator allowing the addition of base (NaOH) into the pump sump for pH stabilization. All piping included, each individual RAS featured a total water volume of 1045 L. Aeration of the fish tanks and the biofilters was provided by air blowers, and a water exchange of 12% of the RAS volume was conducted once daily via manual inlet and outlet valves connected to the pump sump (detailed description in Section 2.3).

### 2.3. Experimental Procedure and Sampling Regime

For 6 weeks before the trial, biofilters were matured and synchronized across all RAS by stocking each with Nile tilapia fed a commercial diet and continuously recirculating water within each RAS as well as between all RAS through pumping connections between the biofilters. 1 day prior to the start of the trial, the systems were emptied, cleaned, and refilled with fresh tap water and again run in series until the introduction of the experimental fish in order to equalize starting conditions, at which point all RAS were disconnected and run independently.

Mixed‐sex African catfish acquired from Nutrition and Food—Bioenergie Lüchow, Altkalen, Germany, were reared on a commercial fish meal and oil‐containing diet (Alltech Coppens Special Pro EF 3.0 mm and 4.5 mm) in a different RAS at 26°C. Fish were not fed for 24 h before transfer to the experimental RAS. In total, 486 fish (142 ± 19 g) were randomly allocated to the experimental system with 54 individuals per RAS, each dietary treatment being replicated in three RAS. Throughout the 42‐day trial, biomass was determined in each RAS on days 0, 21, and 42. Feed rations were decreased linearly on a daily basis from 2.9% to 2.3% of tank biomass and adjusted daily according to biomass projections for each tank, assuming a feed conversion ratio (FCR) of 0.85 across all tanks for the first 3 weeks and applying the actual FCRs (calculated on day 21 after biomass determination) of the first 3 weeks for the biomass projection of the last 3 weeks of the trial. Before the start of the trial, 12 fish were sampled for tissue analyses (skinless fillet and liver tissue for FA analysis, liver tissue for gene expression analysis), and on days 14, 28, and 42, an additional 2 fish were sampled per RAS for the same purpose, i.e., 6 fish per dietary treatment per time point. The means of the 2 sampled fish per RAS were subsequently used as the basis for statistical analysis (*n* = 3). Fish were sacrificed through a percussive blow to the head and subsequent severing of the spinal cord with the approval of the institute’s animal welfare officer and in line with applicable regulations, i.e., the Directive on the Protection of Animals Used for Experimental or Other Scientific Purposes (’Verordnung zum Schutz von zu Versuchszwecken oder zu anderen wissenschaftlichen Zwecken verwendeten Tiere’–Tierschutz‐Versuchstierverordnung, Supporting Information 1) and the superordinate Directive 2010/63/EU (Supporting Information 2). Biomass projections were adjusted for the above biomass reduction through sampling. Furthermore, the fillet samples from the start and end of the trial were used for proximate composition analysis. Samples were stored at −80°C until further processing and final analyses. Mortalities were recorded daily.

The daily RAS operation included a 12% water exchange (125 L), which was the combined result of water loss through the drum filter wastewater, flushing of the settling tank, final removal of excess system water through the manual outlet valves according to volumetrically determined water level markings in the biofilter, and subsequent replenishment with fresh tap water. The water recirculation rate was kept at 600 L/h. Oxygen, pH, temperature (HQ40d, Hach Lange, Germany), and electrical conductivity (EC) (pH/Cond 740i, WTW, Germany) were measured in the fish tanks daily, and the RAS as well as the tap water were sampled weekly to determine dissolved element concentrations. From day 8, system water pH was stabilized at circumneutral levels by adding NaOH (≥99.5% purity, Carl Roth, Germany), which was dissolved in 1 L of deionized water and applied to the pump sumps via the drip applicators over a period of 12–18 h. The daily addition of NaOH was kept equal in all RAS and was increased from 10.5 to 18.9 g, totaling 446 g per RAS over the course of the trial.

The trial was conducted at the Leibniz Institute of Freshwater Ecology and Inland Fisheries in Berlin, Germany, from June to July 2024, by trained personnel according to German and European legislation on keeping and handling experimental animals. According to direct communication with the Ethics Committee of the Landesamt für Gesundheit und Soziales (LAGeSo), Berlin, Germany, this study was not categorized as an animal experiment requiring approval.

### 2.4. Sample Preparation and Analysis

#### 2.4.1. Experimental Diets

Proximate and amino acid compositions of the diets were determined at the accredited laboratory SGS Analytics, Germany, according to official standard methods [36–38]. Diets were freeze‐dried, homogenized in a mortar with liquid nitrogen, and afterwards redried before microwave‐assisted aqua regia digestion of a 150 mg dry sample with a ratio of HCl to HNO_3_ of 1:3 [39]. Elemental analysis (Ca, P, K, S, Na, Mg, Fe, Al, Zn, Mn, and Cu) was subsequently performed by inductively coupled plasma optical emission spectrometry (ICP‐OES) (iCAP 7400 ICP‐OES, Thermo Fisher Scientific, Waltham, MA, USA) [40].

#### 2.4.2. Water

Water samples (15 mL) were filtered (45 µm PVDF syringe filters; Sartorius, Germany) and fixed with 150 µL 2M HCl prior to determination of elemental concentrations (Ca, K, S, and Mg) with ICP‐OES [40] and concentrations of PO_4_‐P (soluble reactive phosphorus [SRP]), NH_4_‐N, NO_2_‐N, and NO_3_‐N (in sum referred to as total inorganic nitrogen [TIN]) with continuous flow analysis (FSR Seal High‐Resolution AA3 chemical analyzer, Seal Analytical, Germany).

#### 2.4.3. Fillet and Liver FAs

All matrices for FA analysis (fillet, liver, and feed) were freeze‐dried, homogenized with liquid nitrogen in a mortar, and again lyophilized to remove excess moisture. A subsample of 50 mg was taken for extraction and analysis. Prior to the above, the fillets (one entire fillet side per sampled fish) were deskinned and prehomogenized with a blender.

FA derivatization and extraction were performed according to DIN EN ISO 12966‐2:2017‐08 [41] with modifications as follows: for methylation, the acid catalyst 1 M H_2_SO_4_ was replaced by 1 M HCl, and the extractant isooctane was replaced by methyl *tert*‐butyl ether (MTBE), as presented in Schöning et al. [42]. Tricosanoic acid (C23:0) was used as an internal standard. Subsequently, gas chromatographic quantification of FAs was performed as described by Schöning et al. [42]. Supelco 37‐component FAME‐Mix (Merck, Germany) was used as the standard for calibration, and MassHunter software (Agilent Technologies, USA) was used for the detection of individual FAs and calculation of concentrations.

#### 2.4.4. Fillet Proximate Composition and Lipid Classes

Subsamples of the prehomogenized fillets from the start and the end of the trial were used for proximate composition (dry matter [DM], CP, total lipids, and ash) and lipid class analysis. DM content was determined by oven drying at 105°C for 8 h and ash content by incineration in a muffle furnace at 550°C for 6 h. For protein content, Kjeldahl analysis was performed according to standard methods [43], and total lipid content was determined by extraction of lipids with hexane‐isopropanol (3:2) according to Hara and Radin [44] with modifications outlined in Mráz and Pickova [45] followed by gravimetric quantification. Lipid class analysis was performed according to Olsen and Henderson [46] with modifications as described by Hematyar et al. [47].

#### 2.4.5. Liver Gene Expression

Subsamples (100 mg) of the freshly dissected livers were fixed in 1 mL of RNA later for 24 h at 4°C and, after removing the RNAlater, stored at −80°C. Total RNA was isolated according to Reiser et al. [48] using TRIzol (Invitrogen), followed by DNase I digestion (Invitrogen). After extraction, the total RNA content was determined with a Nanodrop ND 1000 spectrometer. RNA samples were diluted to 0.125 µg/µL. A premix of 8 µL RNA sample, 1 µL DNase I buffer (10×), and 1 U DNase was incubated for 15 min at room temperature before addition of 1 µL 25 mM EDTA and incubation for 10 min at 65°C. Integrity for all samples was monitored on a 1% agarose gel (40 V, 1–1.5 h) after 2 min of denaturation at 70°C. For 10% of the samples, RNA integrity was analyzed on an RNA 6000 Lab Chip using the Bioanalyzer as described in Kroupova et al. [49]. RIN averaged 8.58 ± 0.61, with all RIN meeting the minimum threshold >7.0. Reverse transcription was carried out with AffinityScript reverse transcriptase (200 U/µL, Agilent Technologies, USA). In brief, RNA (1 µg) was transcribed with 500 ng poly(dT) primer [CCTGAATTCTAGAGCTCA(T)17] in a two‐step procedure. First, annealing of poly(dT) primer at 65°C for 5 min, 40°C for 3 min, 35°C for 3 min, 25°C for 3 min, and 10°C for 5 min was carried out in 14 µL. Then, 2 µL buffer (10×), 2 µL DTT, 1 µL dNTP (10 mM each), and 1 µL Affinity Script reverse transcriptase were added, incubated at 42°C for 1 h, and denatured at 70°C for 15 min. Genomic DNA contamination was monitored by an RT control where the RT had been replaced by DEPC‐treated water.

Species‐specific primers (Table 4) were designed using the NCBI GenBank database. The specificity of the assays was confirmed by direct sequencing (SeqLab, Germany) and monitored by melting curve analysis. Realtime PCR was performed with a CFX qPCR Cycler (BioRad, Germany). For the qPCR reaction, 2 μL of the diluted samples (10 ng/μL) was used as a template in a 25 μL PCR mix containing YBR‐Green I (1.12×, Invitrogen), 2 mmol/L MgCl_2_, 200 μmol/L of each dNTP (Qbiogene, USA), and 400 nM of each primer, and 1 U Platinum Taq polymerase (Invitrogen). PCR was carried out with initial denaturation at 94°C for 2 min, followed by 40 cycles of denaturation at 94°C for 20 s, primer annealing at 63°C for 20 s, and elongation at 72°C for 20 s. PCR efficiencies were determined experimentally with a dilution series of a calibrator corresponding to 10 ng/μL, pooling 5 samples from 0 d. Each sample was assessed in duplicate. Expression of target genes was calculated by the comparative CT method (ΔΔCT) according to Pfaffl [50], corrected for the assay efficiencies, and normalized to *ef1a* (elongation factor 1α) as a housekeeping gene. The housekeeping gene was stably expressed across all groups and time points (Supporting Information 3: Figure S1). Gene expression is presented as a fold increase of the calibrator.

**Table 4:** Specifications of RT‐qPCR assays, including primer sequences, amplicon length, PCR efficiency (Eff.), and NCBI GenBank accession number of the respective housekeeping and target genes: *ef1a*—elongation factor 1α, *elovl2*—elongase of very long chain fatty acid 2, *elovl5*—elongase of very long chain fatty acid 5, and *fads2*—fatty acyl desaturase 2.

Gene	Primer	5′‐3′ sequence	Amplicon length (bp)	Eff.^a^ (%)	GenBank number
*ef1a*	f	CTgTgCTgATTgTTgCTggT	291	96.2	XM_053491388
	r	CCATATTTgTgCTgggCTCC			
*elovl2*	f	CgAggTTggATgCTACTggA	246	101.7	KU902414
	r	ACCCTCTCCTgCATCAAACA			
*elovl5*	f	CTTCCTgCACgTTTACCACC	208	96.2	AY660880
	r	CTggATCAgTTggCCTTgTg			
*fads2*	f	ACCTgggATgAAgTgCAgAA	204	92.2	XM_053478621
	r	CAgCggCTTCATgTACTTCC			

^a^Efficiency was determined from serial dilution series.

### 2.5. Calculations and Statistical Analyses

Formulas for calculating nitrogen free extract and gross energy of the diets as well as the various fish performance indices, including body weight gain (BWG), feed conversion ratio (FCR), specific growth rate (SGR), protein efficiency ratio (PER), thermal growth coefficient (TGC) and mean daily ration (MDR), are presented in the footnotes of the respective tables. FCR and PER were determined according to the amount of feed or CP, respectively, that each remaining individual consumed on average over the course of the trial, i.e., the sum of the individual average daily rations. This was done to control for the sampling of fish throughout the trial as well as the possible influence of unequal mortality.

In order to make feed‐related release of dissolved nutrients into the process water comparable between treatments, i.e., providing a measure of dissolved nutrient release per unit of feed, nutrient concentrations measured in the RAS were normalized to account for the effect of differing feed rations between the individual RAS and nutrient input through tap water, as previously outlined by Shaw et al. [51]. In short, the nutrient load introduced daily via fresh tap water as well as the nutrient load from the initial filling of the RAS with tap water was subtracted from the sum of the final nutrient load in the RAS water and the nutrient load that was removed daily from the RAS water through water exchange. The result is an entirely feed‐related nutrient load, which was then divided by feed input. The basis of the calculations were measured nutrient concentrations on sampling days and means between sampling days for days on which concentrations were not measured.

Data are shown as mean ± standard deviation, and one‐way analysis of variance (ANOVA) followed by a Bonferroni post‐hoc test for multiple comparisons was used to determine differences in treatment means. Given a significant Levene’s test, a Games–Howell post‐hoc test was applied for multiple comparisons. Statistical significance was set at *p* < 0.05. Calculations were performed with Microsoft Excel 2019, and statistical analyses were performed with IBM SPSS Statistics 22.0.

## 3. Results

### 3.1. Rearing and Growth Performance

Within reasonable limits, rearing conditions were comparable between treatments (Table 5). Mean water temperature was kept at 27.3°C, and oxygen levels in the rearing tanks were maintained between 5.5 and 7.4 mg/L (Supporting Information 3: Figures S2 and S3). Water pH was kept above 7 for the most part, with the HI and LO treatments ranging around 7.5 and the AC treatment experiencing a consistently lower pH range of 7.0–7.5 (Supporting Information 3: Figure S4). EC increased from 890 to 1330–1434 µS/cm, with the AC treatment showing continuously higher values than those of the other two treatments (Supporting Information 3: Figure S5). Mean NH_4_‐N and NO_2_‐N concentrations were 0.33–0.50 mg/L and 0.04–0.13 mg/L, respectively. Fish were initially stocked at 22–23 kg/m^3,^ and final rearing densities reached 55–65 kg/m^3^, while total water exchange amounted to 386–420 L/kg of feed.

**Table 5:** Experimental rearing conditions.

Parameters	Rearing conditions		
	AC	HI	LO
O_2_ (mg/L)^1^	6.56 ± 0.31	6.70 ± 0.25	6.69 ± 0.26
Temperature (°C)^1^	27.3 ± 0.4	27.3 ± 0.3	27.3 ± 0.2
pH^1^	7.41 ± 0.29	7.67 ± 0.17	7.69 ± 0.16
Conductivity (µS/cm)^1^	1218 ± 156	1182 ± 129	1192 ± 137
Final rearing density (kg/m^3^)^2^	64.8 ± 2.0	54.9 ± 1.1	54.6 ± 2.6
Water exchange (L/kg of feed)^2^	386 ± 12	418 ± 9	420 ± 10

*Note:* Values represent means ± standard deviations.

^1^Measured once daily before water exchange and feeding; *n* = 123.

^2^ *n* = 3.

At the start, there were no significant differences between dietary treatments with respect to initial biomass and mean individual weight, which similarly applied to survival and MDR over the course of the trial (Table 6). The AC diet resulted in significantly better growth performance compared to the HI and LO diets regarding all recorded measures, with 15%–17% higher final body weight and an FCR (DM basis) of 0.76 compared to 0.85–0.86, while notably, the HI and LO diets produced similar performance across all measures. Due to the better growth performance of the fish fed the AC diet, they subsequently received a significantly higher total feed ration compared to that of the other two experimental groups.

**Table 6:** Fish performance measures.

Parameters	Fish performance		
	AC	HI	LO
Survival (%)^A^	95.7 ± 2.1^a^	93.2 ± 1.1^a^	94.4 ± 1.9^a^
Mean initial body weight (g)^A^	144.9 ± 1.5^a^	141.9 ± 1.5^a^	139.2 ± 3.2^a^
Mean final body weight (g)^A^	462.4 ± 21.6^a^	402.5 ± 7.9^b^	394.6 ± 10.8^b^
BWG (g)^A^	317.5 ± 20.2^a^	260.7 ± 6.6^b^	255.4 ± 7.7^b^
BWG (%)^1A^	219 ± 11.9^a^	184 ± 3.0^b^	183 ± 1.8^b^
Initial biomass (g)^A^	7822 ± 83^a^	7661 ± 82^a^	7517 ± 173^a^
Final biomass (g)^A^	22,026 ± 676^a^	18,650 ± 372^b^	18,552 ± 882^b^
Total feed administered (g–as is)^A^	13,309 ± 419^a^	12,303 ± 258^b^	12,237 ± 290^b^
MDR (% biomass/d)^2B^	2.40 ± 0.75	2.46 ± 0.71	2.44 ± 0.76
FCR (as fed)^3A^	0.84 ± 0.02^b^	0.95 ± 0.00^a^	0.96 ± 0.02^a^
FCR (DM basis)^3A^	0.76 ± 0.02^b^	0.85 ± 0.00^a^	0.86 ± 0.02^a^
SGR^4A^	2.76 ± 0.09^a^	2.48 ± 0.03^b^	2.48 ± 0.02^b^
PER^5A^	2.75 ± 0.06^a^	2.50 ± 0.01^b^	2.47 ± 0.04^b^
TGC^6A^	2.17 ± 0.10^a^	1.89 ± 0.03^b^	1.88 ± 0.02^b^

*Note*: Values represent means ± standard deviations; means in rows with different superscript letters are significantly different (*p* < 0.05).

^A^ *n* = 3.

^B^ *n* = 123.

^1^BWG—mean body weight gain (%) = (final mean body weight [g] − initial mean body weight [g])/initial mean body weight (g)) × 100.

^2^MDR—mean daily ration (%/d) = sum (*r*_*i*_ × 100) / trial duration (days); where *i* = day of trial (1–42) such that *r*_*i*_ = ration (g)/biomass (g) of day *i*.

^3^FCR—feed conversion ration = total feed fed per individual (g as fed or DM) / [final mean body weight (g)−initial mean body weight (g)].

^4^SGR—specific growth rate = [ln (final mean body weight [g]) − ln (initial mean body weight [g])]/trial duration (days) × 100.

^5^PER—protein efficiency ratio = mean individual body weight gain (g) / CP fed per individual (g).

^6^TGC—thermal growth coefficient = 1000 × (final body weight [g]^1/3^ − initial body weight [g]^1/3^) × trial duration (days) × average temperature (°C) [52].

### 3.2. Dissolved Nutrient Release per Unit of Feed

Cumulatively over the course of the entire trial and corrected for the effects of water exchange and tap water nutrient loads, fish released significantly higher amounts of dissolved TIN per kg of feed with the AC diet compared to the other two diets, which did not differ in this regard (Table 7). On the contrary, the HI and LO diets produced significantly and substantially higher SRP release per kg of feed input, i.e., 1.8‐ and 2‐fold higher release, respectively, compared to the AC diet. However, fish fed the AC diet released slightly, yet significantly, more K than the HI and LO diets as well as significantly more S. The HI and LO diets resulted in the same release of N, P, Mg, S, and Ca per kg of feed and only differed significantly regarding K, with the LO diet producing a marginally but significantly higher release compared to the HI diet. Graphs of the cumulative dissolved nutrient release per kg of feed input for each sampled time point over the course of the trial can be found in the supplement (Supporting Information 3: Figure S6).

**Table 7:** Dissolved nutrient release per kg of feed input; corrected for the influence of daily water exchange and tap water nutrient introduction.

Nutrient (g/kg of feed)	Dissolved nutrient release		
	AC	HI	LO
TIN	32.50 ± 0.55^a^	25.63 ± 0.38^b^	27.24 ± 0.88^b^
SRP	0.98 ± 0.06^b^	1.80 ± 0.23^a^	1.97 ± 0.05^a^
K	3.45 ± 0.06^a^	3.05 ± 0.08^c^	3.24 ± 0.00^b^
Mg	1.06 ± 0.03^a^	1.08 ± 0.06^a^	1.12 ± 0.02^a^
S	4.14 ± 0.02^a^	2.84 ± 0.17^b^	3.05 ± 0.07^b^
Ca	3.13 ± 0.45^a^	3.42 ± 0.17^a^	3.79 ± 0.06^a^

*Note:* Values represent means ± standard deviations; means in rows with different superscript letters are significantly different (*p* < 0.05); *n* = 3.

### 3.3. Fillet Proximate Composition and Lipid Classes

The proximate composition of the fillet did not differ significantly between treatments, with values for protein, lipid, and ash content reaching 17.6%–18.4%, 6.9%–7.5%, and 1.1%, respectively (Table 8). Protein and ash content were similar between fish sampled at the start and the end of the trial. However, lipid content roughly doubled over the course of the trial, concomitant with an increase in fillet DM content from 21.5% to 25.1%–26% (Table 8, Supporting Information 3: Figure S7). Although the LO diet resulted in the lowest cholesterol levels and the highest phosphatidylethanolamine levels as a percentage of total lipids, the profile of major lipid classes in the fillet in terms of their relative proportions was nevertheless entirely nonsignificantly different.

**Table 8:** Fillet proximate composition as a percentage of wet weight and fillet lipid class profiles as a percentage of total lipids.

Composition	Fillet proximate composition and lipid classes			
	Start	AC	HI	LO
Proximate composition (% wet weight)				
DM	21.49 ± 0.62	25.57 ± 1.56	25.95 ± 0.82	25.06 ± 0.90
Protein	17.93 ± 0.59	18.43 ± 0.85	17.62 ± 0.85	18.28 ± 0.24
Total lipids	3.38 ± 0.60	6.87 ± 0.31	7.54 ± 1.38	6.85 ± 1.10
Ash	1.13 ± 0.04	1.12 ± 0.02	1.09 ± 0.01	1.09 ± 0.04
Lipid classes (% of total lipids)				
Triacylglycerole	—	55.13 ± 3.63	58.30 ± 3.17	55.04 ± 3.25
Cholesterol		16.04 ± 0.55	14.17 ± 0.15	12.19 ± 3.25
Phosphatidylethanolamine		13.91 ± 1.53	13.32 ± 1.82	15.39 ± 1.49
Phosphatidylcholine		11.29 ± 1.55	10.17 ± 0.77	12.60 ± 2.12
Phosphatidylinositol		0.91 ± 0.28	0.89 ± 0.27	0.76 ± 0.33
Cardiolipin		1.65 ± 0.11	1.58 ± 0.27	1.89 ± 0.26

*Note:* Values represent means ± standard deviations; means in rows with different superscript letters are significantly different (*p* < 0.05); *n* = 3.

### 3.4. Fillet and Liver FA

**Figure 1:**
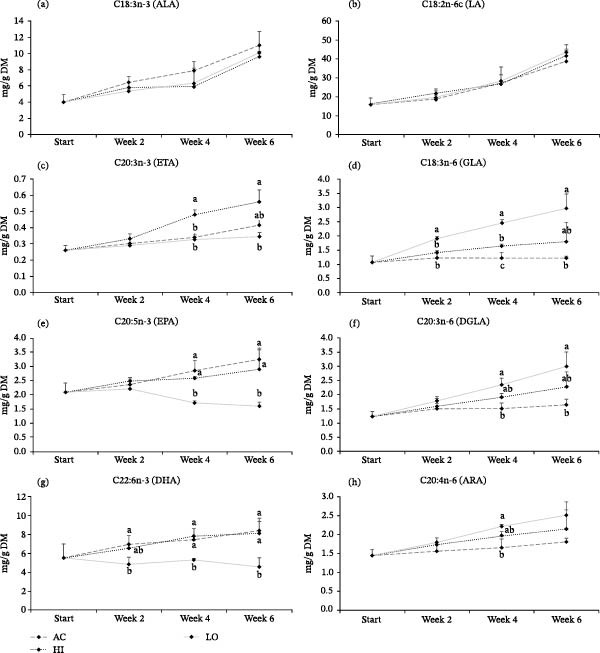
Development of measured fillet fatty acids involved in the LC‐PUFA biosynthesis pathway from ALA to EPA and DHA and from LA to ARA over the course of the trial. Values represent means ± standard deviations; different superscript letters indicate significant differences between group means per time point (*p* < 0.05); *n* = 3. (a) C18:3n−3 (ALA), (b) C18:2n−6c (LA), (c) C20:3n−3 (ETA), (d) C18:3n−6 (GLA), (e) C20:5n−3 (EPA), (f) C20:3n−6 (DGLA), (g) C22:6n−3 (DHA), and (h) C20:4n−6 (ARA).

**Figure 2:**
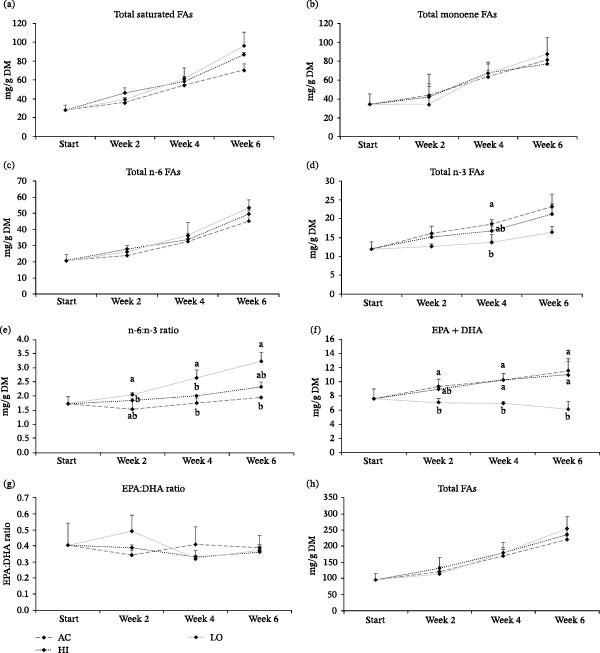
Development of fillet fatty acid groups and ratios over the course of the trial. Values represent means ± standard deviations; different superscript letters indicate significant differences between group means per time point (*p* < 0.05); *n* = 3. (a) Total saturated FAs, (b) total monoene FAs, (c) total n−6 FAs, (d) total n−3 FAs, (e) n−6:n−3 ratio, (f) EPA + DHA, (g) EPA:DHA ratio, and (h) total FAs.

In line with the approximate doubling of fillet total lipid content in all dietary treatments (Table 8), an increase in most FA was observed in the fillet over the course of the trial as well (Figures 1 and 2, Supporting Information 3: Table S1). Total FAs, total saturated, total monoene, and total n‐6 FAs in the fillet showed no significant differences (Figure 2). Although only significantly different compared to the n‐3 FA content in the fillet of the AC‐fed fish, total n‐3 FAs consistently accumulated to a lesser extent in the fillet of the fish fed the LO diet versus the other diets, reflecting the lower n‐3 FA content of the LO diet, particularly versus the AC diet. Similarly, the n‐6:n‐3 ratio in the fillet of the LO‐fed fish was consistently higher than the ratio found in the fillet of the HI‐ and AC‐fed fish, with ratios increasing from 1.74 to 3.24 (LO), 2.36 (HI), and 1.96 (AC) and approximately reaching the respective dietary n:6/n:3 ratios (LO: 3.02, HI: 2.54, AC: 2.06). Differences were significant compared to the HI‐fed fish in weeks 2 and 4 as well as compared to the AC‐fed fish in weeks 4 and 6.

The fillet ALA content followed a similar upward trajectory in all dietary treatments, and despite the ALA content of the LO diet being 74% higher compared to the HI diet (Table 3), the ALA content in the fillet was virtually equal between these two dietary groups throughout the entire trial (Figure 1). Roughly in accordance with the higher eicosatrienoic acid (ETA) content of the HI diet, the HI‐fed fish showed the highest fillet ETA content, which was significant compared to the fish fed the other two diets in week 4 and compared to the LO‐fed fish in week 6. EPA and DHA content in the fillet increased linearly by 38% and 55% over the course of the trial with the HI and AC diet with no significant differences, whereas the LO diet resulted in a significantly lower fillet EPA and DHA in week 4 and 6 as well as a 23% and 18% reduction versus the start of the trial. However, even though the fillet EPA and DHA content of the LO‐fed fish was only roughly half of the EPA and DHA content found in the fillet of the HI‐ and AC‐fed fish at the end of the trial, the difference in the EPA and DHA content of the LO diet versus the HI and the AC diet was considerably larger, with the LO diet only featuring a quarter to less than a fifth of the EPA and DHA content of the other two diets. Similar to EPA and DHA individually, the fillet EPA + DHA was significantly lower in the LO treatment versus the other two treatments, declining by 20% compared to the start of the trial, whereas the HI and AC treatments recorded a 44% and 53% increase (Figure 2). Again, despite the fillet EPA + DHA content produced by the LO diet at the end of the trial being only slightly more than half of what resulted from the HI and AC diets, the EPA + DHA content of the LO diet was merely 22% and 18% of the EPA + DHA content of the HI and AC diet, respectively. Fillet EPA:DHA ratios remained rather stable over time and did not differ between treatments. However, with final values of 0.36–0.39, they were notably lower than dietary EPA:DHA ratios (0.61–0.84).

Fillet LA content did not differ between dietary treatments over the course of the trial, whereas the LO diet produced the consistently highest GLA, DGLA, and ARA contents, with differences significant compared to the AC diet for GLA at all time points, for DGLA in weeks 4 and 6, and for ARA in week 4 (Figure 1). Although consistently higher, fillet GLA, DGLA, and ARA content resulting from the HI diet only differed significantly from the AC diet for GLA in week 4.

As a percentage of total measured fillet FAs, saturated FAs, monoene FAs, and n‐6 FAs (excl. ARA), and n‐3 FAs (excl. EPA + DHA) remained approximately stable across the duration of the trial in all dietary treatments, on average across all time points and treatments amounting to 34%, 35%, 20%, and 5% of total FAs, respectively (Supporting Information 3: Figure S8). However, considering the starting point of the trial with respect to EPA (2.2%), DHA (5.9%), EPA + DHA (8.1%), and ARA (1.5%), there was a clear and consistent trend towards a reduction of the above as a percentage of total FAs down to 0.6%–1.5%, 1.8%–3.8%, 4%–5.3%, and 0.8%–1.1% at the end of the trial, with the LO diet resulting in the lowest EPA, DHA, and EPA + DHA levels in this regard.

**Figure 3:**
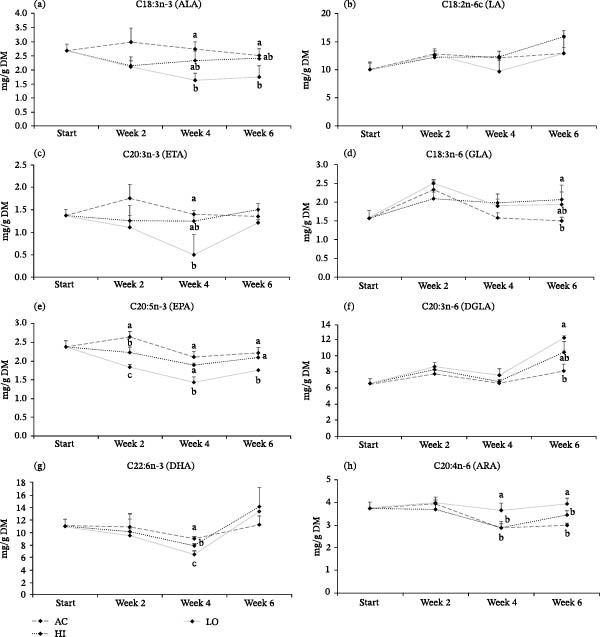
Development of measured liver fatty acids involved in the LC‐PUFA biosynthesis pathway from ALA to EPA and DHA, and from LA to ARA over the course of the trial. Values represent means ± standard deviations; different superscript letters indicate significant differences between group means per time point (*p* < 0.05); *n* = 3. (a) C18:3n−3 (ALA), (b) C18:2n−6c (LA), (c) C20:3n−3 (ETA), (d) C18:3n−6 (GLA), (e) C20:5n−3 (EPA), (f) C20:3n−6 (DGLA), (g) C22:6n−3 (DHA), and (h) C20:4n−6 (ARA).

**Figure 4:**
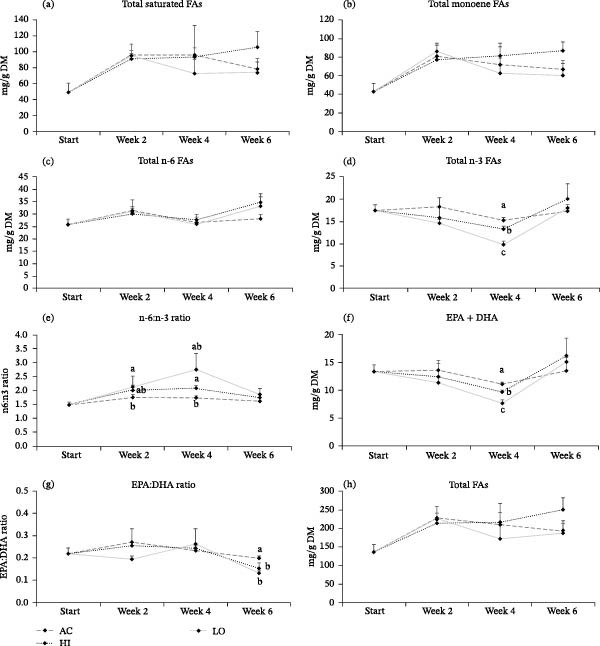
Development of liver fatty acid groups and ratios over the course of the trial. Values represent means ± standard deviations; different superscript letters indicate significant differences between group means per time point (*p* < 0.05); *n* = 3. (a) Total saturated FAs, (b) total monoene FAs, (c) total n−6 FAs, (d) total n−3 FAs, (e) n−6:n−3 ratio, (f) EPA + DHA, (g) EPA:DHA ratio, and (h) total FAs.

In contrast to the fillet, the liver did not show a general increase in the content of FAs throughout the trial and, for the most part, despite some significant differences between treatments for certain time points and FAs, featured fewer clear and consistent differences between treatments over the course of the trial (Figures 3 and 4, Supporting Information 3: Table S2). The most distinct and consistent differences were identified for EPA, with the LO diet resulting in significantly lower liver EPA content from week 2 onward compared to the other two diets, while ARA content was significantly highest in the liver of the LO‐fed fish from week 4 onward (Figure 3). In contrast, liver DHA content did not differ between the LO‐ and HI‐fed fish in weeks 2 and 6 and was only temporarily significantly lower in week 4 compared to the HI‐ and AC‐fed fish. The liver ALA and ETA content in W4 was significantly lower in fish fed the LO diet compared to fish fed the AC diet, but this difference only remained significant through the end of the trial for ALA. Similarly, the LO diet temporarily in week 4 led to a significantly lower total content of n‐3 FAs as well as EPA + DHA compared to the other diets (Figure 4). Liver EPA:DHA ratios only differed between treatments at the end of the trial, with the AC diet resulting in a significantly higher ratio (0.2) compared to the HI (0.15) and LO (0.13) diets. These ratios were even lower than those found in the fillet and, hence, also considerably lower than dietary EPA:DHA ratios (0.61–0.84).

### 3.5. Gene Expression—*elovl2*, *elovl5* and *fads2*

**Figure 5:**
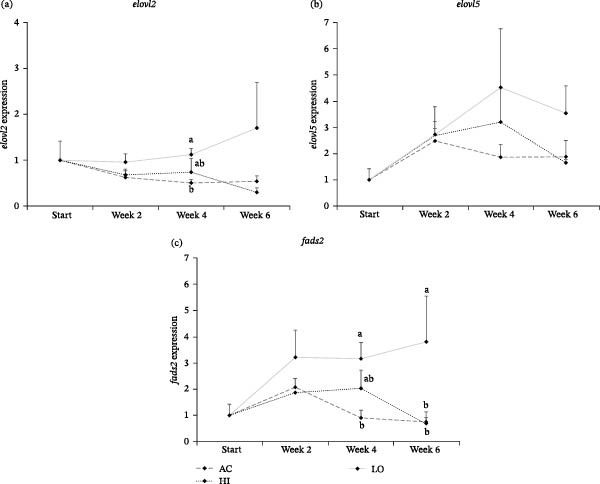
Expression of hepatic *elovl2* (a), *elovl5* (b), and *fads2* (c) genes over the course of the trial. Values represent means ± standard deviations; different superscript letters indicate significant differences between group means per time point (*p* < 0.05); *n* = 3.

Expression of *elovl2*, *elovl5*, and *fads2* in the liver was consistently highest in fish fed the LO diet from W2 onward and for *elovl5* from W4 onward (Figure 5). While expression of *elovl2* did not differ between the HI‐ and AC‐fed fish at any point and decreased to 30% and 54% of the expression level determined at the start of the trial, the LO treatment, in contrast, resulted in the consistently highest and a rising expression of *elovl2*, which was significantly higher compared to the AC treatment and finally reached an increase of 70% versus *elovl2* expression at the start of the trial.

The expression of *elovl5* reached its peak in week 2 for the AC treatment and in week 4 for the HI and LO treatments. Comparing the start and the end of the trial, the HI and the AC diets led to a moderate increase of *elovl5* expression (HI: + 65%, AC: + 87%), which was, however, considerably lower compared to the increase induced by the LO diet (+ 254%). However, despite higher *elovl5* expression in the LO treatment, particularly in weeks 4 and 6, differences were not significant. The LO diet on average led to a consistent rise of *fads2* expression, which was significant in week 4 compared to the AC diet and in week 6 compared to both of the other diets. The LO diet induced a substantial increase in *fads2* expression versus the start of the trial (+ 280%), whereas the HI and AC diets, after a temporary increase midway through the trial, actually led to a reduction of *fads2* expression (HI: −31%; AC: −27%). Considering the dynamics across the entire trial, overall trajectories of *elovl2* and *fads2* expression with the LO diet pointed upward, whereas they pointed downward with the HI and AC diet. Although pointing upward during the first part of the trial, trajectories of *elovl5* expression tended to point downward again during the latter part of the trial with all diets.

## 4. Discussion

Considering published information on growth and feed conversion performance of African catfish [53, 54], fish in the present trial overall performed reasonably well with FCRs of 0.84–0.96 (0.76–0.86 on a DM basis). Despite the initial objective of matching the growth performance of an isonitrogenous and isolipidic commercial diet (AC) with the circular ingredient‐based experimental diets (HI and LO), the growth performance of the commercial diet proved to be superior. This was potentially caused by the somewhat lower gross energy content of the experimental diets (18.8–18.9 vs. 20 MJ/kg) as well as the fact that their Met (0.65%) and Met + Cys content (1%) were lower compared to the AC diet (Met: 0.72% and Met + Cys: 1.36%) and slightly below requirements (IAFFD: Met 0.8% and Met + Cys 1.08%). Interestingly, despite the lower growth performance with the experimental diets, fish released 16% and 21% less dissolved TIN per unit of feed (HI: 25.6 g/kg of feed and LO: 27.2 g/kg of feed) compared to the commercial diet (AC: 32.5 g/kg), indicating lower amino acid catabolism and, in light of the lower growth performance, likely higher fecal N excretion, which together may have been the result of imbalances in the essential amino acid profile and/or lower amino acid digestibility. Due to the higher TIN release caused by the AC diet and the subsequently higher H^+^ production from the nitrification process in the biofilter, system water pH in the AC treatment was lower compared to that in the HI and LO treatment. Considering that the pH range experienced in all treatments during the trial is comfortably within the optimal pH range for African catfish culture [55], a confounding effect on growth performance is, however, unlikely. On the other hand, the higher phosphorus content of the experimental diets led to a release of SRP per unit of feed (HI: 1.80 g/kg of feed and LO: 1.97 g/kg of feed), roughly twice as high as the commercial diet (AC: 0.98 g/kg). Together with the lower TIN release, this improved the ratio of dissolved N and P release by the fish (HI/LO—10:0.7 vs. AC—10:0.3) in favor of a more suitable nutrient profile for hydroponic plant production in aquaponics with N:P ratios of 10:1.5–10:3 [56] and confirms prior work on optimizing the N:P release ratio of fish via purpose‐specific aquaponic feed formulation rich in P and thus the opportunity of reducing the dependence on mineral P fertilizer [33, 34]. Taking into account that system water pH levels in all treatments remained at a circumneutral level and oxygen levels were consistently high, the difference in pH between the AC treatment and the HI and the LO treatment is unlikely to have had a confounding effect on the results of the release of N or P by the fish caused by precipitation or solubilization [57]. Nevertheless, considering the suboptimal growth performance recorded in this trial for the experimental diets compared to the commercial diet, such circular ingredient‐based and high‐P diets would need to be fine‐tuned with respect to, e.g., their EAA profile, to fully exploit the maximum growth potential of African catfish.

Aside from the above‐discussed growth deficit caused by the experimental diets compared to the commercial diet, the most notable result regarding growth was that the fish performed equally well when fed an entirely marine‐ingredient‐free and low EPA + DHA diet based on rapeseed oil (EPA + DHA: 0.93 g/kg as fed) as when fed the same diet based on salmon oil with an almost 5 times higher EPA + DHA content (EPA + DHA: 4.26 g/kg as fed). This result is in line with previous studies on fish oil replacement with plant oils in African catfish that did not report a reduction in growth performance when entirely replacing fish oil with various plant oils [19–23, 27]. However, it should be noted that the rapeseed oil‐based LO diet of the present study reportedly or most likely had a considerably lower EPA + DHA content than the fish oil‐devoid diets tested in the above studies due to their substantial use of marine fish meal (23.2%–45% dietary inclusion) (e.g., [22, 27] with ca. 4.0–6.5 g EPA + DHA/kg feed). There have been studies suggesting that plant oil‐based diets outperform purely fish oil‐based diets high in n‐3 LC‐PUFAs. However, the fish oil control diets in these studies were almost devoid of n‐6 FAs with an n‐6:n‐3 ratio of 0.08 [25] or a similarly low ratio due to all dietary lipids originating from the added fish oil and the residual fish oil from the included fish meal [19]. This indicates, irrespective of the primary dietary oil source, that a certain balance between dietary n‐6 and n‐3 FAs is required for optimal growth, as previously suggested for African catfish [24, 25] and estimated to be in a range of 0.6–9.1 [22, 58]. All three experimental diets of the present study were well within this range (AC: 2.06, HI: 2.54, and LO: 3.02), making them well balanced in this respect.

Despite the similar growth performance resulting from the HI and the LO diets, as well as the comparably better growth resulting from the AC diet, fillet proximate composition and lipid classes did not differ significantly between diets, with overall fillet lipid content increasing from 3.4% to 6.9%–7.5% over the course of the trial with a concomitant increase of fillet DM content, while fillet protein and ash content remained unchanged. This is in line with other studies that did not find an effect of replacing fish oil with plant oils on the total muscle lipid content in African catfish [22, 25, 27]. The lipid content increase in the present study was accompanied by a consistent increase of total saturated, monoene, n‐6, and n‐3 FAs. Considering that the content of the above groups of FAs in the fillet of the fish at the start of the trial were all at or below their content in the feed (both on a DM basis) and at the end of the trial considerably above that dietary level, it indicates a substantial accumulation of these FAs by the fish. Nevertheless, the fillet FA profile for the most part remained approximately stable with regard to the proportions of saturated, monoene, and n‐6 (excl. ARA) and n‐3 (excl. EPA + DHA) FAs and roughly reflected the FA profile of the respective diets, with only saturated FAs being somewhat overrepresented in the fillet (AC: 31.9%, HI: 36.9%, and LO: 37.9%) compared to their proportion in the diets (AC: 22.6%, HI: 30.6%, and LO: 28.6%). The observation of the muscle FA profile depending strongly on the dietary FA profile confirms previous studies on African catfish [25, 27] as well as other fish species [59, 60]. Despite fillet EPA, DHA, and ARA content increasing for the fish fed the AC and the HI diet and fillet ARA content increasing for the fish fed the LO diet, fillet EPA, DHA, EPA + DHA, and ARA experienced a consistent reduction as a percentage of total FAs over the course of the trial across all dietary treatments. Although not determined in the present study, this may reflect the preferential accumulation of these functionally important FAs into neural, retinal, and reproductive tissues [61].

In general, fillet n‐6:n‐3 ratios also closely reflected dietary ratios (LO: 3.02, HI: 2.54, and AC: 2.06), with the rapeseed oil‐based LO diet resulting in the highest n‐6:n‐3 ratio (3.24), primarily caused by a significant reduction in EPA and DHA in contrast to the consistent increase in fillet EPA and DHA content recorded with the salmon oil‐based AC (1.96) and HI (2.36) diets. However, despite the LO diet only having 17%–26% of the EPA and DHA contents found in the AC and HI diets, fish fed the LO diet still featured half of the fillet EPA and DHA content of the fish fed the other two diets. At the same time, fillet ALA content was virtually identical across all dietary treatments, although the ALA content of the LO diet was 1.74 times higher than that of the HI diet. This, together with the consistently and partly substantially higher expression of hepatic *elovl2*, *elovl5*, and *fads2* in the LO‐fed fish, suggests not only a strong stimulation of the LC‐PUFA biosynthesis pathway from ALA to EPA and finally DHA on a gene expression level but also indicates actual and notable elongation and desaturation of ALA towards EPA and DHA at these low dietary EPA (0.42 g/kg as fed) and DHA (0.5 g/kg as fed) contents. Sourabié et al. [27] have also shown a significant increase in hepatic *elovl5* expression in African catfish fed an EPA‐ and DHA‐reduced fish meal and plant oil‐based diet compared to a fish meal and fish oil‐based diet. In contrast, hepatic *fads2* expression was not conclusively modulated in this study, perhaps caused by a still comparably high dietary EPA and DHA content from residual fish oil in the fish meal. In a follow‐up study, Sourabié et al. [22] demonstrated a significant increase in African catfish hepatic *elovl5* and *fads2* when replacing fish oil with desert date oil as well as shea butter, which corresponded with lower dietary EPA, DHA, and ARA levels in these diets compared to the fish oil control. This finding coincided with higher ARA levels in the muscle phospholipid fraction with the desert date oil as well as shea butter diets, leading the authors to link the heightened expression of *elovl5* and *fads2* to the conversion of n‐6 precursors such as LA into ARA. In fact, through functional characterization by heterologous expression of African catfish *elovl2*, *elovl5*, and *fads2* in yeast, Agaba et al. [62], Oboh et al. [18], and Oboh et al. [63] demonstrated that African catfish possess the entire multifunctional biosynthetic pathway to elongate and desaturate the n‐3 and n‐6 precursors ALA and LA towards EPA and ARA and further towards DHA and DPA (docosapentaenoic acid, C22:5n‐6) through the “Sprecher pathway” [64]. Considering the similarity in the content of ARA and other n‐6 FAs between all diets tested in the present study, results suggest that the higher expressions of hepatic *elovl2*, *elovl5*, and *fads2* in fish fed the rapeseed oil‐based LO diet were predominantly caused by the low EPA and DHA content of this diet and the need of the fish to cover their physiological requirements for these n‐3 FAs, i.e., that the n‐3 side of the LC‐PUFA biosynthetic pathway was stimulated. Interestingly, the liver of the LO‐fed fish, on the one hand, showed the consistently lowest EPA levels, concurrent with lower levels of its precursors ALA and ETA, although not as consistent as for EPA. On the other hand, hepatic DHA levels were similar across all dietary treatments, particularly when comparing the HI and LO diet. Since DHA tends to be functionally more relevant than EPA [9, 65, 66], the above may indicate that in light of the low EPA and particularly DHA content of the LO diets, ALA, ETA, and EPA were rapidly transformed towards DHA for urgent supply of tissues. Notably, fillet and especially liver EPA:DHA ratios were substantially lower than dietary EPA:DHA ratios across all treatments, pointing towards a higher demand and retention of DHA versus EPA, as has been clearly demonstrated for Atlantic salmon [65]. EPA, in turn, tends to be more readily β‐oxidized to cover cellular energy demand, transformed into eicosanoids with various physiological functions (e.g., control of inflammatory responses), or simply converted into DHA [61, 66].

While the complete replacement of salmon oil with rapeseed oil in an entirely marine‐ingredient‐free and low EPA + DHA diet did not affect the growth performance of African catfish, fillet FA composition and quality with respect to human nutritional value were altered significantly. The minimum daily EPA + DHA consumption recommended by the Food and Agricultural Organization of the United Nations to achieve nutraceutical benefits is given at 250 mg [67]. On the basis of a portion size of 100 g of fresh fillet, the salmon oil‐fed fish would be able to comfortably supply this recommended minimum with 298 mg (AC) and 286 mg (HI) EPA + DHA, whereas the rapeseed oil‐fed fish would only be able to provide 154 mg (LO). Considering that the fish at the end of the trial with a 395–462 g average weight were still considerably below the normal harvest size of 1–1.5 kg and fillet EPA + DHA content of the salmon oil‐fed fish was still on an upward trajectory, while it was stable if not decreasing in the rapeseed oil‐fed fish, the above EPA + DHA quality gap would likely even widen if continuing grow‐out with the same diets. For a healthy human diet, recommended n‐6:n‐3 ratios to reduce the risk of diet‐related chronic inflammatory diseases, certain cancers, and cardiovascular diseases lie between 1–2 and 4–5 [68, 69]. With ratios of 1.96 (AC), 2.36 (HI), and 3.24 (LO), fish fed all three diets produced fillets within or close to these recommended optimal ratio ranges, whereas again, the fillet from the salmon oil‐fed fish would be considered superior to the rapeseed oil‐fed fish in this respect. However, taking into account that there was no negative effect on growth performance with the marine‐ingredient‐free and rapeseed oil‐based LO diet, at least at the tested EPA + DHA level (0.93 g/kg as fed) and n‐6:n‐3 ratio (3.02) over the course of the 6‐week trial, this could present the opportunity of replacing or at least reducing scarce and high‐priced fish oils and other n‐3 LC‐PUFA sources (e.g., microalgae, transgenic oilseeds) in diets for African catfish. The duration of the present trial, however, covered only part of the grow‐out phase, and fish may still have benefited from EPA and DHA deposits acquired before the start of the trial, which is especially relevant in the case of the LO diet. Therefore, a conclusive evaluation of this opportunity would require longer‐term grow‐out trials with perhaps even lower dietary EPA + DHA levels to judge the effect of minimal EPA + DHA intake on the growth performance, health status, and n‐3 LC‐PUFA biosynthetic capacity of African catfish. In any case, integrating a generally more judicious use of EPA and DHA through the utilization of low EPA and DHA circular protein sources and, e.g., cheaper rapeseed oil, with a finishing feed approach that employs diets with higher EPA + DHA content during a limited period before harvest could be beneficial in terms of balancing sustainability and economic concerns with human nutritional aspects. While finishing feed strategies have been investigated in various important fish species such as, for instance, tilapia [70, 71], common carp [13], and rainbow trout [72, 73], such strategies still need to be investigated for African catfish as a high‐potential aquaculture species.

## 5. Conclusion

In summary, this study investigated the effect of replacing EPA‐ and DHA‐rich salmon oil with EPA‐ and DHA‐devoid rapeseed oil in an otherwise entirely marine‐ingredient‐free experimental diet compared to an industrial control diet on the growth performance, dissolved nutrient excretion, and FA profile development in fillet and liver, as well as hepatic gene expression development (*elovl2*, *elovl5*, and *fads2*) of African catfish (*Clarias gariepinus*).

i. While the growth performance of the industrial control diet was not matched, the circular animal protein‐based experimental diets confirmed previous studies with regard to the ability of improving the ratio of dissolved nitrogen to phosphorus for the aquaponic use case. The replacement of salmon oil with rapeseed oil did not affect the growth performance.
ii. However, replacement of salmon oil with rapeseed oil significantly altered the content of specific n‐3 and n‐6 LC‐PUFAs, particularly in the fillet but also in the liver of the fish, and stimulated the hepatic expression of all genes involved in the biosynthetic pathway of LC‐PUFAs from C_18_ precursors (*elovl2*, *elovl5*, and *fads2*). Considering the similar n‐6 FA profile of the diets and the minimal EPA + DHA content of the rapeseed oil diet, findings clearly point to the activation of the n‐3 side of this biosynthetic pathway.
iii. Although integrated examination of gene expression and individual n‐3 FA accumulation in fillet and liver suggests active biosynthesis of EPA and DHA in the rapeseed oil‐fed fish, resulting fillets did not achieve the same human nutritional quality as fillets from salmon oil‐fed fish in terms of FA profile (EPA + DHA, n‐6:n‐3 ratio). Nevertheless, the lack of an effect on growth indicates the potential of reducing EPA and DHA levels in African catfish diets while leaving open the possibility of improving human nutritional quality through finishing feed approaches. In this sense, strategies should be identified to optimally balance economic and sustainability considerations of dietary EPA + DHA reduction on the side of fish farmers and feed producers with the species’ requirements and final product quality.

## Author Contributions

**Christopher Shaw**: writing – original draft, writing – review and editing, visualization, methodology, investigation, formal analysis, data curation, conceptualization. **Klaus Knopf**: writing – review and editing, supervision, resources, conceptualization. **Sven Wuertz, Koushik Roy, and Radek Gebauer:** writing – review and editing, methodology, resources, conceptualization. **Tobias Goldhammer**: writing – review and editing, resources, supervision. **Viola Schöning**: writing – review and editing, data curation, methodology, investigation. **Wibke Kleiner and Marvin Sens:** methodology, data curation. **Christian Ulrichs**: writing – review and editing, project administration, funding acquisition. **Werner Kloas**: writing – review and editing, supervision, resources, project administration, funding acquisition, conceptualization.

## Funding

This research was funded by the Federal Ministry of Research, Technology, and Space as part of Agricultural Systems of the Future (CUBES Circle) (Grant 031B1523B). Part of the work was carried out with the support of the VVI CENAKVA Research Infrastructure (Grant ID 90238, MEYS CR, 2023–2026). Open Access funding enabled and organized by Projekt DEAL.

## Ethics Statement

This study did not require an application for approval according to communication with the Ethics Committee of the Landesamt für Gesundheit und Soziales (LAGeSo), Berlin.

## Conflicts of Interest

The authors declare no conflicts of interest.

## Supporting Information

Additional supporting information can be found online in the Supporting Information section.

## Supporting information


**Supporting Information 1** Verordnung zum Schutz von zu Versuchszwecken oder zu anderen wissenschaftlichen Zwecken verwendeten Tiere’– Tierschutz‐Versuchstierverordnung (Anlage 2) https://www.gesetze-im-internet.de/tierschversv/TierSchVersV.pdf.

**Supporting Information 2** Directive 2010/63/EU (Annex 4) https://eur-lex.europa.eu/legal-content/EN/TXT/PDF/?uri=CELEX:32010L0063.

**Supporting Information 3** Figure S1: Expression of the housekeeping gene *ef1a* (elongation factor 1α) over the course of the trial. Figure S2: Development of RAS water temperature over the course of the trial. Figure S3: Development of RAS water oxygen concentration over the course of the trial. Figure S4: Development of RAS water pH over the course of the trial. Figure S5: Development of RAS water electrical conductivity over the course of the trial. Figure S6: Cumulative dissolved nutrient release per kg of feed input over the course of the trial; corrected for the influence of daily water exchange and tap water nutrient introduction. (A) Total inorganic nitrogen (TIN), (B) Soluble reactive phosphorus (SRP), (C) Potassium (K), (D) Magnesium (Mg), (E) Calcium (Ca), and (F) Sulfur (S). Figure S7: Development of fillet dry matter content (% wet weight) over the course of the trial. Figure S8: Relative proportion of fatty acid (FA) groups and certain FAs as a percentage (%) of total measured FAs in the fillet of the fish. Table S1: Fillet fatty acid content for week 2, 4, and 6 of the trial (g/kg DM). Table S2: Liver fatty acid content for week 2, 4, and 6 of the trial (g/kg DM).

## Data Availability

The data are available online via the following link: https://doi.org/10.6084/m9.figshare.30833891 (accessed on December 15, 2025).
