# Supplementary material for: Compensatory Fatty Acid Metabolism and Hepatic Gene Expression in African Catfish (Clarias gariepinus) Fed Marine‐Ingredient‐Free Circular Diets Low in EPA and DHA
Source: Aquac Nutr. 2026 Jun 11;2026:4162985. doi: 10.1155/anu/4162985 (PMC13259715; doi:10.1155/anu/4162985)
Supplement: Supplementary file 1 — Supporting Information 1 Verordnung zum Schutz von zu Versuchszwecken oder zu anderen wissenschaftlichen Zwecken verwendeten Tiere’– Tierschutz‐Versuchstierverordnung (Anlage 2) https://www.gesetze-im-internet.de/tierschversv/TierSchVersV.pdf. [file ANU-2026-4162985-s002.pdf]

Verordnung zum Schutz von zu Versuchszwecken oder zu anderen wissenschaftlichen Zwecken verwendeten Tieren (Tierschutz-Versuchstierverordnung - TierSchVersV)

TierSchVersV

Ausfertigungsdatum: 01.08.2013

Vollzitat:

"Tierschutz-Versuchstierverordnung vom 1. August 2013 (BGBl. I S. 3125, 3126), die zuletzt durch Artikel 1 der Verordnung vom 10. Oktober 2025 (BGBl. 2025 I Nr. 238) geändert worden ist"

Stand: Zuletzt geändert durch Art. 1 V v. 10.10.2025 I Nr. 238

Fußnote

```
(+++ Textnachweis ab: 13.8.2013 +++)


(+++ Zur Anwendung vgl. § 48 +++)


(+++ Amtlicher Hinweis des Normgebers auf EG-Recht:
   Umsetzung der
     EURL 63/2010    (CELEX Nr: 32010L0063) vgl. V v. 11.8.2021 I 3570
   Umsetzung der
     EURL 2024/1262  (CELEX Nr: 32024L1262) vgl. V v. 10.10.2025 I Nr. 238 +++)
```

Die V wurde als Artikel 1 der V v. 1.8.2013 I 3125 vom Bundesministerium für Ernährung, Landwirtschaft und Verbraucherschutz nach Anhörung der Tierschutzkommission im Einvernehmen mit den Bundesministerien für Bildung und Forschung, für Umwelt, Naturschutz und Reaktorsicherheit, für Verkehr, Bau und Stadtentwicklung, für Wirtschaft und Technologie und der Verteidigung, mit Zustimmung des Bundesrates beschlossen. Sie ist gem. Art. 4 dieser V am 13.8.2013 in Kraft getreten.

Inhaltsübersicht

Abschnitt 1
Halten von
Wirbeltieren und Kopffüßern zur
Verwendung in Tierversuchen oder
zu anderen wissenschaftlichen Zwecken
Unterabschnitt 1
Anforderungen an die Haltung
sowie an Einrichtungen und Betriebe

§ 1 Anforderungen an die Haltung von Wirbeltieren und Kopffüßern
§ 2 Anforderungen an die Tötung von Wirbeltieren und Kopffüßern
§ 3 Anforderungen an die Sachkunde
§ 4 Organisationspflichten
§ 5 Tierschutzbeauftragte
§ 6 Tierschutzausschuss
§ 7 Führen von Aufzeichnungen

§ 8 Besondere Aufzeichnungen bei Hunden, Katzen und Primaten
§ 9 Kennzeichnung von Hunden, Katzen und Primaten
§ 10 Anderweitige Unterbringung oder Freilassung von Wirbeltieren und Kopffüßern

Unterabschnitt 2
Erlaubnis nach § 11 Absatz 1
Satz 1 Nummer 1 des Tierschutzgesetzes

§ 11 Erlaubnisvoraussetzungen
§ 12 Beantragen der Erlaubnis
§ 13 Erlaubnisbescheid, Anzeige und Erlaubnis von Änderungen

Abschnitt 2
Durchführung, Genehmigung
und Anzeige von Tierversuchen

§ 14 Geltung für Tiere in einem frühen Entwicklungsstadium

Unterabschnitt 1
Durchführung von Tierversuchen

§ 15 Anforderungen an Räumlichkeiten und Anlagen
§ 16 Anforderungen an die Sachkunde
§ 17 Schmerzlinderung und Betäubung
§ 18 Erneutes Verwenden von Wirbeltieren und Kopffüßern
§ 19 Verwenden gezüchteter Wirbeltiere und Kopffüßer
§ 20 Verwenden wildlebender Tiere
§ 21 Verwenden herrenloser oder verwilderter Haustiere
§ 22 Verwenden geschützter Tierarten
§ 23 Verwenden von Primaten
§ 24 Herkunft zu verwendender Primaten
§ 25 Durchführung besonders belastender Tierversuche
§ 26 Genehmigungen in besonderen Fällen
§ 27 Zweckerreichung
§ 28 Verfahren nach Abschluss, Nachbehandlung
§ 29 Führen von Aufzeichnungen zu Tierversuchen
§ 30 Pflichten des Leiters

Unterabschnitt 2
Genehmigung und Anzeige von Versuchsvorhaben

§ 31 Beantragen der Genehmigung

§ 32 Genehmigungsverfahren, Bearbeitungsfristen

§ 33 Genehmigungsbescheid, Befristung

§ 34 Genehmigung und Anzeige von Änderungen genehmigter Versuchsvorhaben

§ 35 Rückblickende Bewertung von Versuchsvorhaben

§ 36 Vereinfachtes Genehmigungsverfahren für Versuchsvorhaben nach § 8a Absatz 1 des Tierschutzgesetzes

§ 37 Sammelgenehmigung und Genehmigung von Änderungen genehmigter Versuchsvorhaben im vereinfachten Genehmigungsverfahren

§ 38 Prüfung der Anzeige von Änderungen von Versuchsvorhaben

§ 39 Anzeige von Versuchsvorhaben an Zehnfußkrebsen

§ 40 Aufbewahrungspflicht

§ 41 Veröffentlichung von Zusammenfassungen

§ 42 Tierversuchskommissionen

§ 43 Unterrichtung des Bundesministeriums

Abschnitt 3
Ordnungswidrigkeiten

§ 44 Ordnungswidrigkeiten

Abschnitt 4
Übergangs- und Schlussbestimmungen

§ 45 Aufgaben nach Artikel 49 der Richtlinie 2010/63/EU

§ 46 Beratung zu Alternativen zu Tierversuchen

§ 47 Unberührtheitsklausel

§ 48 Übergangsvorschriften

Abschnitt 1
Halten von Wirbeltieren und Kopffüßern zur Verwendung in Tierversuchen oder zu anderen wissenschaftlichen Zwecken

Unterabschnitt 1
Anforderungen an die Haltung sowie an Einrichtungen und Betriebe

§ 1 Anforderungen an die Haltung von Wirbeltieren und Kopffüßern

(1) Der Leiter einer Einrichtung oder der Verantwortliche für einen Betrieb, in der oder in dem Wirbeltiere oder Kopffüßer gehalten werden, die zur Verwendung in Tierversuchen bestimmt sind oder deren Gewebe oder Organe dazu bestimmt sind, zu wissenschaftlichen Zwecken verwendet zu werden, hat sicherzustellen, dass

1. die Haltung der Tiere, auch während ihrer Verwendung in Tierversuchen, den sich aus Anhang III der Richtlinie 2010/63/EU des Europäischen Parlaments und des Rates vom 22. September 2010 zum Schutz der für wissenschaftliche Zwecke verwendeten Tiere (ABl. L 276 vom 20.10.2010, S. 33) in der jeweils geltenden Fassung ergebenden Anforderungen entspricht,
2. mindestens einmal täglich das Befinden der Tiere durch direkte Inaugenscheinnahme und die Haltungsbedingungen sowie die Funktionsfähigkeit der der Haltung dienenden Anlagen durch geeignete Maßnahmen überprüft werden,

3. die Tiere so befördert werden, dass ihnen keine vermeidbaren Schmerzen, Leiden oder Schäden zugefügt werden,
4. unverzüglich Abhilfe geschaffen wird, sobald festgestellt wird, dass die in Nummer 1 bis 3 genannten Anforderungen nicht eingehalten werden oder den Tieren vermeidbare Schmerzen, Leiden oder Schäden zugefügt werden, und
5. die Haltung der Tiere, auch während ihrer Verwendung in einem Tierversuch, fortlaufend hinsichtlich der Möglichkeiten zur Verbesserung des Wohlergehens der Tiere überprüft wird.

Soweit Artikel 33 Absatz 2 in Verbindung mit Anhang III der Richtlinie 2010/63/EU bestimmt, dass Anforderungen ab einem dort genannten Zeitpunkt angewendet werden, ist Satz 1 Nummer 1 ab dem dort genannten Zeitpunkt anzuwenden. Die Sätze 1 und 2 gelten auch für Einrichtungen und Betriebe, in denen die dort genannten Tiere gezüchtet oder zum Zwecke der Abgabe an Dritte gehalten werden.

(2) Die zuständige Behörde kann Ausnahmen von den Anforderungen nach Absatz 1 Satz 1 Nummer 1 genehmigen, soweit

1. wissenschaftlich begründet dargelegt ist, dass dies im Hinblick auf den Zweck des Tierversuchs unerlässlich ist, oder
2. dies aus Gründen des Tierschutzes oder der Tiergesundheit erforderlich ist.

(3) Anhang A des Europäischen Übereinkommens vom 18. März 1986 zum Schutz der für Versuche und andere wissenschaftliche Zwecke verwendeten Wirbeltiere bleibt unberührt.

§ 2 Anforderungen an die Tötung von Wirbeltieren und Kopffüßern

(1) In § 1 Absatz 1 bezeichnete Wirbeltiere und Kopffüßer dürfen nur

1. in den Räumlichkeiten einer Einrichtung oder eines Betriebs im Sinne des § 1 Absatz 1,
2. von einer Person, die die Anforderungen nach Anlage 1 Abschnitt 2 erfüllt, und
3. unter Betäubung oder sonst nur unter größtmöglicher Vermeidung von Schmerzen und Leiden

getötet werden. Räumlichkeiten im Sinne des Satzes 1 Nummer 1 sind auch bewegliche oder lediglich teilweise umschlossene oder überdachte Örtlichkeiten. Satz 1 Nummer 1 gilt nicht, wenn ein Tier im Rahmen eines Tierversuchs getötet wird, der nach § 15 Absatz 1 Satz 3 außerhalb einer Einrichtung oder eines Betriebs durchgeführt wird. Satz 1 Nummer 2 gilt nicht, wenn ein Tier im Rahmen eines Tierversuchs getötet wird, der Ausbildungs-, Fortbildungs- oder Weiterbildungszwecken dient, soweit das Töten in Anwesenheit und unter Aufsicht einer Person erfolgt, die die nach Satz 1 Nummer 2 erforderlichen Anforderungen erfüllt.

(2) In § 1 Absatz 1 bezeichnete Wirbeltiere und Kopffüßer dürfen darüber hinaus nur nach Maßgabe der Anlage 2 getötet werden, wobei das Verfahren anzuwenden ist, das

1. für das Tier die geringste Belastung bedeutet und
2. mit dem Versuchszweck vereinbar ist.

Satz 1 gilt nicht für das Töten von Tieren,

1. die empfindungs- und wahrnehmungslos sind, sofern sie vor dem Tod ihre Wahrnehmungs- und Empfindungsfähigkeit nicht wiedererlangen und bis zur sicheren Feststellung des Todes des Tieres eine Kontrolle der Wahrnehmungs- und Empfindungslosigkeit erfolgt, oder
2. die in der landwirtschaftlichen Forschung verwendet werden, wenn der Zweck des Versuchsvorhabens es erforderlich macht, dass die Tiere unter vergleichbaren Bedingungen wie in der Nutztierhaltung zu Erwerbszwecken gehalten werden und sie im Einklang mit den Anforderungen des Anhangs I der Verordnung (EG) Nr. 1099/2009 des Rates vom 24. September 2009 über den Schutz von Tieren zum Zeitpunkt der Tötung (ABl. L 303 vom 18.11.2009, S. 1) in der jeweils geltenden Fassung getötet werden.

(3) Die zuständige Behörde kann die Anwendung eines den Anforderungen des Absatzes 2 Satz 1 nicht entsprechenden Tötungsverfahrens genehmigen, wenn

1. dieses Verfahren wissenschaftlichen Erkenntnissen zufolge nicht mit stärkeren Schmerzen und Leiden verbunden ist als ein den Anforderungen entsprechendes Verfahren oder

2. im Falle der Tötung eines Tieres im Rahmen seiner Verwendung in einem Tierversuch wissenschaftlich begründet dargelegt ist, dass die Anwendung dieses Verfahrens im Hinblick auf den Zweck des Tierversuchs unerlässlich und ethisch vertretbar ist.

§ 3 Anforderungen an die Sachkunde

(1) Der Leiter einer Einrichtung oder der Verantwortliche für einen Betrieb im Sinne des § 1 Absatz 1 hat sicherzustellen, dass

1. die mit der Pflege der Tiere betrauten Personen über die Kenntnisse und Fähigkeiten nach Anlage 1 Abschnitt 1 und
2. die mit dem Töten der Tiere betrauten Personen über die Kenntnisse und Fähigkeiten nach Anlage 1 Abschnitt 2

verfügen. Satz 1 gilt nicht für Personen, die die dort genannten Tätigkeiten zu Ausbildungs-, Fortbildungs- oder Weiterbildungszwecken in Anwesenheit und unter Aufsicht einer Person mit den nach Satz 1 Nummer 1 oder 2 erforderlichen Kenntnissen und Fähigkeiten ausüben.

(2) Der nach Absatz 1 Satz 1 Verpflichtete hat außerdem sicherzustellen, dass sich Personen nach Absatz 1 Satz 1 im Hinblick auf die dort genannten Kenntnisse und Fähigkeiten und Personen, die in der Einrichtung oder dem Betrieb mit der Durchführung von Tierversuchen an Wirbeltieren oder Kopffüßern betraut sind, im Hinblick auf die nach § 16 Absatz 1 Satz 1 erforderlichen Kenntnisse und Fähigkeiten regelmäßig fortbilden.

§ 4 Organisationspflichten

Für Einrichtungen und Betriebe im Sinne des § 10 Absatz 1 Satz 1 und 2 des Tierschutzgesetzes hat der Träger der Einrichtung oder der für den Betrieb Verantwortliche eine oder mehrere Personen vor Ort zu bestellen, die

1. für die Überwachung der Pflege der in der Einrichtung oder in dem Betrieb befindlichen Tiere und ihr Wohlergehen verantwortlich sind,
2. gewährleisten, dass Personen, die mit den Tieren umgehen, Zugang zu Informationen über die in der Einrichtung oder in dem Betrieb untergebrachten Tierarten erhalten, und
3. dafür sorgen, dass
    a) die Personen, die mit Aufgaben im Bereich der Pflege oder dem Töten der Tiere betraut sind, die Anforderungen des § 3 Absatz 1 und
    b) die Personen, die Tierversuche durchführen, die Anforderungen des § 7 Absatz 1 Satz 4 des Tierschutzgesetzes und des § 16

    erfüllen, diesbezüglich fortlaufend geschult werden und solange beaufsichtigt werden, bis die erforderlichen Fähigkeiten in der Praxis nachgewiesen worden sind.

§ 5 Tierschutzbeauftragte

(1) Für Einrichtungen und Betriebe im Sinne des § 10 Absatz 1 des Tierschutzgesetzes hat der Träger der Einrichtung oder der für den Betrieb Verantwortliche vor Aufnahme der Tätigkeit einen oder mehrere Tierschutzbeauftragte zu bestellen und die Bestellung der zuständigen Behörde anzuzeigen. In der Anzeige sind entsprechend den Anforderungen des Absatzes 6 Satz 3 auch die Stellung und die Befugnisse des Tierschutzbeauftragten anzugeben.

(2) Der Tierschutzbeauftragte darf nicht zugleich die für das Züchten oder Halten der Tiere verantwortliche Person im Sinne des § 11 Absatz 1 Satz 1 Nummer 1 sein. Die zuständige Behörde kann Ausnahmen zulassen, soweit dies auf Grund der sachlichen und personellen Ausstattung der Einrichtung oder des Betriebs sachgerecht ist und Belange des Tierschutzes nicht entgegenstehen. Führt ein Tierschutzbeauftragter einer Einrichtung oder eines Betriebs, in der oder in dem Tierversuche durchgeführt werden, selbst ein Versuchsvorhaben durch, so muss für dieses Versuchsvorhaben ein anderer Tierschutzbeauftragter tätig sein.

(3) Zum Tierschutzbeauftragten können nur Personen mit abgeschlossenem Hochschulstudium der Veterinärmedizin bestellt werden. Sie müssen die für die Durchführung ihrer in Absatz 4 bezeichneten Aufgaben erforderlichen Kenntnisse und Fähigkeiten und die hierfür erforderliche Zuverlässigkeit haben. Der Tierschutzbeauftragte ist verpflichtet, die für seine Aufgaben erforderlichen Kenntnisse und Fähigkeiten durch

regelmäßige Fortbildungen auf dem Stand von Wissenschaft und Technik zu halten. Die zuständige Behörde kann Ausnahmen von Satz 1 genehmigen, wenn

1. die Bestellung einer anderen spezialisierten Person geeigneter ist als die Bestellung einer Person mit einem abgeschlossenen Hochschulstudium der Veterinärmedizin und
2. die Person die nach Satz 2 erforderlichen Kenntnisse und Fähigkeiten nachgewiesen hat.

(4) Der Tierschutzbeauftragte ist verpflichtet,

1. auf die Einhaltung von Vorschriften, Bedingungen und Auflagen im Interesse des Tierschutzes zu achten und
2. die Einrichtung oder den Betrieb und die mit der Haltung der Tiere befassten Personen zu beraten, insbesondere hinsichtlich des Wohlergehens der Tiere und der Möglichkeiten zur Verbesserung des Wohlergehens der Tiere beim Erwerb, der Unterbringung und der Pflege sowie hinsichtlich deren medizinischer Behandlung.

Der Tierschutzbeauftragte einer Einrichtung oder eines Betriebs, in der oder in dem Tierversuche durchgeführt werden, ist darüber hinaus verpflichtet

1. zu jedem Antrag auf Genehmigung eines Versuchsvorhabens Stellung zu nehmen und diese Stellungnahme der zuständigen Behörde auf Verlangen vorzulegen,
2. innerbetrieblich auf die Entwicklung und Einführung von Verfahren und Mitteln zur Erfüllung der Anforderungen des § 7 Absatz 1 Satz 2 und des § 7a Absatz 2 Nummer 2, 4 und 5 des Tierschutzgesetzes hinzuwirken und
3. die mit der Durchführung von Tierversuchen befassten Personen insbesondere im Hinblick auf die Anwendung der in Nummer 2 genannten Verfahren und Mittel zu beraten und diese laufend über diesbezügliche technische und wissenschaftliche Entwicklungen zu informieren.

(5) Die Einrichtung oder der Betrieb hat den Tierschutzbeauftragten

1. bei der Erfüllung seiner Aufgaben so zu unterstützen, dass er seine Aufgaben uneingeschränkt wahrnehmen kann, und
2. in den Fällen des Absatzes 4 Satz 2 von allen Versuchsvorhaben zu unterrichten.

Sie haben sicherzustellen, dass sich der Tierschutzbeauftragte regelmäßig fortbildet.

(6) Der Tierschutzbeauftragte ist bei der Erfüllung seiner Aufgaben weisungsfrei. Er darf wegen der Erfüllung seiner Aufgaben nicht benachteiligt werden. Seine Stellung und seine Befugnisse sind durch Satzung, innerbetriebliche Anweisung oder in ähnlicher Form zu regeln. Dabei ist sicherzustellen, dass der Tierschutzbeauftragte seine Vorschläge oder Bedenken unmittelbar der in der Einrichtung oder in dem Betrieb entscheidenden Stelle vortragen kann. Werden mehrere Tierschutzbeauftragte bestellt, so sind ihre Aufgabenbereiche festzulegen.

§ 6 Tierschutzausschuss

(1) Für Einrichtungen und Betriebe im Sinne des § 10 Absatz 1 Satz 1 und 2 des Tierschutzgesetzes hat der Träger der Einrichtung oder der für den Betrieb Verantwortliche vor Aufnahme der Tätigkeit einen Tierschutzausschuss zu bestellen. Dem Tierschutzausschuss gehören mindestens an

1. die für die Überwachung der Pflege der in der Einrichtung oder in dem Betrieb befindlichen Tiere und ihr Wohlergehen verantwortlichen Personen und
2. ein wissenschaftliches Mitglied, soweit in der Einrichtung oder dem Betrieb Tierversuche durchgeführt werden.

(2) Der Tierschutzausschuss hat die Aufgabe,

1. die Tierschutzbeauftragten bei der Erfüllung ihrer Aufgaben nach § 5 Absatz 4 Satz 1 Nummer 2 und Satz 2 Nummer 2 zu unterstützen,
2. an der Festlegung interner Arbeitsabläufe, die die Durchführung und Auswertung der Überwachung des Wohlergehens der Tiere sowie diesbezügliche Folgemaßnahmen betreffen, mitzuwirken und die Einhaltung der Arbeitsabläufe zu überprüfen,

3. die Entwicklung von Tierversuchen und deren Ergebnisse unter Berücksichtigung der Auswirkungen auf die verwendeten Tiere zu verfolgen,
4. im Hinblick auf die Entwicklung und Durchführung von Programmen nach § 10 Absatz 2 beratend tätig zu werden,
5. das gesamte mit Tierversuchen sowie mit der Züchtung, Haltung, Pflege und Tötung von Tieren befasste Personal der Einrichtung oder des Betriebes
   a) im Hinblick auf die Erfüllung der Anforderungen des § 7 Absatz 1 Satz 2 und 3 sowie des § 7a Absatz 2 Nummer 2, 4 und 5 des Tierschutzgesetzes und im Hinblick auf Maßnahmen, die zur Verbesserung der Zucht, Unterbringung und Pflege und der bei der Tötung von Tieren angewendeten Verfahren beitragen, zu beraten
   b) laufend über technische und wissenschaftliche Entwicklungen zur Erfüllung der Anforderungen des § 7 Absatz 1 Satz 2 und 3 sowie des § 7a Absatz 2 Nummer 2, 4 und 5 des Tierschutzgesetzes und zur Verbesserung der Zucht, Unterbringung und Pflege und der zur Tötung von Tieren angewendeten Verfahren zu informieren, insbesondere über Entwicklungen zu Möglichkeiten der Verbesserung des Wohlergehens der Tiere,
6. die Entwicklungen und die Ergebnisse von Tierversuchen unter Berücksichtigung der Auswirkungen auf die verwendeten Tiere zu verfolgen sowie
7. Faktoren, auch aufgrund der Erkenntnisse aus den innerbetrieblichen Versuchen, zu ermitteln, die zu einer weitergehenden Erfüllung der Anforderungen des § 7 Absatz 1 Satz 2 und 3 sowie des § 7a Absatz 2 Nummer 2, 4 und 5 des Tierschutzgesetzes und zur Verbesserung der Zucht, Unterbringung und Pflege und der bei der Tötung von Tieren angewendeten Verfahren beitragen, und entsprechende Empfehlungen zu geben, insbesondere zur Verbesserung des Wohlergehens der Tiere.

(3) Der Tierschutzbeauftragte kann Eingaben beim Tierschutzausschuss einreichen.

(4) Der Träger der Einrichtung oder der für den Betrieb Verantwortliche hat sicherzustellen, dass über Empfehlungen des Tierschutzausschusses, die dieser im Rahmen der Erfüllung seiner in Absatz 2 Satz 1 genannten Aufgaben abgibt, sowie über alle Entscheidungen, die im Hinblick auf diese Empfehlungen getroffen werden, Aufzeichnungen geführt und diese mindestens drei Jahre lang aufbewahrt werden. Die Aufzeichnungen sind der zuständigen Behörde auf Verlangen vorzulegen.

§ 7 Führen von Aufzeichnungen

(1) Wer zum Führen von Aufzeichnungen nach § 11a Absatz 1 Satz 1 Nummer 1 des Tierschutzgesetzes verpflichtet ist, hat in den Betriebs- oder Geschäftsräumen ein Kontrollbuch nach Maßgabe der Sätze 2 und 3 zu führen. In das Kontrollbuch nach Satz 1 ist jede Bestandsveränderung mit folgenden Angaben dauerhaft einzutragen:

1. Anzahl und Art der gezüchteten, erworbenen, an Dritte abgegebenen, in Tierversuchen verwendeten und nach § 10 untergebrachten oder verbrachten Tiere,
2. Herkunft der Tiere, einschließlich der Angabe, ob sie zur Verwendung in Tierversuchen gezüchtet worden sind,
3. Zeitpunkt, zu dem die Tiere erworben, geliefert oder nach § 10 untergebracht oder verbracht worden sind,
4. Name und Anschrift der Person, von der die Tiere erworben wurden,
5. Name und Anschrift des Empfängers der Tiere,
6. Anzahl und Art der in einer Einrichtung oder einem Betrieb getöteten oder aus anderen Gründen gestorbenen Tiere sowie im letzteren Falle die Todesursache, soweit bekannt,
7. Auffälligkeiten in Bezug auf den Gesundheitszustand der Tiere.

Hunde, Katzen und Primaten sind einzeln mit folgenden zusätzlichen Angaben aufzuführen:

1. Identität des Tieres,
2. Geburtsort und -datum, soweit bekannt,
3. bei Primaten, ob es sich um einen Nachkommen von in Gefangenschaft gezüchteten Primaten handelt.

Die §§ 239 und 261 des Handelsgesetzbuchs gelten sinngemäß.

(2) Die in Absatz 1 genannten Aufzeichnungen sind, gerechnet ab dem Beginn des Jahres, das auf die Entstehung der Aufzeichnung folgt, mindestens fünf Jahre lang aufzubewahren und der zuständigen Behörde auf Verlangen vorzulegen.

§ 8 Besondere Aufzeichnungen bei Hunden, Katzen und Primaten

(1) Unbeschadet des § 7 hat der zum Führen von Aufzeichnungen nach § 11a Absatz 1 Satz 1 Nummer 1 des Tierschutzgesetzes Verpflichtete bei Hunden, Katzen und Primaten jeweils gesonderte Aufzeichnungen nach Maßgabe des Satzes 2 und des Absatzes 2 Nummer 1 zu führen. Die Aufzeichnungen umfassen bezogen auf das jeweilige Tier alle wesentlichen fortpflanzungsbezogenen, tiermedizinischen und das Verhalten des Tieres betreffenden Informationen sowie Angaben zu den Versuchsvorhaben, in denen es verwendet worden ist.

(2) Der zum Führen der Aufzeichnungen nach Absatz 1 Verpflichtete hat

1. mit dem Führen der Aufzeichnungen unverzüglich nach der Geburt des Tieres zu beginnen,
2. im Falle der Abgabe des Tieres an einen Dritten in anderen als den in § 10 genannten Fällen dem Dritten die jeweiligen Aufzeichnungen vollständig und unverzüglich zu übergeben,
3. im Falle einer Unterbringung des Tieres nach § 10 dem neuen Halter die in den Aufzeichnungen enthaltenen und für die Unterbringung und tiermedizinische Versorgung wesentlichen tiermedizinischen und das Verhalten des Tieres betreffenden Informationen nach Absatz 1 Satz 2 zur Verfügung zu stellen und
4. die Aufzeichnungen, soweit sie nicht nach Nummer 2 weitergegeben wurden, nach der Unterbringung oder dem Verbringen des Tieres nach § 10 oder andernfalls nach dem Tod des Tieres drei Jahre lang aufzubewahren und der zuständigen Behörde auf Verlangen vorzulegen.

§ 9 Kennzeichnung von Hunden, Katzen und Primaten

(1) Wer Hunde, Katzen oder Primaten, die zur Verwendung in Tierversuchen bestimmt sind oder deren Gewebe oder Organe dazu bestimmt sind, zu wissenschaftlichen Zwecken verwendet zu werden, züchtet, hat das jeweilige Tier spätestens zum Zeitpunkt des Absetzens unter Verwendung derjenigen Methode, die für den Versuchszweck geeignet ist und die bei dem jeweiligen Tier die geringsten Schmerzen, Leiden und Schäden verursacht, dauerhaft so zu kennzeichnen, dass seine Identität festgestellt werden kann.

(2) Wer nicht gekennzeichnete Hunde, Katzen oder Primaten zur Abgabe oder Verwendung zu den in Absatz 1 Satz 1 genannten Zwecken erwirbt, hat die Kennzeichnung nach Absatz 1 unverzüglich vorzunehmen und auf Verlangen der zuständigen Behörde den Nachweis zu erbringen, dass es sich um für solche Zwecke gezüchtete Tiere handelt.

(3) Wer nach Absatz 1 oder Absatz 2 Tiere zu kennzeichnen hat, hat ein Verzeichnis der gekennzeichneten Tiere nach Art, Datum und Kennzeichnung zu führen und dies der zuständigen Behörde auf Verlangen vorzulegen.

§ 10 Anderweitige Unterbringung oder Freilassung von Wirbeltieren und Kopffüßern

(1) Wirbeltiere oder Kopffüßer, die in Tierversuchen verwendet worden sind oder die dazu bestimmt gewesen sind, in Tierversuchen verwendet zu werden, deren Verwendung jedoch nicht mehr vorgesehen ist, können dauerhaft außerhalb einer Einrichtung oder eines Betriebs im Sinne des § 1 Absatz 1 untergebracht, in ein für die jeweilige Tierart geeignetes Haltungssystem oder, im Falle von aus der Natur entnommenen Tieren, einen geeigneten Lebensraum verbracht werden, wenn

1. der Gesundheitszustand der Tiere dies zulässt,
2. von den Tieren keine Gefahren für die Gesundheit von Menschen oder anderen Tieren oder für die Umwelt ausgehen und
3. geeignete Maßnahmen ergriffen worden sind, um das Wohlergehen der Tiere sicherzustellen.

(2) Wer nach Absatz 1 Tiere unterbringt, muss über ein Programm für eine solche Unterbringung verfügen, in dessen Rahmen die Gewöhnung der unterzubringenden Tiere gewährleistet wird. Soweit dies aus Gründen des Tierschutzes erforderlich ist, dürfen aus der Natur entnommene Tiere nur im Rahmen eines Auswilderungsprogramms in einen geeigneten Lebensraum verbracht werden.

Unterabschnitt 2

Erlaubnis nach § 11 Absatz 1 Satz 1 Nummer 1 des Tierschutzgesetzes

§ 11 Erlaubnisvoraussetzungen

(1) Die Erlaubnis nach § 11 Absatz 1 Satz 1 Nummer 1 des Tierschutzgesetzes darf nur erteilt werden, wenn

1. die für die Tätigkeit verantwortliche Person auf Grund ihrer Ausbildung oder ihres bisherigen beruflichen oder sonstigen Umgangs mit Tieren die für die Tätigkeit erforderlichen fachlichen Kenntnisse und Fähigkeiten hat,
2. die für die Tätigkeit verantwortliche Person die erforderliche Zuverlässigkeit hat,
3. in den der Tätigkeit dienenden Einrichtungen und Betrieben
   a) geeignete Räumlichkeiten und Anlagen vorhanden sind und
   b) ausreichend sachkundiges Personal zur Verfügung steht,

   sodass eine den Anforderungen des § 2 des Tierschutzgesetzes und des § 1 Absatz 1 entsprechende Haltung der Tiere ermöglicht wird,
4. sichergestellt ist, dass die Personen nach § 3 Absatz 1 Satz 1 Nummer 1 und 2 jederzeit den Nachweis erbringen können, dass sie über die dort genannten Kenntnisse und Fähigkeiten verfügen,
5. die Einhaltung der §§ 4 bis 9 erwartet werden kann und
6. im Fall der Züchtung von Primaten der Züchter über ein Konzept verfügt, mit dessen Hilfe er den Anteil derjenigen Tiere erhöhen kann, die Nachkommen von in Gefangenschaft gezüchteten Primaten sind.

Im Falle des Satzes 1 Nummer 1 sind die erforderlichen fachlichen Kenntnisse und Fähigkeiten auf Verlangen in einem Fachgespräch bei der zuständigen Behörde nachzuweisen.

(2) Die Erlaubnis kann, soweit es zum Schutz der Tiere erforderlich ist, unter Befristungen, Bedingungen und Auflagen erteilt werden.

§ 12 Beantragen der Erlaubnis

In dem Antrag auf Erteilung der Erlaubnis nach § 11 Absatz 1 Satz 1 Nummer 1 des Tierschutzgesetzes sind anzugeben

1. Name und Anschrift des Antragstellers,
2. die der Tätigkeit dienenden Einrichtungen und Betriebe einschließlich der dort vorhandenen Räumlichkeiten und Anlagen und des dort vorhandenen Personals,
3. die Art der betroffenen Tiere sowie, tierartbezogen, die Haltungskapazitäten,
4. der Name der für die Tätigkeit verantwortlichen Person,
5. das Vorhandensein von Personen nach § 3 Absatz 1 Satz 1 Nummer 1 und 2 und § 4 und
6. der Name der Tierschutzbeauftragten nach § 5.

Dem Antrag sind Nachweise über die Kenntnisse und Fähigkeiten nach § 11 Absatz 1 Satz 1 Nummer 1 beizufügen.

§ 13 Erlaubnisbescheid, Anzeige und Erlaubnis von Änderungen

(1) In dem Erlaubnisbescheid sind die Personen nach § 12 Satz 1 Nummer 4 und 6 anzugeben.

(2) Wechselt eine der in Absatz 1 genannten Personen, so hat der Inhaber der Erlaubnis diese Änderung der zuständigen Behörde unverzüglich anzuzeigen. Die Erlaubnis ist innerhalb eines Monats ab Eingang der Änderungsanzeige zu widerrufen, wenn auf Grund der angezeigten Änderungen die Voraussetzungen für die Erteilung der Erlaubnis nicht mehr vorliegen. Satz 1 gilt entsprechend für eine Änderung der nach § 12 Satz 1 Nummer 2 und 3 angegebenen Sachverhalte, es sei denn es ist ausgeschlossen, dass sich diese Änderung nachteilig auf das Wohlergehen der Tiere auswirkt.

(3) Jede erhebliche Änderung der in § 12 Satz 1 Nummer 2 und 3 genannten Sachverhalte, die sich nachteilig auf das Wohlergehen der Tiere auswirken könnte, bedarf einer erneuten Erlaubnis.

Abschnitt 2

Durchführung, Genehmigung und Anzeige von Tierversuchen

§ 14 Geltung für Tiere in einem frühen Entwicklungsstadium

Die §§ 7 bis 9 des Tierschutzgesetzes sowie die §§ 15 bis 43 gelten auch für die Durchführung von Tierversuchen, einschließlich der Genehmigung und Anzeige von Versuchsvorhaben,

1. in denen
   a) Larven von Wirbeltieren, soweit diese in der Lage sind, selbstständig Nahrung aufzunehmen, oder
   b) Föten von Säugetieren ab dem letzten Drittel ihrer normalen Entwicklung vor der Geburt

   verwendet werden oder verwendet werden sollen oder
2. in denen andere als in Nummer 1 genannte Wirbeltiere in einem Entwicklungsstadium vor der Geburt oder dem Schlupf verwendet werden oder verwendet werden sollen, wenn die Tiere über dieses Entwicklungsstadium hinaus weiterleben sollen und nach der Geburt oder dem Schlupf infolge der Verwendung voraussichtlich Schmerzen oder Leiden empfinden oder Schäden erleiden werden.

Unterabschnitt 1
Durchführung von Tierversuchen

§ 15 Anforderungen an Räumlichkeiten und Anlagen

(1) Tierversuche an Wirbeltieren und Kopffüßern dürfen nur in den Räumlichkeiten einer Einrichtung oder eines Betriebs im Sinne des § 1 Absatz 1 durchgeführt werden. § 2 Absatz 1 Satz 2 gilt entsprechend. Abweichend von Satz 1 darf ein Tierversuch außerhalb einer Einrichtung oder eines Betriebs durchgeführt werden, wenn wissenschaftlich begründet dargelegt ist, dass dies im Hinblick auf den Zweck des Versuchs erforderlich ist.

(2) Unbeschadet des § 1 Absatz 1 Nummer 1 müssen die in der Einrichtung oder dem Betrieb nach Absatz 1 Satz 1 für die Durchführung von Tierversuchen an Wirbeltieren und Kopffüßern bestimmten Räumlichkeiten, Anlagen und Gegenstände

1. hierfür geeignet sein und den Anforderungen des Anhangs III Teil A Nummer 1.3 der Richtlinie 2010/63/EU entsprechen und
2. durch ihre Gestaltung, Konstruktion und Funktionsweise gewährleisten, dass die Tierversuche zielgerichtet durchgeführt werden, um unter Verwendung der geringstmöglichen Anzahl an Tieren sowie unter Verursachung der geringstmöglichen Schmerzen, Leiden und Schäden zuverlässige Ergebnisse zu erzielen.

§ 16 Anforderungen an die Sachkunde

(1) Tierversuche an Wirbeltieren und Kopffüßern dürfen nur von Personen durchgeführt werden, die über die Kenntnisse und Fähigkeiten nach Anlage 1 Abschnitt 3 verfügen. Darüber hinaus dürfen Tierversuche nur

1. von Personen mit abgeschlossenem Hochschulstudium der Veterinärmedizin, der Medizin oder der Zahnmedizin,
2. von Personen mit abgeschlossenem naturwissenschaftlichen Hochschulstudium, sofern sie nachweislich die erforderlichen Kenntnisse und Fähigkeiten haben, oder
3. von Personen, die nachweislich im Rahmen einer abgeschlossenen Berufsausbildung die erforderlichen Kenntnisse und Fähigkeiten erworben haben,

durchgeführt werden. Tierversuche mit operativen Eingriffen an Wirbeltieren dürfen unbeschadet des Satzes 1 nur

1. von Personen mit abgeschlossenem Hochschulstudium der Veterinärmedizin, der Medizin oder der Zahnmedizin oder
2. von Personen mit abgeschlossenem naturwissenschaftlichen Hochschulstudium oder einer Weiterbildung im Anschluss an ein naturwissenschaftliches Hochschulstudium, sofern sie nachweislich die erforderlichen Kenntnisse und Fähigkeiten haben,

durchgeführt werden. Die Sätze 2 und 3 gelten nicht für Tierversuche nach § 7 Absatz 2 Satz 2 Nummer 1 des Tierschutzgesetzes, die nach bereits erprobten Verfahren vorgenommen werden. Die zuständige Behörde genehmigt Ausnahmen von den Sätzen 2 und 3, wenn der Nachweis der erforderlichen Kenntnisse und Fähigkeiten auf andere Weise erbracht ist.

(2) Tierversuche, die Aus-, Fort- oder Weiterbildungszwecken dienen, dürfen abweichend von § 7 Absatz 1 Satz 4 des Tierschutzgesetzes und Absatz 1 Satz 1 bis 3 auch von Personen, die die dort genannten Anforderungen nicht erfüllen, durchgeführt werden, soweit dies in Anwesenheit und unter Aufsicht einer Person erfolgt, die die jeweiligen Anforderungen erfüllt.

(3) Tierversuche dürfen nur durchgeführt werden, wenn die Personen, von denen das Versuchsvorhaben und die beabsichtigten Tierversuche geplant worden sind, über die hierfür erforderlichen Kenntnisse und Fähigkeiten einschließlich der Kenntnisse und Fähigkeiten nach Anlage 1 Abschnitt 3 verfügen und diese der zuständigen Behörde auf Verlangen nachweisen.

§ 17 Schmerzlinderung und Betäubung

(1) Bei der Durchführung von Versuchen an Wirbeltieren und Kopffüßern ist durch Anwendung schmerzlindernder Mittel oder Verfahren sicherzustellen, dass Schmerzen und Leiden bei dem verwendeten Tier auf das geringstmögliche Maß vermindert werden.

(2) Versuche an Wirbeltieren oder Kopffüßern dürfen nur unter Narkose oder lokaler Schmerzausschaltung (Betäubung) durchgeführt werden. Satz 1 gilt nicht, wenn

1. die für das jeweilige Tier mit der Durchführung des Versuchs verbundenen Schmerzen geringfügiger als die mit einer Betäubung verbundenen Schmerzen und Leiden sind oder
2. der Zweck des Versuchs eine Betäubung ausschließt und der Versuch bei dem jeweiligen Tier nicht zu schweren Verletzungen führt.

Die Betäubung darf bei Wirbeltieren nur von einer Person, die die Voraussetzungen des § 7 Absatz 1 Satz 4 des Tierschutzgesetzes und des § 16 Absatz 1 Satz 2 erfüllt, oder, soweit die Durchführung der Betäubung Ausbildungs-, Fortbildungs- oder Weiterbildungszwecken dient, in Anwesenheit und unter Aufsicht einer solchen Person vorgenommen werden. § 16 Absatz 1 Satz 5 gilt entsprechend.

(3) Ist bei einem betäubten Wirbeltier oder Kopffüßer damit zu rechnen, dass mit Abklingen der Betäubung Schmerzen auftreten, so muss das Tier rechtzeitig mit schmerzlindernden Mitteln oder Verfahren behandelt werden. Dies gilt, soweit ethisch vertretbar, nicht, wenn wissenschaftlich begründet dargelegt wird, dass die Behandlung mit schmerzlindernden Mitteln oder Verfahren mit dem Zweck des Tierversuchs unvereinbar ist.

(4) Bei einem betäubten Wirbeltier oder Kopffüßer dürfen Mittel, durch die das Äußern von Schmerzen verhindert oder beeinträchtigt wird, nur angewendet werden, wenn wissenschaftlich begründet worden ist:

1. die Notwendigkeit der Anwendung der Mittel, durch die das Äußern von Schmerzen verhindert oder beeinträchtigt wird,
2. die angemessene Anwendung der Mittel zur Narkose oder lokalen Schmerzausschaltung und
3. in den Fällen des Absatzes 3 Satz 1 die angemessene Anwendung der schmerzlindernden Mittel.

In der Begründung nach Satz 1 ist das anzuwendende Mittel anzugeben und zur erläutern, dass der Einsatz von dem Mittel nicht dazu dient, den Ausdruck von Schmerz zu verhindern oder zu beschränken, weil das Tier aufgrund der gleichzeitigen Gabe des Betäubungsmittels oder der Analgetika hinreichend davor geschützt ist, tatsächlich Schmerz wahrzunehmen.

(5) Bei einem nicht betäubten Wirbeltier oder Kopffüßer dürfen keine Mittel angewendet werden, durch die das Äußern von Schmerzen verhindert oder beeinträchtigt wird.

§ 18 Erneutes Verwenden von Wirbeltieren und Kopffüßern

(1) Ein Wirbeltier oder ein Kopffüßer, das oder der bereits in einem Versuchsvorhaben verwendet worden ist, darf in einem weiteren Versuchsvorhaben, für das auch ein zuvor noch nicht verwendetes Tier verwendet werden könnte, nur dann verwendet werden, wenn

1. das Tier nicht in einem Tierversuch verwendet worden ist, der nach Artikel 15 Absatz 1 in Verbindung mit Anhang VIII der Richtlinie 2010/63/EU als „schwer“ einzustufen ist,
2. sein allgemeiner Gesundheitszustand und sein Wohlbefinden vollständig wiederhergestellt sind,

3. das Tier im Rahmen des weiteren Versuchsvorhabens nicht in einem Tierversuch verwendet wird, der nach Artikel 15 Absatz 1 in Verbindung mit Anhang VIII der Richtlinie 2010/63/EU als „schwer“ einzustufen ist und
4. die erneute Verwendung im Einklang mit einer tierärztlichen Empfehlung steht, die Art und Umfang der Schmerzen, Leiden und Schäden berücksichtigt, die das jeweilige Tier während seines gesamten bisherigen Lebensverlaufs erfahren hat.

(2) Die zuständige Behörde kann abweichend von Absatz 1 Nummer 1 die Verwendung eines Wirbeltieres oder eines Kopffüßers in einem weiteren Versuchsvorhaben genehmigen, wenn das Tier

1. nicht mehr als einmal in einem Tierversuch verwendet worden ist, der nach Artikel 15 Absatz 1 in Verbindung mit Anhang VIII der Richtlinie 2010/63/EU als „schwer“ einzustufen ist,
2. im Rahmen des weiteren Versuchsvorhabens nicht in einem Tierversuch verwendet wird, der nach Artikel 15 Absatz 1 in Verbindung mit Anhang VIII der Richtlinie 2010/63/EU als „schwer“ oder „mittel“ einzustufen ist, und
3. zuvor einer tierärztlichen Untersuchung unterzogen worden ist.

§ 19 Verwenden gezüchteter Wirbeltiere und Kopffüßer

(1) Wirbeltiere und Kopffüßer dürfen in Tierversuchen nur verwendet werden, wenn sie für einen solchen Zweck gezüchtet worden sind. Die zuständige Behörde kann, soweit es mit dem Schutz der Tiere vereinbar ist, Ausnahmen hiervon genehmigen, wenn wissenschaftlich begründet dargelegt ist, dass die Verwendung von anderen als nach Satz 1 gezüchteten Tieren erforderlich ist.

(2) Absatz 1 Satz 1 gilt nicht für die Verwendung von Pferden, Rindern, Schweinen, Schafen, Ziegen, Hühnern, Tauben, Puten, Enten, Gänsen oder Fischen, ausgenommen Zebrabärblinge.

§ 20 Verwenden wildlebender Tiere

(1) Aus der Natur entnommene Tiere dürfen in Tierversuchen nicht verwendet werden. Die zuständige Behörde kann Ausnahmen hiervon genehmigen, wenn der Zweck des Versuchs nicht durch die Verwendung anderer Tiere erreicht werden kann.

(2) Wirbeltiere oder Kopffüßer, die aus der Natur entnommen werden sollen, dürfen nur von Personen gefangen werden, die die dafür erforderlichen Kenntnisse und Fähigkeiten haben. Schmerzen, Leiden oder Schäden dürfen den Tieren dabei nur in dem Maße zugefügt werden, als dies für den Fang unerlässlich ist.

(3) Wird bei oder nach dem Einfangen nach Absatz 2 festgestellt, dass das Tier verletzt ist oder sich in einem schlechten gesundheitlichen Zustand befindet, so ist es einem Tierarzt oder einer anderen sachkundigen Person vorzustellen und es sind Maßnahmen zu ergreifen, um Schmerzen, Leiden und Schäden des Tieres auf das mit dem Zweck des Tierversuchs vereinbare, geringstmögliche Maß zu vermindern.

§ 21 Verwenden herrenloser oder verwilderter Haustiere

Herrenlose oder verwilderte Tiere von Tierarten, die üblicherweise in menschlicher Obhut gehalten werden, dürfen in Tierversuchen nicht verwendet werden. Die zuständige Behörde kann Ausnahmen hiervon genehmigen, wenn

1. der Tierversuch zur Deckung eines grundlegenden Bedarfs an Studien über die Gesundheit und das Wohlergehen dieser Tiere oder über gewichtige Gefahren für die Umwelt oder die Gesundheit von Menschen oder Tieren durchgeführt wird und
2. wissenschaftlich begründet dargelegt ist, dass der Zweck des Tierversuchs nur durch die Verwendung eines Tieres nach Satz 1 erreicht werden kann.

§ 22 Verwenden geschützter Tierarten

In Anhang A der Verordnung (EG) Nr. 338/97 des Rates vom 9. Dezember 1996 über den Schutz von Exemplaren wildlebender Tier- und Pflanzenarten durch Überwachung des Handels (ABl. L 61 vom 3.3.1997, S. 1) in der jeweils geltenden Fassung aufgeführte Wirbeltiere, die nicht Primaten sind, und Kopffüßer dürfen in Tierversuchen nicht verwendet werden. Satz 1 gilt nicht, wenn

1. der Tierversuch

a) dem Zweck des Vorbeugens, Erkennens oder Behandelns von Krankheiten, Leiden, Körperschäden oder körperlichen Beschwerden bei Menschen oder Tieren oder der Entwicklung und Herstellung sowie Prüfung der Qualität, Wirksamkeit oder Unbedenklichkeit von Stoffen oder Produkten im Hinblick auf die in § 7a Absatz 1 Satz 1 Nummer 2 des Tierschutzgesetzes genannten Zwecke oder

b) der Forschung im Hinblick auf die Erhaltung der Arten

dient und

2. wissenschaftlich begründet dargelegt ist, dass der in Nummer 1 genannte Zweck des Tierversuchs nicht durch die Verwendung anderer als der in Anhang A der Verordnung (EG) Nr. 338/97 genannten Tierarten erreicht werden kann.

Satz 1 gilt nicht für in Gefangenschaft geborene und gezüchtete Tiere oder künstlich vermehrte Tiere nach Artikel 7 Absatz 1 der Verordnung (EG) Nr. 338/97. Satz 2 gilt nicht für Tierversuche, die der Grundlagenforschung dienen.

§ 23 Verwenden von Primaten

(1) Primaten dürfen in Tierversuchen nicht verwendet werden.

(2) Absatz 1 gilt, vorbehaltlich der Absätze 4 und 5, nicht, wenn

1. der Tierversuch

   a) der Grundlagenforschung,

   b) dem Zweck des Vorbeugens, Erkennens oder Behandelns von Krankheiten, Leiden, Körperschäden oder körperlichen Beschwerden bei Menschen, die lebensbedrohlich sein können oder zu einer Verminderung der körperlichen oder geistigen Funktionsfähigkeit führen, oder der Entwicklung und Herstellung sowie Prüfung der Qualität, Wirksamkeit oder Unbedenklichkeit von Stoffen oder Produkten hinsichtlich der genannten Beeinträchtigungen der menschlichen Gesundheit oder

   c) der Forschung im Hinblick auf die Erhaltung der Arten

   dient und

2. wissenschaftlich begründet dargelegt ist, dass der in Nummer 1 genannte Zweck des Tierversuchs nicht durch die Verwendung anderer Tierarten als Primaten erreicht werden kann.

(3) Abweichend von Absatz 2 Nummer 1 kann die zuständige Behörde die Verwendung von Primaten in einem Tierversuch auch dann genehmigen, wenn der Tierversuch der Forschung mit dem Zweck des Vorbeugens, Erkennens oder Behandelns anderer als der in Absatz 2 Nummer 1 Buchstabe b genannten Krankheiten, Leiden, Körperschäden oder körperlichen Beschwerden bei Menschen dient, soweit wissenschaftlich begründet dargelegt ist, dass die Verwendung von Primaten zur Erreichung des genannten Zwecks des Tierversuchs unerlässlich ist.

(4) Im Falle von Primaten, die in Anhang A der Verordnung (EG) Nr. 338/97 aufgeführt sind und nicht unter Artikel 7 Absatz 1 der Verordnung (EG) Nr. 338/97 fallen, gilt Absatz 1 nicht, wenn

1. der Tierversuch

   a) dem Zweck des Vorbeugens, Erkennens oder Behandelns von Krankheiten, Leiden, Körperschäden oder körperlichen Beschwerden bei Menschen, die lebensbedrohlich sein können oder zu einer Verminderung der körperlichen oder geistigen Funktionsfähigkeit führen, oder der Entwicklung und Herstellung sowie Prüfung der Qualität, Wirksamkeit oder Unbedenklichkeit von Stoffen oder Produkten hinsichtlich der genannten Beeinträchtigungen der menschlichen Gesundheit oder

   b) der Forschung im Hinblick auf die Erhaltung der Arten

   dient und

2. wissenschaftlich begründet dargelegt ist, dass der in Nummer 1 genannte Zweck des Tierversuchs nicht durch die Verwendung anderer Tierarten als der in Absatz 1 genannten und in Anhang A der Verordnung (EG) Nr. 338/97 genannten und nicht unter Artikel 7 Absatz 1 der Verordnung (EG) Nr. 338/97 fallenden Primaten erreicht werden kann.

Satz 1 gilt nicht für Tierversuche, die der Grundlagenforschung dienen.

(5) Abweichend von Absatz 1 kann die zuständige Behörde die Verwendung von Menschenaffen in einem Tierversuch genehmigen, wenn

1. der Tierversuch
   a) dem Zweck des Vorbeugens, Erkennens oder Behandelns von Krankheiten, Leiden, Körperschäden oder körperlichen Beschwerden bei Menschen, die lebensbedrohlich sind oder zu einer Verminderung der körperlichen oder geistigen Funktionsfähigkeit führen und die unerwartet aufgetreten sind, oder der Entwicklung und Herstellung sowie Prüfung der Qualität, Wirksamkeit oder Unbedenklichkeit von Stoffen oder Produkten hinsichtlich der genannten Beeinträchtigungen der menschlichen Gesundheit oder
   b) der Forschung im Hinblick auf die Erhaltung der Arten

   dient und
2. wissenschaftlich begründet dargelegt ist, dass
   a) Grund zu der Annahme besteht, dass die Durchführung des Tierversuchs zur Erreichung des in Nummer 1 genannten Zwecks des Tierversuchs unerlässlich ist und
   b) dieser Zweck nicht durch die Verwendung anderer Tierarten als Menschenaffen erreicht werden kann.

Satz 1 gilt nicht für Tierversuche, die der Grundlagenforschung dienen.

§ 24 Herkunft zu verwendender Primaten

(1) Primaten, die in Anhang II Spalte 1 der Richtlinie 2010/63/EU aufgeführt sind, dürfen ab dem in Anhang II Spalte 2 der Richtlinie 2010/63/EU jeweils genannten Zeitpunkt nur in Tierversuchen verwendet werden, wenn sie Nachkommen von in Gefangenschaft gezüchteten Primaten sind oder wenn sie aus sich selbst erhaltenden Kolonien im Sinne des Artikels 10 Absatz 1 Unterabsatz 3 der Richtlinie 2010/63/EU stammen.

(2) Abweichend von Absatz 1 kann die zuständige Behörde die Verwendung von in Anhang II Spalte 1 der Richtlinie 2010/63/EU aufgeführten Primaten anderer Abstammung oder Herkunft genehmigen, wenn wissenschaftlich begründet dargelegt ist, dass die Verwendung dieser Primaten erforderlich ist.

§ 25 Durchführung besonders belastender Tierversuche

(1) Tierversuche an Wirbeltieren oder Kopffüßern, die bei den verwendeten Tieren zu voraussichtlich länger anhaltenden oder sich wiederholenden erheblichen Schmerzen oder Leiden führen, dürfen nur durchgeführt werden, wenn die angestrebten Ergebnisse vermuten lassen, dass sie für wesentliche Bedürfnisse von Mensch oder Tier einschließlich der Lösung wissenschaftlicher Probleme von hervorragender Bedeutung sein werden.

(2) Tierversuche nach Absatz 1 dürfen nicht durchgeführt werden, wenn die erheblichen Schmerzen oder Leiden länger anhalten und nicht gelindert werden können. Abweichend von Satz 1 kann die zuständige Behörde die Durchführung eines Tierversuchs nach Satz 1 genehmigen, soweit die Voraussetzungen des Absatzes 1 vorliegen und wissenschaftlich begründet dargelegt ist, dass die Durchführung des Tierversuchs wegen der Bedeutung der angestrebten Erkenntnisse unerlässlich ist.

§ 26 Genehmigungen in besonderen Fällen

(1) Eine Genehmigung nach § 23 Absatz 3 oder 5 oder § 25 Absatz 2 Satz 2 wird von der zuständigen Behörde unter dem Vorbehalt erteilt, dass die Genehmigung im Falle einer Entscheidung der Europäischen Kommission nach Artikel 55 Absatz 4 Unterabsatz 2 Buchstabe b der Richtlinie 2010/63/EU widerrufen wird.

(2) Erteilt die zuständige Behörde eine Genehmigung nach Absatz 1, so hat sie dies dem Bundesministerium für Landwirtschaft, Ernährung und Heimat (Bundesministerium) unverzüglich mitzuteilen. Die Mitteilung nach Satz 1 enthält eine ausführliche Begründung für die Entscheidung der zuständigen Behörde im Hinblick auf das Vorliegen der jeweiligen Genehmigungsvoraussetzungen. Das Bundesministerium unterrichtet nach Eingang der Mitteilung nach Satz 1 die Europäische Kommission nach Artikel 55 Absatz 4 Unterabsatz 1 der Richtlinie 2010/63/EU auf der Grundlage der Mitteilung der zuständigen Behörde nach Satz 2.

§ 27 Zweckerreichung

(1) Sobald der Zweck eines Tierversuchs erreicht ist, sind Maßnahmen zu ergreifen, um die Schmerzen, Leiden und Schäden der verwendeten Tiere auf das geringstmögliche Maß zu vermindern.

(2) Tierversuche sind so zu planen und durchzuführen, dass der Zweck des Versuchs erreicht werden kann, ohne dass die verwendeten Tiere unmittelbar unter der Versuchseinwirkung sterben. Dabei ist insbesondere sicherzustellen, dass der infolge der Versuchseinwirkung bevorstehende Tod eines Tieres so früh wie möglich erkannt und das Tier in diesem Fall unverzüglich unter größtmöglicher Vermeidung von Schmerzen und Leiden getötet wird. Die Sätze 1 und 2 gelten nicht, soweit der Tod der verwendeten Tiere unmittelbar unter der Versuchseinwirkung zur Erreichung des Zwecks des Tierversuchs unerlässlich ist; in diesem Fall ist der Versuch so durchzuführen, dass

1. möglichst wenige der verwendeten Tiere sterben und
2. die Dauer und die Intensität der Schmerzen und Leiden der Tiere auf das geringstmögliche Maß vermindert und der Tod unter größtmöglicher Vermeidung von Schmerzen und Leiden gewährleistet wird.

§ 28 Verfahren nach Abschluss, Nachbehandlung

(1) Nach Abschluss eines Tierversuchs entscheidet ein Tierarzt oder eine andere sachkundige Person darüber, ob ein verwendetes Wirbeltier oder ein verwendeter Kopffüßer, dessen weitere Verwendung in dem jeweiligen Versuchsvorhaben nicht mehr vorgesehen ist, am Leben bleiben oder, wenn ein vernünftiger Grund dafür vorliegt, getötet werden soll. Sind Primaten, Einhufer, Paarhufer, Hunde, Hamster, Katzen, Kaninchen oder Meerschweinchen verwendet worden, so sind diese unverzüglich einem Tierarzt zur Untersuchung vorzustellen.

(2) Kann nach Abschluss eines Tierversuchs ein verwendetes Wirbeltier oder ein verwendeter Kopffüßer nach dem Urteil des Tierarztes oder der sachkundigen Person nur unter mehr als geringfügigen Schmerzen, Leiden oder Schäden weiterleben, so ist das Tier unverzüglich schmerzlos zu töten.

(3) Andere als die in Absatz 2 genannten Tiere sind schmerzlos zu töten, wenn ein vernünftiger Grund dafür vorliegt und dies nach dem Urteil einer sachkundigen Person erforderlich ist.

(4) Soll ein Tier nach Abschluss eines Tierversuchs am Leben erhalten werden, so muss es seinem Gesundheitszustand entsprechend gepflegt und untergebracht und dabei von einem Tierarzt oder einer anderen sachkundigen Person beobachtet und erforderlichenfalls medizinisch versorgt werden.

§ 29 Führen von Aufzeichnungen zu Tierversuchen

(1) In den nach § 9 Absatz 5 Satz 1 des Tierschutzgesetzes zu führenden Aufzeichnungen sind für jedes Versuchsvorhaben, in dem Wirbeltiere, Kopffüßer oder Zehnfußkrebse verwendet werden, der Zweck sowie die Zahl und die Art der verwendeten Tiere und die Art und Durchführung der Tierversuche sowie die Namen der Personen, die die Tierversuche durchgeführt haben, anzugeben. Werden Wirbeltiere verwendet, so ist auch ihre Herkunft einschließlich des Namens und der Anschrift des Vorbesitzers anzugeben. Bei Hunden, Katzen und Primaten sind zusätzlich das Geschlecht, eine an dem Tier vorgenommene Kennzeichnung nach § 9 und bei Hunden und Katzen die Rasse anzugeben.

(2) Die Aufzeichnungen nach Absatz 1 sind von den Personen, die die Tierversuche durchgeführt haben, und von dem Leiter des Versuchsvorhabens oder seinem Stellvertreter zu unterzeichnen. Werden die Aufzeichnungen elektronisch erstellt, sind sie unverzüglich nach Abschluss jedes Teilversuches des Versuchsvorhabens

1. auszudrucken und von dem Leiter des Versuchsvorhabens oder seinem Stellvertreter zu unterzeichnen oder
2. von dem Leiter des Versuchsvorhabens oder seinem Stellvertreter mit einem Zeitstempel unter Verwendung einer fortgeschrittenen elektronischen Signatur zu versehen, auf einem dauerhaften Datenträger zu speichern und auf Verlangen der zuständigen Behörde auszudrucken.

Die §§ 239 und 261 des Handelsgesetzbuchs gelten sinngemäß. Aufzeichnungen zu einem Versuchsvorhaben sind fünf Jahre lang, beginnend mit dem Abschluss des Tierversuchs, aufzubewahren und der zuständigen Behörde auf Verlangen vorzulegen. Vorbehaltlich anderer gesetzlicher Regelungen sind in diesen Aufzeichnungen enthaltene personenbezogene Daten nach Ablauf der Aufbewahrungsfrist nach Satz 4 unverzüglich, bei elektronischer Speicherung, sofern technisch möglich, automatisiert zu löschen.

§ 30 Pflichten des Leiters

(1) Der Leiter des Versuchsvorhabens oder im Falle dessen Verhinderung sein Stellvertreter hat sicherzustellen, dass die Vorschriften der §§ 15 bis 25 und 27 bis 29 eingehalten werden.

(2) Der Leiter des Versuchsvorhabens oder im Falle dessen Verhinderung sein Stellvertreter hat sicherzustellen, dass sobald bei der Durchführung des Versuchsvorhabens vermeidbare Schmerzen, Leiden oder Schäden bei einem Tier verursacht werden, dies unverzüglich unterbunden wird. Er hat darüber hinaus sicherzustellen, dass das Versuchsvorhaben

1. entsprechend der Genehmigung nach § 8 Absatz 1 Satz 1 des Tierschutzgesetzes und
2. unter Beachtung aller im Hinblick auf das Versuchsvorhaben getroffenen Anordnungen, Auflagen und Bedingungen der zuständigen Behörde

durchgeführt wird. Dabei hat er sicherzustellen, dass im Falle einer diesbezüglichen Abweichung geeignete Abhilfemaßnahmen ergriffen und über die Abweichungen und die ergriffenen Abhilfemaßnahmen Aufzeichnungen geführt werden.

(3) Der Leiter des Versuchsvorhabens oder sein Stellvertreter hat sicherzustellen, dass bei der Planung und Durchführung des Versuchsvorhabens die Möglichkeiten, das Wohlergehen der Tiere zu verbessern, berücksichtigt werden.

Unterabschnitt 2
Genehmigung und Anzeige von Versuchsvorhaben

§ 31 Beantragen der Genehmigung

(1) Der Antrag auf Genehmigung eines Versuchsvorhabens nach § 8 Absatz 1 Satz 1 des Tierschutzgesetzes ist schriftlich oder elektronisch bei der zuständigen Behörde zu stellen. In dem Antrag

1. sind anzugeben
   a) Name und Anschrift des Antragstellers,
   b) eine Beschreibung und wissenschaftliche Rechtfertigung des Versuchsvorhabens einschließlich des damit verfolgten Zweckes,
   c) eine wissenschaftliche Rechtfertigung der Art, der Herkunft, des Lebensabschnittes und der geschätzten Anzahl der für das Versuchsvorhaben vorgesehenen Tiere,
   d) die Art und die Durchführung der beabsichtigten Tierversuche einschließlich des geplanten Einsatzes von Mitteln und Methoden zum Zwecke der Betäubung oder Schmerzlinderung sowie die Sachverhalte, bei deren Vorliegen ein Tier nicht mehr in den Tierversuchen verwendet wird,
   e) der Ort, der Zeitpunkt des Beginns und die voraussichtliche Dauer des Versuchsvorhabens,
   f) der Name, die Anschrift und die Sachkunde des Leiters des Versuchsvorhabens und seines Stellvertreters, der Personen, von denen das Versuchsvorhaben oder die beabsichtigten Tierversuche geplant worden sind, und der durchführenden Personen sowie die für die Nachbehandlung in Frage kommenden Personen,
   g) soweit eine Tötung der Tiere vorgesehen ist, das Verfahren, das hierzu angewandt werden soll,
   h) eine Zusammenfassung der Maßnahmen zur Verminderung, Vermeidung und Linderung jeglicher Form des Leidens von Tieren von ihrer Geburt bis zu ihrem Tod,
   i) Informationen zu den Versuchs- und Beobachtungsstrategien und zur statistischen Gestaltung zur Minimierung der Anzahl der Tiere, der Schmerzen, des Leidens, der Schäden und gegebenenfalls der Auswirkungen auf die Umwelt,
   j) Methoden, mit denen die Erfüllung der Anforderungen des § 7 Absatz 1 Satz 2 und 3 sowie des § 7a Absatz 2 Nummer 2, 4 und 5 des Tierschutzgesetzes an die Verwendung von Tieren in Verfahren sichergestellt wird, sowie
   k) vorgesehene Eingewöhnungs- und Trainingsprogramme, die für die Tiere, die Verfahren und die Dauer des Versuchsvorhabens geeignet sind,
2. ist wissenschaftlich begründet darzulegen,
   a) dass die Voraussetzungen des § 8 Absatz 1 Satz 2 Nummer 1 Buchstabe a und b des Tierschutzgesetzes vorliegen,
   b) in welchen Schweregrad der Versuch eingestuft wird und
   c) im Fall des § 17 Absatz 4 unter Angabe der dort genannten Mittel

aa) die Notwendigkeit der Anwendung der Mittel, durch die das Äußern von Schmerzen verhindert oder beeinträchtigt wird,

bb) die angemessene Anwendung der Mittel zur Narkose oder zur lokalen Schmerzausschaltung und

cc) im Fall des § 17 Absatz 4 Satz 1 Nummer 3 die angemessene Anwendung der schmerzlindernden Mittel,

3. ist nachzuweisen, dass die Voraussetzungen des § 8 Absatz 1 Satz 2 Nummer 2 bis 5 des Tierschutzgesetzes vorliegen, und
4. ist darzulegen,
   a) dass die Voraussetzungen des § 8 Absatz 1 Satz 2 Nummer 6 bis 8 des Tierschutzgesetzes vorliegen und
   b) wie Belange der Umwelt berücksichtigt werden sollen.

(2) Dem Antrag ist eine Zusammenfassung des Versuchsvorhabens mit den Angaben nach § 41 Absatz 1 Satz 2 beizufügen.

(3) Dem Antrag auf Genehmigung eines Versuchsvorhabens können wissenschaftliche Beurteilungen von unabhängigen Dritten beigefügt werden.

§ 32 Genehmigungsverfahren, Bearbeitungsfristen

(1) Die zuständige Behörde hat innerhalb von 40 Arbeitstagen ab dem Eingang eines den Anforderungen des § 31 entsprechenden Antrags dem Antragsteller ihre Entscheidung über den Antrag mitzuteilen. Soweit der Umfang und die Schwierigkeit der Prüfung des Vorliegens der Voraussetzungen nach § 8 Absatz 1 Satz 2 des Tierschutzgesetzes dies rechtfertigen, kann die zuständige Behörde den in Satz 1 genannten Zeitraum einmalig um bis zu 15 Arbeitstage nach Maßgabe des Absatzes 2 Satz 3 verlängern.

(2) Nach Eingang eines Antrags nach § 31 Absatz 1 Satz 1 hat die zuständige Behörde dem Antragsteller unverzüglich eine Empfangsbestätigung auszustellen. In der Empfangsbestätigung ist anzugeben, dass dem Antragsteller die Entscheidung über den Antrag innerhalb des in Absatz 1 Satz 1 genannten Zeitraums mitgeteilt wird. Eine Verlängerung nach Absatz 1 Satz 2 ist dem Antragsteller spätestens bis zum Ablauf des in Absatz 1 Satz 1 genannten Zeitraums unter Angabe von Gründen mitzuteilen.

(3) Die zuständige Behörde überprüft einen eingegangenen Antrag nach § 31 Absatz 1 Satz 1 unverzüglich nach Eingang auf Vollständigkeit. Soweit dieser den Anforderungen des § 31 nicht genügt, teilt die zuständige Behörde dies dem Antragsteller unverzüglich unter Benennung der fehlenden Angaben und Unterlagen mit. Der Antragsteller ist darauf hinzuweisen, dass der Beginn des in Absatz 1 Satz 1 genannten Zeitraums den Eingang eines den Anforderungen des § 31 entsprechenden Antrags voraussetzt.

(4) Die zuständige Behörde unterrichtet unverzüglich die Kommission nach § 15 Absatz 1 Satz 2 des Tierschutzgesetzes über vorliegende Anträge auf Genehmigung von Versuchsvorhaben und gibt ihr Gelegenheit, in angemessener Frist Stellung zu nehmen. Die zuständige Behörde kann der Kommission auch Anzeigen von Änderungen genehmigter Versuchsvorhaben zur Stellungnahme vorlegen, soweit der Umfang und die Schwierigkeit der Prüfung dies erfordern.

(4a) Die zuständige Behörde berücksichtigt bei ihrer Entscheidung über das Vorliegen der Voraussetzungen nach § 8 Absatz 1 Satz 2 Nummer 1 Buchstabe a und b des Tierschutzgesetzes die wissenschaftlich begründeten Darlegungen des Antragstellers nach § 31 Absatz 1 Satz 2 Nummer 2 sowie die wissenschaftlichen Beurteilungen nach § 31 Absatz 3.

(5) Absatz 4 gilt für die zuständige Stelle der Bundeswehr entsprechend mit der Maßgabe, dass die Kommission nach § 15 Absatz 3 Satz 2 des Tierschutzgesetzes zu beteiligen ist. Die Sicherheitsbelange der Bundeswehr sind zu berücksichtigen. Sollen Tierversuche im Auftrag der Bundeswehr durchgeführt werden, so ist die Kommission hiervon ebenfalls zu unterrichten und ihr vor Auftragserteilung Gelegenheit zur Stellungnahme zu geben; § 15 Absatz 1 des Tierschutzgesetzes bleibt unberührt. Die für die Genehmigung des Versuchsvorhabens zuständige Landesbehörde ist davon in Kenntnis zu setzen. Die zuständige Stelle der Bundeswehr sendet auf Anforderung die Stellungnahme zu.

§ 33 Genehmigungsbescheid, Befristung

(1) Der Genehmigungsbescheid ergeht schriftlich oder elektronisch und enthält

1. die Angabe des Leiters des Versuchsvorhabens und seines Stellvertreters,
2. die Angabe, in welchen Einrichtungen oder Betrieben oder, in den Fällen des § 15 Absatz 1 Satz 3, an welchem Ort das Versuchsvorhaben durchgeführt wird,
3. eine Entscheidung darüber, ob und zu welchem Zeitpunkt das Versuchsvorhaben nach § 35 rückblickend zu bewerten ist,
4. gegebenenfalls die Nebenbestimmungen, mit denen die Genehmigung versehen wird, und
5. sofern die zuständige Behörde bei ihrer Entscheidung von den wissenschaftlich begründeten Darlegungen nach § 31 Absatz 1 Satz 2 Nummer 2 und den wissenschaftlichen Beurteilungen nach § 31 Absatz 3 abweicht, unbeschadet der verwaltungsverfahrensrechtlichen Anforderungen zur Begründung eines Verwaltungsaktes eine Darlegung der Gründe.

(2) Die Genehmigung ist auf höchstens fünf Jahre zu befristen. Ist die Genehmigung mit einer Befristung von weniger als fünf Jahren erteilt worden, so ist sie auf, auch formlosen, mit Gründen versehenen Antrag höchstens zweimal um jeweils bis zu einem Jahr zu verlängern, sofern dadurch die Gesamtdauer des genehmigten Versuchsvorhabens fünf Jahre nicht überschreitet und sofern seit der erstmaligen Erteilung oder ersten Verlängerung der Genehmigung keine Änderungen des genehmigten Versuchsvorhabens oder nur solche Änderungen eingetreten sind, die

1. nach § 34 Absatz 1 Satz 1 genehmigt oder
2. nach § 34 Absatz 2 Satz 1 oder Absatz 3 Satz 1 angezeigt und von der zuständigen Behörde nicht beanstandet

worden sind.

§ 34 Genehmigung und Anzeige von Änderungen genehmigter Versuchsvorhaben

(1) Änderungen genehmigter Versuchsvorhaben, die sich nachteilig auf das Wohlergehen der Tiere auswirken können, bedürfen einer Genehmigung. Eine Änderung im Sinne des Satzes 1 liegt insbesondere vor, wenn

1. der Zweck des Versuchsvorhabens nicht beibehalten wird,
2. sich das Maß der bei den verwendeten Tieren verursachten Schmerzen, Leiden und Schäden durch die Änderung erhöhen kann oder
3. die Zahl der verwendeten Tiere wesentlich erhöht wird.

(2) Wechselt der Leiter des Versuchsvorhabens oder sein Stellvertreter, so hat der Genehmigungsinhaber diese Änderung der zuständigen Behörde unverzüglich anzuzeigen. Die Genehmigung ist innerhalb eines Monats ab Eingang der Änderungsanzeige von der zuständigen Behörde zu widerrufen, wenn der Leiter des Versuchsvorhabens oder sein Stellvertreter die Anforderungen des § 8 Absatz 1 Satz 2 Nummer 2 des Tierschutzgesetzes nicht erfüllen.

(3) Andere als die in Absatz 1 Satz 1 genannten Änderungen bedürfen einer Anzeige bei der zuständigen Behörde. Die Änderungen dürfen frühestens zwei Wochen nach Eingang der Anzeige nach Satz 1 vorgenommen werden, es sei denn, die zuständige Behörde hat vorher mitgeteilt, dass gegen die Änderungen keine Einwände bestehen.

§ 35 Rückblickende Bewertung von Versuchsvorhaben

(1) Genehmigt die zuständige Behörde ein Versuchsvorhaben, so kann sie zugleich festlegen, dass das Versuchsvorhaben nach seinem Abschluss durch die zuständige Behörde zu bewerten ist und zu welchem Zeitpunkt diese Bewertung vorzunehmen ist. Eine Bewertung nach Satz 1 ist vorzusehen, wenn das Versuchsvorhaben die Durchführung von

1. Tierversuchen, in denen Primaten verwendet werden,
2. Tierversuchen, die nach Artikel 15 Absatz 1 in Verbindung mit Anhang VIII der Richtlinie 2010/63/EU als „schwer" einzustufen sind, oder
3. Tierversuchen nach § 25 Absatz 2

beinhaltet.

(2) Im Rahmen der Bewertung nach Absatz 1 Satz 1 hat die zuständige Behörde auf Grund von Unterlagen, die der Antragsteller nach § 31 Absatz 1 Satz 1 ihr auf Verlangen vorzulegen hat, soweit sie für die Durchführung der Bewertung erforderlich sind, Folgendes zu prüfen:

1. ob das mitgeteilte Ergebnis mit dem im Antrag nach § 31 Absatz 1 Satz 2 Nummer 1 Buchstabe b angegebenen Zweck des Versuchsvorhabens im Einklang steht,
2. die Schäden, die bei den verwendeten Tieren verursacht worden sind,
3. die Anzahl und die Art der verwendeten Tiere,
4. den Schweregrad der durchgeführten Tierversuche nach Artikel 15 Absatz 1 in Verbindung mit Anhang VIII der Richtlinie 2010/63/EU und
5. ob sich hieraus Schlussfolgerungen im Hinblick auf die Anforderungen gemäß § 7 Absatz 1 Satz 2 und 3 sowie § 7a Absatz 2 Nummer 2, 4 und 5 des Tierschutzgesetzes ergeben.

§ 36 Vereinfachtes Genehmigungsverfahren für Versuchsvorhaben nach § 8a Absatz 1 des Tierschutzgesetzes

(1) Der Antrag auf Genehmigung eines Versuchsvorhabens im vereinfachten Genehmigungsverfahren nach § 8a Absatz 1 des Tierschutzgesetzes ist schriftlich oder elektronisch bei der zuständigen Behörde zu stellen. In dem Antrag sind anzugeben:

1. die Tatsache, dass es sich um einen Antrag auf Genehmigung eines Versuchsvorhabens im vereinfachten Genehmigungsverfahren handelt,
2. die Angaben, Darlegungen und Nachweise, die nach § 31 Absatz 1 Satz 2 erforderlich sind, und
3. im Fall eines Versuchsvorhabens nach § 8a Absatz 1 Satz 1 Nummer 1 des Tierschutzgesetzes zusätzlich die Rechtsgrundlage für die Durchführung des Versuchsvorhabens.

(2) Die zuständige Behörde hat dem Antragsteller innerhalb von

1. 15 Arbeitstagen ab Eingang eines den Anforderungen des Absatzes 1 entsprechenden Antrags
   a) das Ergebnis ihrer Prüfung über das Vorliegen der Voraussetzungen nach § 8 Absatz 1 Satz 2 Nummer 1, 3, 5, 6 und 7 Buchstabe b bis g sowie Nummer 7a des Tierschutzgesetzes und
   b) die Festlegung über die Durchführung einer rückblickenden Bewertung nach § 35,
2. 20 Arbeitstagen ab Eingang eines den Anforderungen des Absatzes 1 entsprechenden Antrags ihre abschließende Entscheidung über den Antrag

mitzuteilen. Die zuständige Behörde kann den in Satz 1 Nummer 1 und 2 genannten Zeitraum jeweils einmalig um bis zu zehn Arbeitstage nach Maßgabe des Absatzes 3 Satz 3 verlängern, soweit der Umfang und die Schwierigkeit der Prüfung des Vorliegens der Voraussetzungen nach

1. § 8 Absatz 1 Satz 2 Nummer 1, 3, 5, 6 und 7 Buchstabe b bis g sowie Nummer 7a des Tierschutzgesetzes im Fall des Satzes 1 Nummer 1 oder
2. § 8a Absatz 1 des Tierschutzgesetzes im Fall des Satzes 1 Nummer 2

dies rechtfertigen.

(3) Nach Eingang eines Antrags nach Absatz 1 hat die zuständige Behörde dem Antragsteller unverzüglich eine Empfangsbestätigung auszustellen. In der Empfangsbestätigung ist anzugeben, dass dem Antragsteller die abschließende Entscheidung über den Antrag innerhalb des in Absatz 2 Satz 1 Nummer 2 genannten Zeitraums mitgeteilt wird. Eine Verlängerung nach Absatz 2 Satz 2 Nummer 2 ist dem Antragsteller spätestens bis zum Ablauf des in Absatz 2 Satz 1 Nummer 2 genannten Zeitraums unter Angabe von Gründen mitzuteilen.

(4) Die zuständige Behörde überprüft einen eingegangenen Antrag nach Absatz 1 Satz 1 unverzüglich nach Eingang auf Vollständigkeit. Sofern dieser den Anforderungen nach Absatz 1 nicht genügt, teilt die zuständige Behörde dies dem Antragsteller unverzüglich unter Benennung der fehlenden Angaben, Darlegungen und Nachweise nach Absatz 1 Satz 2 mit. Der Antragsteller ist darauf hinzuweisen, dass der Beginn der in Absatz 2 Satz 1 genannten Zeiträume den Eingang eines den Anforderungen des Absatzes 1 entsprechenden Antrags voraussetzt.

(5) Die zuständige Behörde kann die Kommission nach § 15 Absatz 1 Satz 2 des Tierschutzgesetzes über Anträge auf Genehmigung von Versuchsvorhaben im vereinfachten Genehmigungsverfahren nach § 8a Absatz 1 des Tierschutzgesetzes unterrichten und ihr Gelegenheit geben, in angemessener Frist Stellung zu nehmen.

(6) Absatz 5 gilt für die zuständige Stelle der Bundeswehr entsprechend mit der Maßgabe, dass die Kommission nach § 15 Absatz 3 Satz 2 des Tierschutzgesetzes beteiligt werden kann. Die Sicherheitsbelange der Bundeswehr sind zu berücksichtigen. Sollen Tierversuche im Auftrag der Bundeswehr durchgeführt werden, so kann die Kommission hiervon ebenfalls unterrichtet werden und ihr kann vor Auftragserteilung Gelegenheit zur Stellungnahme gegeben werden; § 15 Absatz 1 des Tierschutzgesetzes bleibt unberührt. Die für die Genehmigung des Versuchsvorhabens zuständige Landesbehörde ist davon in Kenntnis zu setzen. Die zuständige Stelle der Bundeswehr sendet auf Anforderung die Stellungnahme zu.

(7) § 33 gilt mit der Maßgabe, dass die Genehmigung nach § 33 Absatz 2 Satz 2 bei Vorliegen der weiteren dort genannten Voraussetzungen zu verlängern ist, sofern seit der erstmaligen Erteilung oder ersten Verlängerung der Genehmigung im vereinfachten Genehmigungsverfahren

1. keine Änderungen eingetreten sind oder
2. nur solche Änderungen eingetreten sind, die
    a) nach § 37 Absatz 2 in Verbindung mit § 34 Absatz 1 genehmigt worden sind oder
    b) nach § 34 Absatz 2 Satz 1 oder Absatz 3 Satz 1 angezeigt und von der zuständigen Behörde nicht beanstandet worden sind.

(8) Ein Versuchsvorhaben, für das die Genehmigung nach § 8a Absatz 1 Satz 2 des Tierschutzgesetzes als erteilt gilt, darf nicht nach Ablauf von fünf Jahren nach Ablauf der in Absatz 2 Satz 1 Nummer 2 genannten Frist durchgeführt werden.

§ 37 Sammelgenehmigung und Genehmigung von Änderungen genehmigter Versuchsvorhaben im vereinfachten Genehmigungsverfahren

(1) Ist die Durchführung mehrerer gleichartiger Versuchsvorhaben nach § 8a Absatz 1 Satz 1 des Tierschutzgesetzes beabsichtigt, so genügt die Genehmigung des ersten Versuchsvorhabens im vereinfachten Genehmigungsverfahren, wenn in dem Antrag auf Genehmigung zusätzlich die voraussichtliche Zahl der Versuchsvorhaben angegeben wird. Bis zum 15. Februar eines Jahres hat der Antragsteller der zuständigen Behörde die Zahl der im vorangegangenen Kalenderjahr durchgeführten Versuchsvorhaben sowie Art und Zahl der insgesamt verwendeten Tiere anzugeben.

(2) § 34 Absatz 1 gilt mit der Maßgabe, dass die Änderungen einer erneuten Genehmigung im vereinfachten Genehmigungsverfahren bedürfen.

§ 38 Prüfung der Anzeige von Änderungen von Versuchsvorhaben

Im Fall der Anzeige von Änderungen nach § 34 Absatz 3 prüft die zuständige Behörde innerhalb von zwei Wochen, ob

1. die in § 8 Absatz 1 Satz 2 Nummer 1 bis 8 des Tierschutzgesetzes genannten Voraussetzungen vorliegen oder
2. die Durchführung des Versuchsvorhabens nach § 16a Absatz 2 des Tierschutzgesetzes zu untersagen ist.

§ 39 Anzeige von Versuchsvorhaben an Zehnfußkrebsen

(1) In der Anzeige eines Versuchsvorhabens nach § 8a Absatz 3 des Tierschutzgesetzes sind anzugeben:

1. der Zweck des Versuchsvorhabens,
2. die Art der für das Versuchsvorhaben vorgesehenen Tiere,
3. die Art und die Durchführung der beabsichtigten Tierversuche, einschließlich der Betäubung,
4. der Ort, der Zeitpunkt des Beginns und die voraussichtliche Dauer des Versuchsvorhabens und
5. der Name, die Anschrift und die Sachkunde des Leiters des Versuchsvorhabens, seines Stellvertreters und der durchführenden Personen sowie die für die Nachbehandlung in Frage kommenden Personen.

Die Anzeige hat schriftlich oder elektronisch zu erfolgen. § 37 Absatz 1 gilt entsprechend. Ändert sich ein nach Satz 1 Nummer 1 bis 3 in der Anzeige angegebener Sachverhalt während des Versuchsvorhabens, ist die Änderung unverzüglich der zuständigen Behörde anzuzeigen.

(2) Mit der Durchführung des Versuchsvorhabens darf nicht vor Ablauf von zwei Wochen ab Eingang einer den Anforderungen des Absatzes 1 entsprechenden Anzeige bei der zuständigen Behörde begonnen werden, es sei denn die zuständige Behörde hat zuvor mitgeteilt, dass gegen die Durchführung keine Einwände bestehen. Die in Satz 1 genannte Frist kann von der zuständigen Behörde bei Bedarf auf bis zu vier Wochen verlängert werden; die Verlängerung ist dem Antragsteller spätestens bis zum Ablauf der in Satz 1 genannten Frist unter Angabe von Gründen mitzuteilen.

(2a) Nach Eingang einer Anzeige nach § 8a Absatz 3 des Tierschutzgesetzes hat die zuständige Behörde dem Anzeigenden unverzüglich eine Empfangsbestätigung auszustellen. In der Empfangsbestätigung ist der Tag des Einganges der Anzeige anzugeben und auf die Frist nach Absatz 2 hinzuweisen.

(2b) Ein nach § 8a Absatz 3 des Tierschutzgesetzes angezeigtes Versuchsvorhaben darf nicht durchgeführt werden nach Ablauf von fünf Jahren

1. nach Ablauf der in Absatz 2 genannten Frist oder
2. nach Eingang der Mitteilung nach Absatz 2.

(3) Im Falle des Eingangs einer Anzeige nach Absatz 1 Satz 1 prüft die zuständige Behörde, ob im Hinblick auf das angezeigte Versuchsvorhaben die Einhaltung der Vorschriften des § 7 Absatz 1 Satz 2 bis 4 und § 7a Absatz 1 und 2 Nummer 1, 2, 4 und 5 des Tierschutzgesetzes, des § 20 Absatz 1 und der §§ 27 und 28 Absatz 3 und 4 sichergestellt ist oder ob die Durchführung des Versuchsvorhabens nach § 16a Absatz 2 des Tierschutzgesetzes zu untersagen ist.

§ 40 Aufbewahrungspflicht

Der Inhaber einer Genehmigung oder, im Falle von Versuchsvorhaben nach § 8a Absatz 3 des Tierschutzgesetzes, der Anzeigende hat

1. eine Kopie des Antrags nach § 31 und den Genehmigungsbescheid nach § 33 oder, im Fall von Versuchsvorhaben nach § 8a Absatz 1 Satz 1 des Tierschutzgesetzes, eine Kopie des Antrags nach § 36 Absatz 1 und des Genehmigungsbescheids nach § 33 in Verbindung mit § 36 Absatz 6 oder im Fall von Versuchsvorhaben nach § 8a Absatz 3 des Tierschutzgesetzes, eine Kopie der Anzeige nach § 39 Absatz 1 Satz 1 sowie
2. alle sonstigen Dokumente, die ihm im Zusammenhang mit der Genehmigung oder Anzeige und der Durchführung des Versuchsvorhabens von der zuständigen Behörde übermittelt worden sind,

mindestens drei Jahre über das Ende der Geltungsdauer der Genehmigung oder, bei Versuchsvorhaben nach § 8a Absatz 3 des Tierschutzgesetzes, über den Ablauf der in § 39 Absatz 2b genannten Frist hinaus aufzubewahren. Abweichend von Satz 1 darf im Fall der elektronischen Übermittlung der dort genannten Dokumente die Aufbewahrung dieser Dokumente durch Speicherung auf einem dauerhaften Datenträger erfolgen. Im Falle von Versuchsvorhaben, die einer Bewertung nach § 35 unterzogen werden sollen, sind die in Satz 1 genannten Dokumente bis zum Abschluss der Bewertung aufzubewahren, soweit der Abschluss der Bewertung erst nach Ablauf der in Satz 1 genannten Frist erfolgt.

Fußnote

(+++ § 40: Zur Anwendung vgl. § 48 Abs. 6 +++)

§ 41 Veröffentlichung von Zusammenfassungen

(1) Die zuständige Behörde übermittelt dem Bundesinstitut für Risikobewertung (Bundesinstitut) innerhalb von drei Monaten nach Erteilung einer Genehmigung nach § 8 Absatz 1 Satz 1 des Tierschutzgesetzes eine Zusammenfassung zu dem genehmigten Versuchsvorhaben zum Zwecke der Veröffentlichung durch das Bundesinstitut. In der Zusammenfassung ist auf der Grundlage der Angaben im Genehmigungsantrag Folgendes darzustellen:

1. die Zwecke des Versuchsvorhabens,
2. der zu erwartende Nutzen des Versuchsvorhabens,

3. die zu erwartenden Schäden bei den zur Verwendung vorgesehenen Tieren,
4. die Anzahl und die Art der zur Verwendung vorgesehenen Tiere und
5. die Erfüllung der Anforderungen des § 7 Absatz 1 Satz 2 und 3 sowie des § 7a Absatz 2 Nummer 2, 4 und 5 des Tierschutzgesetzes.

Die Zusammenfassung darf keine einrichtungs- oder personenbezogenen Daten enthalten. Die Vorschriften zum Schutz des geistigen Eigentums und zum Schutz von Betriebs- und Geschäftsgeheimnissen bleiben unberührt.

(2) Die Zusammenfassung wird innerhalb von zwölf Monaten nach der Übermittlung durch die zuständige Behörde durch das Bundesinstitut im Internet veröffentlicht. Die entsprechende Internetseite wird durch das Bundesinstitut im Bundesanzeiger bekannt gemacht.

(3) Die Übermittlung der Zusammenfassung nach Absatz 1 Satz 1 erfolgt auch zum Zwecke der Weiterleitung an die Europäische Kommission. Das Bundesinstitut leitet die Zusammenfassung einschließlich notwendiger Aktualisierungen innerhalb von drei Monaten nach der Übermittlung durch die zuständigen Behörden auf elektronischem Wege an die Europäische Kommission weiter.

§ 42 Tierversuchskommissionen

(1) Die Mehrheit der Mitglieder der nach § 15 Absatz 1 Satz 2 und Absatz 3 Satz 2 des Tierschutzgesetzes einzuberufenden Kommissionen muss die für die Beurteilung von Tierversuchen erforderlichen Fachkenntnisse der Veterinärmedizin, der Medizin oder einer naturwissenschaftlichen Fachrichtung haben.

(2) In die Kommissionen nach § 15 Absatz 1 Satz 2 des Tierschutzgesetzes sind auch Mitglieder zu berufen, die auf Grund von Vorschlägen der Tierschutzorganisationen ausgewählt worden sind und auf Grund ihrer Erfahrungen zur Beurteilung von Tierschutzfragen geeignet sind; die Zahl dieser Mitglieder muss mindestens ein Drittel der Kommissionsmitglieder betragen.

(3) In die Kommissionen nach § 15 Absatz 3 Satz 2 des Tierschutzgesetzes sollen auch Mitglieder berufen werden, die auf Grund von Vorschlägen der Tierschutzorganisationen ausgewählt worden sind und auf Grund ihrer Erfahrungen zur Beurteilung von Tierschutzfragen geeignet sind.

§ 43 Unterrichtung des Bundesministeriums

Die nach Landesrecht zuständigen Behörden unterrichten das Bundesministerium über Fälle grundsätzlicher Bedeutung bei der Genehmigung von Versuchsvorhaben, insbesondere über die Fälle, in denen die Genehmigung von Versuchsvorhaben mit der Begründung versagt worden ist, dass die Voraussetzungen des § 7a Absatz 2 Nummer 3 des Tierschutzgesetzes nicht erfüllt waren, oder in denen die Kommission nach § 15 Absatz 1 des Tierschutzgesetzes oder der Tierschutzbeauftragte Bedenken hinsichtlich des Vorliegens dieser Voraussetzungen erhoben haben. Personenbezogene Daten dürfen nicht übermittelt werden.

Abschnitt 3
Ordnungswidrigkeiten

§ 44 Ordnungswidrigkeiten

(1) Ordnungswidrig im Sinne des § 18 Absatz 1 Nummer 3 Buchstabe a des Tierschutzgesetzes handelt, wer vorsätzlich oder fahrlässig

1. entgegen § 15 Absatz 1 Satz 1 oder § 17 Absatz 2 Satz 1 einen Tierversuch durchführt,
2. entgegen § 17 Absatz 5 ein Mittel anwendet,
3. entgegen § 30 Absatz 1 die Einhaltung der Vorschriften des § 15 Absatz 1 Satz 1 nicht sicherstellt oder
4. entgegen § 30 Absatz 1 die Einhaltung der Vorschriften des § 17 Absatz 2 Satz 1 oder Absatz 4 nicht sicherstellt.

(2) Ordnungswidrig im Sinne des § 18 Absatz 1 Nummer 3 Buchstabe b des Tierschutzgesetzes handelt, wer vorsätzlich oder fahrlässig

1. entgegen § 2 Absatz 1 Satz 1 ein Wirbeltier oder einen Kopffüßer tötet,
2. entgegen § 5 Absatz 1 Satz 1 einen Tierschutzbeauftragten nicht oder nicht rechtzeitig bestellt oder eine Anzeige nicht, nicht richtig oder nicht rechtzeitig erstattet,

2a. entgegen § 6 Absatz 1 Satz 1 einen Tierschutzausschuss nicht oder nicht rechtzeitig bestellt,

3. entgegen § 7 Absatz 1 Satz 1 ein Kontrollbuch nicht, nicht richtig, nicht vollständig oder nicht in der vorgeschriebenen Weise führt,

4. entgegen § 7 Absatz 2 eine dort genannte Aufzeichnung nicht oder nicht mindestens fünf Jahre aufbewahrt oder nicht oder nicht rechtzeitig vorlegt,

5. entgegen § 8 Absatz 1 Satz 1 eine Aufzeichnung nicht, nicht richtig oder nicht vollständig führt,

6. entgegen § 8 Absatz 2 Nummer 2 eine Aufzeichnung nicht, nicht richtig, nicht vollständig oder nicht rechtzeitig übergibt,

7. entgegen § 8 Absatz 2 Nummer 4 eine Aufzeichnung nicht oder nicht mindestens drei Jahre aufbewahrt oder nicht oder nicht rechtzeitig vorlegt,

8. entgegen § 9 ein dort genanntes Tier nicht, nicht richtig, nicht in der vorgeschriebenen Weise oder nicht rechtzeitig kennzeichnet,

9. entgegen § 9 Absatz 2 den dort genannten Nachweis nicht erbringt,

9a. entgegen § 19 Absatz 1 Satz 1, § 20 Absatz 1 Satz 1, § 21 Satz 1 oder § 24 Absatz 1 ein dort genanntes Tier, einen Kopffüßer oder einen Primaten verwendet,

10. entgegen § 30 Absatz 1 die Einhaltung der Vorschriften des § 29 Absatz 1 nicht sicherstellt,

11. entgegen § 30 Absatz 1 die Einhaltung der Vorschriften des § 29 Absatz 2 nicht sicherstellt,

12. entgegen § 37 Absatz 1 Satz 2, auch in Verbindung mit § 39 Absatz 1 Satz 3, eine Angabe nicht, nicht richtig, nicht vollständig oder nicht rechtzeitig macht,

13. entgegen § 37 Absatz 2 eine Änderung vornimmt,

14. entgegen § 39 Absatz 2 Satz 1 ein Versuchsvorhaben beginnt oder

15. entgegen § 40 ein dort genanntes Dokument nicht oder nicht für die vorgeschriebene Dauer aufbewahrt.

Abschnitt 4
Übergangs- und Schlussbestimmungen

§ 45 Aufgaben nach Artikel 49 der Richtlinie 2010/63/EU

Das Bundesinstitut berät die zuständigen Behörden und die Tierschutzausschüsse nach § 6 in Angelegenheiten, die mit dem Erwerb, der Zucht, der Unterbringung oder der Pflege von Wirbeltieren und Kopffüßern nach § 1 Absatz 1 oder der Verwendung von Wirbeltieren und Kopffüßern in Tierversuchen zusammenhängen, und gewährleistet, dass diesbezüglich ein Austausch über bewährte Praktiken stattfindet. Darüber hinaus tauscht es mit in anderen Mitgliedstaaten der Europäischen Union eingerichteten Ausschüssen nach Artikel 49 Absatz 1 der Richtlinie 2010/63/EU Informationen über

1. die Arbeit der Tierschutzausschüsse nach § 6 und

2. die Beurteilung von Versuchsvorhaben,

einschließlich diesbezüglicher bewährter Praktiken, aus.

§ 46 Beratung zu Alternativen zu Tierversuchen

Das Bundesinstitut berät die zuständigen Behörden in Angelegenheiten, die mit Alternativen zu Tierversuchen zusammenhängen.

§ 47 Unberührtheitsklausel

Die Vorschriften des Naturschutzrechts, des Jagdrechts, des Umweltrechts und des Fischereirechts bleiben unberührt.

§ 48 Übergangsvorschriften

(1) Die §§ 1 und 3 bis 6 gelten für Einrichtungen und Betriebe, in denen am 12. August 2013 Wirbeltiere oder Kopffüßer,

1. die zur Verwendung in Tierversuchen bestimmt sind oder

2. deren Gewebe oder Organe dazu bestimmt sind, zu wissenschaftlichen Zwecken verwendet zu werden,

gehalten werden, ab dem 1. Januar 2014. Satz 1 gilt auch für Einrichtungen und Betriebe, in denen die dort genannten Tiere gezüchtet oder zum Zwecke der Abgabe an Dritte gehalten werden. Für Einrichtungen und Betriebe nach Satz 1, in denen Tierversuche an Wirbeltieren durchgeführt werden, ist bis zum 31. Dezember 2013 § 8b des Tierschutzgesetzes in der bis zum 13. Juli 2013 geltenden Fassung weiter anzuwenden.

(2) Wer nach § 8b Absatz 2 des Tierschutzgesetzes in der am 12. Juli 2013 geltenden Fassung am 12. Juli 2013 befugt ist, als Tierschutzbeauftragter tätig zu sein, behält diese Befugnis, solange er die Tätigkeit weiter ausübt.

(3) Wer nach § 9 Absatz 1 des Tierschutzgesetzes in der am 12. Juli 2013 geltenden Fassung im Rahmen seiner am 12. Juli 2013 ausgeübten Tätigkeit befugt ist, Tierversuche durchzuführen, behält diese Befugnis, solange er die Tätigkeit weiter ausübt.

(4) Die §§ 14 bis 41 und § 44 gelten nicht für Tierversuche,

1. deren Genehmigung vor dem Inkrafttreten dieser Verordnung nach den Vorschriften des Tierschutzgesetzes in der bis zum 13. Juli 2013 geltenden Fassung unter Einhaltung der Anforderungen nach dessen § 8 Absatz 2 beantragt oder
2. deren Durchführung vor dem Inkrafttreten dieser Verordnung nach den Vorschriften des Tierschutzgesetzes in der bis zum 13. Juli 2013 geltenden Fassung angezeigt und von der zuständigen Behörde nicht beanstandet

worden ist.

(5) Für Tierversuche,

1. deren Genehmigung vor dem 1. Dezember 2021 erteilt worden ist oder
2. deren Durchführung vor dem 1. Dezember 2021 nach den Vorschriften des Tierschutzgesetzes in der bis Ablauf des 1. Dezember 2021 anzuwendenden Fassung und nach den Vorschriften dieser Verordnung in der bis zum 1. Dezember 2021 geltenden Fassung angezeigt und von der zuständigen Behörde nicht beanstandet worden ist,

sind abweichend von den §§ 31 bis 38 bis zum 1. Dezember 2023 die Vorschriften dieser Verordnung in der bis zum 1. Dezember 2021 geltenden Fassung weiter anzuwenden.

(6) Für Tierversuche, deren Durchführung vor dem 1. Dezember 2021 nach § 8a Absatz 1 des Tierschutzgesetzes in der bis zum 1. Dezember 2021 anzuwendenden Fassung und den Vorschriften dieser Verordnung in der bis zum 1. Dezember 2021 geltenden Fassung angezeigt und von der zuständigen Behörde nicht beanstandet worden ist, ist § 40 in der bis zum 1. Dezember 2021 geltenden Fassung weiter anzuwenden.

Anlage 1 Kenntnisse und Fähigkeiten, die für die Pflege oder das Töten von Tieren oder die Planung oder die Durchführung von Tierversuchen erforderlich sind

(Fundstelle: BGBl. I 2013, 3140 - 3141;
bzgl. der einzelnen Änderungen vgl. Fußnote)

Abschnitt 1
Pflege von Tieren

1. Geltende Rechtsvorschriften zur Haltung und Pflege von Tieren, die dazu bestimmt sind, in Tierversuchen verwendet zu werden, oder deren Gewebe oder Organe dazu bestimmt sind, zu wissenschaftlichen Zwecken verwendet zu werden.
2. Grundlagen der Biologie und angemessene artspezifische Biologie in Bezug auf Anatomie und physiologische Merkmale.
3. Tierverhalten und Haltungsanforderungen und -methoden, einschließlich Anreicherung der Haltungseinrichtungen (allgemein und artspezifisch).
4. Gesunderhaltung und Hygiene des Tierbestands.
5. Erkennung artspezifischer Schmerzen und Leiden der am häufigsten für Tierversuche verwendeten Arten.

6. Anforderung gemäß § 7 Absatz 1 Satz 2 Nummer 2 des Tierschutzgesetzes.
7. Verhaltensgerechter Umgang mit Tieren.
8. Ethik in Bezug auf die Beziehung zwischen Mensch und Tier sowie intrinsischer Wert des Lebens.
9. Anforderungen des Prinzips der Unerlässlichkeit nach § 7 Absatz 1 Satz 2 und 3 sowie § 7a Absatz 2 Nummer 2, 4 und 5 des Tierschutzgesetzes.

Abschnitt 2
Töten von Tieren

1. Geltende Rechtsvorschriften zum Töten von Tieren zu wissenschaftlichen Zwecken oder von Tieren, die dazu bestimmt sind, in Tierversuchen verwendet zu werden.
2. Ethik in Bezug auf die Beziehung zwischen Mensch und Tier, intrinsischer Wert des Lebens und Argumente für und gegen die Verwendung von Tieren zu wissenschaftlichen Zwecken.
3. Grundlagen der Biologie und angemessene artspezifische Biologie in Bezug auf Anatomie und physiologische Merkmale.
4. Grundkenntnisse des Verhaltens der Tiere.
5. Grundkenntnisse der Physik und Chemie, soweit diese für die betreffenden Tötungsverfahren notwendig sind.
6. Eignung und Kapazität der jeweiligen Tötungsverfahren.
7. Betäubung, schmerzlindernde Methoden und Töten einschließlich der Verfahren, die für die Tiere die geringste Belastung bedeuten.
8. Gegebenenfalls artspezifische Handhabungsmethoden.
9. Ordnungsgemäße Durchführung der Tötung und gegebenenfalls vorhergehende Betäubung der Tiere unter Zufügung geringstmöglicher Schmerzen oder Leiden.
10. Wartung der für die Tötung und gegebenenfalls vorhergehende Betäubung notwendigen Geräte oder Anlagen.
11. Erkennen artspezifischer Schmerzen und Leiden der am häufigsten für Tierversuche verwendeten Arten.
12. Anforderungen des Prinzips der Unerlässlichkeit nach § 7 Absatz 1 Satz 2 und 3 sowie § 7a Absatz 2 Nummer 2, 4 und 5 des Tierschutzgesetzes.

Abschnitt 3
Planung und Durchführung von Tierversuchen

1. Geltende Rechtsvorschriften zur Durchführung von Tierversuchen.
2. Ethik in Bezug auf die Beziehung zwischen Mensch und Tier, intrinsischer Wert des Lebens und Argumente für und gegen die Verwendung von Tieren zu wissenschaftlichen Zwecken.
3. Grundlagen der Biologie und angemessene artspezifische Biologie in Bezug auf Anatomie, physiologische Merkmale, Zucht, Genetik und genetische Veränderung.
4. Tierverhalten und Haltungsanforderungen und -methoden, einschließlich Anreicherung der Haltungseinrichtungen (allgemein und artspezifisch).
5. Gesunderhaltung und Hygiene des Tierbestands.
6. Artspezifische Handhabungs- und Versuchsmethoden.
7. Erkennung artspezifischer Schmerzen und Leiden der am häufigsten für Tierversuche verwendeten Arten.
8. Anwendung möglichst schmerzloser Endpunkte.
9. Anforderungen des Prinzips der Unerlässlichkeit von Tierversuchen gemäß § 7 Absatz 1 Satz 2 und 3 sowie § 7a Absatz 2 Nummer 2, 4 und 5 des Tierschutzgesetzes.
10. Gegebenenfalls Planung von Verfahren und Projekten.
11. Relevante Versuchstechniken und operative Eingriffe.

12. Recherche und Auswertung wissenschaftlicher Literatur einschließlich solcher zu Alternativen zum Tierversuch.
13. Betäubung und schmerzlindernde Methoden.
14. Soweit im Rahmen der Durchführung auch die Tötung der Tiere vorgesehen ist, die Kenntnisse und Fähigkeiten nach Abschnitt 2.
15. Biometrische Statistik.

Anlage 2 (zu § 2 Absatz 2)
Tötungsverfahren

(Fundstelle: BGBl. I 2013, 3142 - 3143)

1. Zur Tötung von Tieren einer der in Zeile 1 der Tabelle genannten Tierkategorien dürfen nur diejenigen Verfahren angewendet werden, die in Spalte 1 Zeile 2 bis 9 gelistet sind und die in der die jeweilige Tierkategorie betreffenden Spalte mit einem Kreuz (+) bezeichnet sind, unter Beachtung der in den Anmerkungen enthaltenen Maßgaben. Hierbei ist immer die am wenigsten belastende Methode zu wählen, soweit dieses mit dem Versuchszweck vereinbar ist.

	Fische	Amphibien	Reptilien	Vögel	Nagetiere	Kaninchen	Hunde, Katzen, Frettchen und Füchse	Große Säugetiere	Primaten
Überdosis eines Betäubungsmittels	+[1]	+[1]	+[1]	+[1]	+[1]	+[1]	+[1]	+[1]	+[1]
Bolzenschuss			+[2]			+		+	
Kohlendioxidexposition				+	+[3]				
Zervikale Dislokation				+[4]	+[5]	+[6]			
Gehirnerschütterung/stumpfer Schlag auf den Kopf	+	+	+	+[7]	+[8]	+[9]	+[10]		
Dekapitation				+[11]	+[12]				
Elektrische Betäubung	+[13]	+[13]		+[13]		+[13]	+[13]	+[13]	
Inhalation von Inertgasen (Argon, Stickstoff)				+	+			+[14]	
Pistolen- oder Gewehrschuss mit angemessenen Waffen und angemessener Munition			+[15]				+[16]	+[15]	

Anmerkungen:

[1] Das Verfahren muss in Verbindung mit einem vorherigen Sedieren der Tiere eingesetzt werden, es sei denn, dies ist unangemessen.

[2] Das Verfahren darf nur bei großen Reptilien angewendet werden.

[3] Das Verfahren darf nur unter schrittweiser Befüllung des Behältnisses angewendet werden. Das Verfahren darf nicht bei Föten und Neugeborenen angewendet werden.

[4] Das Verfahren darf nur bei Vögeln mit einem Gewicht von unter 1 kg angewendet werden. Vögel mit einem Gewicht von über 250 g müssen zuvor sediert werden.

[5] Das Verfahren darf nur bei Nagetieren mit einem Gewicht von unter 1 kg angewendet werden. Nagetiere mit einem Gewicht von über 150 g müssen zuvor sediert werden.

[6] Das Verfahren darf nur bei Kaninchen mit einem Gewicht von unter 1 kg angewendet werden. Kaninchen mit einem Gewicht von über 150 g müssen zuvor sediert werden.

[7] Das Verfahren darf nur bei Vögeln mit einem Gewicht von unter 5 kg angewendet werden.

[8] Das Verfahren darf nur bei Nagetieren mit einem Gewicht von unter 1 kg angewendet werden.

[9] Das Verfahren darf nur bei Kaninchen mit einem Gewicht von unter 5 kg angewendet werden.

[10] Das Verfahren darf nur bei Neugeborenen angewendet werden.

[11] Das Verfahren darf nur bei Vögeln mit einem Gewicht von unter 250 g angewendet werden.

[12] Das Verfahren darf nur angewendet werden, wenn die Anwendung anderer Verfahren nicht möglich ist.

[13] Für die Anwendung des Verfahrens sind dafür geeignete Anlagen und Geräte erforderlich.

[14] Das Verfahren darf nur bei Schweinen angewendet werden.

[15] Das Verfahren darf nur in den Fällen des § 2 Absatz 1 Satz 3 und nur von einem erfahrenen Schützen angewendet werden.

[16] Das Verfahren darf nur in den Fällen des § 2 Absatz 1 Satz 3 und nur von einem erfahrenen Schützen angewendet werden. Darüber hinaus darf es nur dann angewendet werden, wenn die Anwendung anderer Verfahren nicht möglich ist.

2. Die Tötung der Tiere unter Anwendung der unter Nummer 1 genannten Verfahren ist durch eines der folgenden Verfahren abzuschließen:

   a) Bestätigen des endgültigen Kreislaufstillstands,
   b) Zerstören des Gehirns,
   c) Durchtrennen des Rückenmarks im Genick,
   d) Entbluten oder
   e) Bestätigen des Eintritts der Totenstarre.
