# Supplementary material for: Compensatory Fatty Acid Metabolism and Hepatic Gene Expression in African Catfish (Clarias gariepinus) Fed Marine‐Ingredient‐Free Circular Diets Low in EPA and DHA
Source: Aquac Nutr. 2026 Jun 11;2026:4162985. doi: 10.1155/anu/4162985 (PMC13259715; doi:10.1155/anu/4162985)
Supplement: Supplementary file 2 — Supporting Information 2 Directive 2010/63/EU (Annex 4) https://eur-lex.europa.eu/legal-content/EN/TXT/PDF/?uri=CELEX:32010L0063. [file ANU-2026-4162985-s001.pdf]

DIRECTIVES

DIRECTIVE 2010/63/EU OF THE EUROPEAN PARLIAMENT AND OF THE COUNCIL

of 22 September 2010

on the protection of animals used for scientific purposes

(Text with EEA relevance)

THE EUROPEAN PARLIAMENT AND THE COUNCIL OF THE EUROPEAN UNION,

Having regard to the Treaty on the Functioning of the European Union, and in particular Article 114 thereof,

Having regard to the proposal from the European Commission,

Having regard to the opinion of the European Economic and Social Committee ([1]),

After consulting the Committee of the Regions,

Acting in accordance with the ordinary legislative procedure ([2]),

Whereas:

(1) On 24 November 1986 the Council adopted Directive 86/609/EEC ([3]) in order to eliminate disparities between laws, regulations and administrative provisions of the Member States regarding the protection of animals used for experimental and other scientific purposes. Since the adoption of that Directive, further disparities between Member States have emerged. Certain Member States have adopted national implementing measures that ensure a high level of protection of animals used for scientific purposes, while others only apply the minimum requirements laid down in Directive 86/609/EEC. These disparities are liable to constitute barriers to trade in products and substances the development of which involves experiments on animals. Accordingly, this Directive should provide for more detailed rules in order to reduce such disparities by approximating the rules applicable in that area and to ensure a proper functioning of the internal market.

(2) Animal welfare is a value of the Union that is enshrined in Article 13 of the Treaty on the Functioning of the European Union (TFEU).

(3) On 23 March 1998 the Council adopted Decision 1999/575/EC concerning the conclusion by the Community of the European Convention for the protection of vertebrate animals used for experimental and other scientific purposes ([4]). By becoming party to that Convention, the Community acknowledged the importance of the protection and welfare of animals used for scientific purposes at international level.

(4) The European Parliament in its resolution of 5 December 2002 on Directive 86/609/EEC called for the Commission to come forward with a proposal for a revision of that Directive with more stringent and transparent measures in the area of animal experimentation.

(5) On 15 June 2006, the Fourth Multilateral Consultation of Parties to the European Convention for the protection of vertebrate animals used for experimental and other scientific purposes adopted a revised Appendix A to that Convention, which set out guidelines for the accommodation and care of experimental animals. Commission Recommendation 2007/526/EC of 18 June 2007 on guidelines for the accommodation and care of animals used for experimental and other scientific purposes ([5]) incorporated those guidelines.

(6) New scientific knowledge is available in respect of factors influencing animal welfare as well as the capacity of animals to sense and express pain, suffering, distress and lasting harm. It is therefore necessary to improve the welfare of animals used in scientific procedures by raising the minimum standards for their protection in line with the latest scientific developments.

(7) Attitudes towards animals also depend on national perceptions, and there is a demand in certain Member States to maintain more extensive animal-welfare rules than those agreed upon at the level of the Union. In the interests of the animals, and provided it does not affect the functioning of the internal market, it is appropriate to allow the Member States certain flexibility to maintain national rules aimed at more extensive protection of animals in so far as they are compatible with the TFEU.

([1]) OJ C 277, 17.11.2009, p. 51.
([2]) Position of the European Parliament of 5 May 2009 (OJ C 212 E, 5.8.2010, p. 170), position of the Council of 13 September 2010 (not yet published in the Official Journal) and position of the European Parliament of 8 September 2010 (not yet published in the Official Journal).
([3]) OJ L 358, 18.12.1986, p. 1.
([4]) OJ L 222, 24.8.1999, p. 29.
([5]) OJ L 197, 30.7.2007, p. 1.

(8) In addition to vertebrate animals including cyclostomes, cephalopods should also be included in the scope of this Directive, as there is scientific evidence of their ability to experience pain, suffering, distress and lasting harm.

(9) This Directive should also cover foetal forms of mammals, as there is scientific evidence showing that such forms in the last third of the period of their development are at an increased risk of experiencing pain, suffering and distress, which may also affect negatively their subsequent development. Scientific evidence also shows that procedures carried out on embryonic and foetal forms at an earlier stage of development could result in pain, suffering, distress or lasting harm, should the developmental forms be allowed to live beyond the first two thirds of their development.

(10) While it is desirable to replace the use of live animals in procedures by other methods not entailing the use of live animals, the use of live animals continues to be necessary to protect human and animal health and the environment. However, this Directive represents an important step towards achieving the final goal of full replacement of procedures on live animals for scientific and educational purposes as soon as it is scientifically possible to do so. To that end, it seeks to facilitate and promote the advancement of alternative approaches. It also seeks to ensure a high level of protection for animals that still need to be used in procedures. This Directive should be reviewed regularly in light of evolving science and animal-protection measures.

(11) The care and use of live animals for scientific purposes is governed by internationally established principles of replacement, reduction and refinement. To ensure that the way in which animals are bred, cared for and used in procedures within the Union is in line with that of the other international and national standards applicable outside the Union, the principles of replacement, reduction and refinement should be considered systematically when implementing this Directive. When choosing methods, the principles of replacement, reduction and refinement should be implemented through a strict hierarchy of the requirement to use alternative methods. Where no alternative method is recognised by the legislation of the Union, the numbers of animals used may be reduced by resorting to other methods and by implementing testing strategies, such as the use of *in vitro* and other methods that would reduce and refine the use of animals.

(12) Animals have an intrinsic value which must be respected. There are also the ethical concerns of the general public as regards the use of animals in procedures. Therefore, animals should always be treated as sentient creatures and their use in procedures should be restricted to areas which may ultimately benefit human or animal health, or the environment. The use of animals for scientific or educational purposes should therefore only be considered where a non-animal alternative is unavailable. Use of animals for scientific procedures in other areas under the competence of the Union should be prohibited.

(13) The choice of methods and the species to be used have a direct impact on both the numbers of animals used and their welfare. The choice of methods should therefore ensure the selection of the method that is able to provide the most satisfactory results and is likely to cause the minimum pain, suffering or distress. The methods selected should use the minimum number of animals that would provide reliable results and require the use of species with the lowest capacity to experience pain, suffering, distress or lasting harm that are optimal for extrapolation into target species.

(14) The methods selected should avoid, as far as possible, death as an end-point due to the severe suffering experienced during the period before death. Where possible, it should be substituted by more humane end-points using clinical signs that determine the impending death, thereby allowing the animal to be killed without any further suffering.

(15) The use of inappropriate methods for killing an animal can cause significant pain, distress and suffering to the animal. The level of competence of the person carrying out this operation is equally important. Animals should therefore be killed only by a competent person using a method that is appropriate to the species.

(16) It is necessary to ensure that the use of animals in procedures does not pose a threat to biodiversity. Therefore, the use of endangered species in procedures should be limited to a strict minimum.

(17) Having regard to the present state of scientific knowledge, the use of non-human primates in scientific procedures is still necessary in biomedical research. Due to their genetic proximity to human beings and to their highly developed social skills, the use of non-human primates in scientific procedures raises specific ethical and practical problems in terms of meeting their behavioural, environmental and social needs in a laboratory environment. Furthermore, the use of non-human primates is of the greatest concern to the public. Therefore the use of non-human primates should be permitted only in those biomedical areas essential for the benefit of human beings, for which no other

alternative replacement methods are yet available. Their use should be permitted only for basic research, the preservation of the respective non-human primate species or when the work, including xenotransplantation, is carried out in relation to potentially life-threatening conditions in humans or in relation to cases having a substantial impact on a person's day-to-day functioning, i.e. debilitating conditions.

(18) The use of great apes, as the closest species to human beings with the most advanced social and behavioural skills, should be permitted only for the purposes of research aimed at the preservation of those species and where action in relation to a life-threatening, debilitating condition endangering human beings is warranted, and no other species or alternative method would suffice in order to achieve the aims of the procedure. The Member State claiming such a need should provide information necessary for the Commission to take a decision.

(19) The capture of non-human primates from the wild is highly stressful for the animals concerned and carries an elevated risk of injury and suffering during capture and transport. In order to end the capturing of animals from the wild for breeding purposes, only animals that are the offspring of an animal which has been bred in captivity, or that are sourced from self-sustaining colonies, should be used in procedures after an appropriate transition period. A feasibility study should be carried out to that effect and the transition period adopted if necessary. The feasibility of moving towards sourcing non-human primates only from self-sustaining colonies as an ultimate goal should also be examined.

(20) There is a need for certain species of vertebrate animals used in procedures to be bred specifically for that purpose so that their genetic, biological and behavioural background is well-known to persons undertaking the procedures. Such knowledge both increases the scientific quality and reliability of the results and decreases the variability, ultimately resulting in fewer procedures and reduced animal use. Furthermore, for reasons of animal welfare and conservation, the use of animals taken from the wild in procedures should be limited to cases where the purpose of the procedures cannot be achieved using animals bred specifically for use in procedures.

(21) Since the background of stray and feral animals of domestic species is not known, and since capture and placement into establishments increases distress for such animals, they should not, as a general rule, be used in procedures.

(22) To enhance transparency, facilitate the project authorisation, and provide tools for monitoring compliance, a severity classification of procedures should be introduced on the basis of estimated levels of pain, suffering, distress and lasting harm that is inflicted on the animals.

(23) From an ethical standpoint, there should be an upper limit of pain, suffering and distress above which animals should not be subjected in scientific procedures. To that end, the performance of procedures that result in severe pain, suffering or distress, which is likely to be long-lasting and cannot be ameliorated, should be prohibited.

(24) When developing a common format for reporting purposes, the actual severity of the pain, suffering, distress or lasting harm experienced by the animal should be taken into account rather than the predicted severity at the time of the project evaluation.

(25) The number of animals used in procedures could be reduced by performing procedures on animals more than once, where this does not detract from the scientific objective or result in poor animal welfare. However, the benefit of reusing animals should be balanced against any adverse effects on their welfare, taking into account the lifetime experience of the individual animal. As a result of this potential conflict, the reuse of animals should be considered on a case-by-case basis.

(26) At the end of the procedure, the most appropriate decision should be taken as regards the future of the animal on the basis of animal welfare and potential risks to the environment. The animals whose welfare would be compromised should be killed. In some cases, animals should be returned to a suitable habitat or husbandry system or animals such as dogs and cats should be allowed to be rehomed in families as there is a high level of public concern as to the fate of such animals. Should Member States allow rehoming, it is essential that the breeder, supplier or user has a scheme in place to provide appropriate socialisation to those animals in order to ensure successful rehoming as well as to avoid unnecessary distress to the animals and to guarantee public safety.

(27) Animal tissue and organs are used for the development of *in vitro* methods. To promote the principle of reduction, Member States should, where appropriate, facilitate the establishment of programmes for sharing the organs and tissue of animals that are killed.

(28) The welfare of the animals used in procedures is highly dependent on the quality and professional competence of the personnel supervising procedures, as well as of those performing procedures or supervising those taking care of the animals on a daily basis. Member States should ensure through authorisation or by other means that staff are adequately educated, trained and competent. Furthermore, it is important that staff are supervised until they have obtained and demonstrated the requisite competence. Non-binding guidelines at the level of the Union concerning educational requirements would, in the long run, promote the free movement of personnel.

(29) The establishments of breeders, suppliers and users should have adequate installations and equipment in place to meet the accommodation requirements of the animal species concerned and to allow the procedures to be performed efficiently and with the least distress to the animals. The breeders, suppliers and users should operate only if they are authorised by the competent authorities.

(30) To ensure the ongoing monitoring of animal-welfare needs, appropriate veterinary care should be available at all times and a staff member should be made responsible for the care and welfare of animals in each establishment.

(31) Animal-welfare considerations should be given the highest priority in the context of animal keeping, breeding and use. Breeders, suppliers and users should therefore have an animal-welfare body in place with the primary task of focusing on giving advice on animal-welfare issues. The body should also follow the development and outcome of projects at establishment level, foster a climate of care and provide tools for the practical application and timely implementation of recent technical and scientific developments in relation to the principles of replacement, reduction and refinement, in order to enhance the life-time experience of the animals. The advice given by the animal-welfare body should be properly documented and open to scrutiny during inspections.

(32) In order to enable competent authorities to monitor compliance with this Directive, each breeder, supplier and user should maintain accurate records of the numbers of animals, their origins and fate.

(33) Non-human primates, dogs and cats should have a personal history file from birth covering their lifetimes in order to be able to receive the care, accommodation and treatment that meet their individual needs and characteristics.

(34) The accommodation and care of animals should be based on the specific needs and characteristics of each species.

(35) There are differences in the requirements for the accommodation and care of animals between Member States, which contribute to the distortion of the internal market. Furthermore, some of those requirements no longer reflect the most recent knowledge on the impacts of accommodation and care conditions on both the animal welfare and the scientific results of procedures. It is therefore necessary to establish in this Directive harmonised requirements for accommodation and care. These requirements should be updated on the basis of scientific and technical development.

(36) To monitor compliance with this Directive, Member States should carry out regular inspections of breeders, suppliers and users on a risk basis. To ensure public confidence and promote transparency, an appropriate proportion of the inspections should be carried out without prior warning.

(37) To assist the Member States in the enforcement of this Directive and on the basis of the findings in the reports on the operation of the national inspections, the Commission should, when there is reason for concern, carry out controls of the national inspection systems. Member States should address any weaknesses identified in the findings of these controls.

(38) Comprehensive project evaluation, taking into account ethical considerations in the use of animals, forms the core of project authorisation and should ensure the implementation of principles of replacement, reduction and refinement in those projects.

(39) It is also essential, both on moral and scientific grounds, to ensure that each use of an animal is carefully evaluated as to the scientific or educational validity, usefulness and relevance of the expected result of that use. The likely harm to the animal should be balanced against the expected benefits of the project. Therefore, an impartial project evaluation independent of those involved in the study should be carried out as part of the authorisation process of projects involving the use of live animals. Effective implementation of a project evaluation should also allow for an appropriate assessment of the use of any new scientific experimental techniques as they emerge.

(40) Due to the nature of the project, the type of species used and the likelihood of achieving the desired objectives of the project, it might be necessary to carry out a retrospective assessment. Since projects may vary significantly in terms of complexity, length, and the time period for obtaining the results, it is necessary that the decision on retrospective assessment should be made taking those aspects fully into account.

(41) To ensure that the public is informed, it is important that objective information concerning projects using live animals is made publicly available. This should not violate proprietary rights or expose confidential information. Therefore, users should provide anonymous non-technical summaries of those projects, which Member States should publish. The published details should not breach the anonymity of the users.

(42) To manage risks to human and animal health and the environment, the legislation of the Union provides that substances and products can be marketed only after appropriate safety and efficacy data have been submitted. Some of those requirements can be fulfilled only by resorting to animal testing, hereinafter referred to as 'regulatory testing'. It is necessary to introduce specific measures in order to increase the use of alternative approaches and to eliminate unnecessary duplication of regulatory testing. For that purpose Member States should recognise the validity of test data produced using test methods provided for under the legislation of the Union.

(43) To reduce the administrative workload and enhance the competitiveness of research and industry in the Union, it should be possible to authorise multiple generic projects when carried out using established methods for testing, diagnostic or production purposes under one group authorisation, albeit without exempting any of these procedures from the project evaluation.

(44) To ensure effective examination of authorisation applications and to enhance the competitiveness of research and industry in the Union, a time-limit should be set for the competent authorities to evaluate project proposals and take decisions on the authorisation of such projects. In order not to compromise the quality of the project evaluation, additional time might be required for more complex project proposals due to the number of disciplines involved, the novel characteristics and more complex techniques of the proposed project. However, the extension of deadlines for project evaluation should remain the exception.

(45) Given the routine or repetitive nature of certain procedures, it is appropriate to provide for a regulatory option whereby the Member States could introduce a simplified administrative procedure for the evaluation of projects containing such procedures, provided certain requirements laid down in this Directive are complied with.

(46) The availability of alternative methods is highly dependent on the progress of the research into the development of alternatives. The Community Framework Programmes for Research and Technological Development provided increasing funding for projects which aim to replace, reduce and refine the use of animals in procedures. In order to increase competitiveness of research and industry in the Union and to replace, reduce and refine the use of animals in procedures, the Commission and the Member States should contribute through research and by other means to the development and validation of alternative approaches.

(47) The European Centre for the Validation of Alternative Methods, a policy action within the Joint Research Centre of the Commission, has coordinated the validation of alternative approaches in the Union since 1991. However, there is an increasing need for new methods to be developed and proposed for validation, which requires a reference laboratory of the Union for the validation of alternative methods to be established formally. This laboratory should be referred to as the European Centre for the Validation of Alternative Methods (ECVAM). It is necessary for the Commission to cooperate with the Member States when setting priorities for validation studies. The Member States should assist the Commission in identifying and nominating suitable laboratories to carry out such validation studies. For validation studies that are similar to previously validated methods and in respect of which a validation represents a significant competitive advantage, ECVAM should be able to collect charges from those who submit their methods for validation. Such charges should not be prohibitive of healthy competition in the testing industry.

(48) There is a need to ensure a coherent approach to project evaluation and review strategies at national level. Member States should establish national committees for the protection of animals used for scientific purposes to give advice to the competent authorities and animal-welfare bodies in order to promote the principles of replacement, reduction and refinement. A network of national committees should play a role in the exchange of best practice at the level of the Union.

(49) Technical and scientific advancements in biomedical research can be rapid, as can the increase in knowledge of factors influencing animal welfare. It is therefore necessary to provide for a review of this Directive. Such review should examine the possible replacement of the use of animals, and in particular non-human primates, as a matter of priority where it is possible, taking into account the advancement of science. The Commission should also conduct periodic thematic reviews concerning the replacement, reduction and refinement of the use of animals in procedures.

(50) In order to ensure uniform conditions for implementation, implementing powers should be conferred on the Commission to adopt guidelines at the level of the Union on the requirements with regard to education, training and competence of breeders', suppliers' and users' staff, to adopt detailed rules regarding the Union Reference Laboratory, its duties and tasks and the charges it may collect, to establish a common format for submitting the information by Member States to the Commission on the implementation of this Directive, statistical information and other specific information, and for the application of safeguard clauses. According to Article 291 TFEU, rules and general principles concerning mechanisms for the control by Member States of the Commission's exercise of implementing powers shall be laid down in advance by a regulation adopted in accordance with the ordinary legislative procedure. Pending the adoption of that new regulation, Council Decision 1999/468/EC of 28 June 1999 laying down the procedures for the exercise of implementing powers conferred on the Commission [1] continues to apply, with the exception of the regulatory procedure with scrutiny, which is not applicable.

(51) The Commission should be empowered to adopt delegated acts in accordance with Article 290 TFEU in respect of the following: modifications of the list of species falling under the obligation of being specifically bred for use in procedures; modifications of the care and accommodation standards; modifications of methods of killing, including their specifications; modifications of the elements to be used for the establishment by Member States of requirements with regard to education, training and competence of breeders', suppliers' and users' personnel; modifications of certain obligatory elements of the application for authorisation; modifications regarding the Union Reference Laboratory, its duties and tasks; as well as modifications of examples of different types of procedures assigned to each of the severity categories on the basis of factors related to the type of procedure. It is of particular importance that the Commission carry out appropriate consultation during its preparatory work, including at expert level.

(52) Member States should lay down rules on penalties applicable to infringements of the provisions of this Directive and ensure that they are implemented. Those penalties should be effective, proportionate and dissuasive.

(53) Directive 86/609/EEC should therefore be repealed. Certain modifications introduced by this Directive have a direct impact on the application of Regulation (EC) No 1069/2009 of the European Parliament and of the Council of 21 October 2009 laying down health rules as regards animal by-products and derived products not intended for human consumption [2]. It is therefore appropriate to amend a provision of that Regulation accordingly.

(54) Benefits to animal welfare from applying project authorisation retrospectively, and the related administrative costs, can only be justified for long term ongoing projects. Therefore, it is necessary to include transitional measures for ongoing short and medium term projects to avoid the need for retrospective authorisation with only limited benefits.

(55) In accordance with paragraph 34 of the Interinstitutional Agreement on better law-making, Member States are encouraged to draw up, for themselves and in the interests of the Union, their own tables, which will, as far as possible, illustrate the correlation between this Directive and the transposition measures, and to make them public.

(56) Since the objective of this Directive, namely the harmonisation of legislation concerning the use of animals for scientific purposes, cannot be sufficiently achieved by the Member States and can therefore, by reason of its scale and effects, be better achieved at Union level, the Union may adopt measures, in accordance with the principle of subsidiarity as set out in Article 5 of the Treaty on European Union. In accordance with the principle of proportionality, as set out in that Article, this Directive does not go beyond what is necessary in order to achieve that objective,

HAVE ADOPTED THIS DIRECTIVE:

CHAPTER I

GENERAL PROVISIONS

Article 1

Subject matter and scope

1. This Directive establishes measures for the protection of animals used for scientific or educational purposes.

To that end, it lays down rules on the following:

(a) the replacement and reduction of the use of animals in procedures and the refinement of the breeding, accommodation, care and use of animals in procedures;

(b) the origin, breeding, marking, care and accommodation and killing of animals;

(c) the operations of breeders, suppliers and users;

(d) the evaluation and authorisation of projects involving the use of animals in procedures.

2. This Directive shall apply where animals are used or intended to be used in procedures, or bred specifically so that their organs or tissues may be used for scientific purposes.

This Directive shall apply until the animals referred to in the first subparagraph have been killed, rehomed or returned to a suitable habitat or husbandry system.

[1] OJ L 184, 17.7.1999, p. 23.
[2] OJ L 300, 14.11.2009, p. 1.

The elimination of pain, suffering, distress or lasting harm by the successful use of anaesthesia, analgesia or other methods shall not exclude the use of an animal in procedures from the scope of this Directive.

3. This Directive shall apply to the following animals:

(a) live non-human vertebrate animals, including:

(i) independently feeding larval forms; and

(ii) foetal forms of mammals as from the last third of their normal development;

(b) live cephalopods.

4. This Directive shall apply to animals used in procedures, which are at an earlier stage of development than that referred to in point (a) of paragraph 3, if the animal is to be allowed to live beyond that stage of development and, as a result of the procedures performed, is likely to experience pain, suffering, distress or lasting harm after it has reached that stage of development.

5. This Directive shall not apply to the following:

(a) non-experimental agricultural practices;

(b) non-experimental clinical veterinary practices;

(c) veterinary clinical trials required for the marketing authorisation of a veterinary medicinal product;

(d) practices undertaken for the purposes of recognised animal husbandry;

(e) practices undertaken for the primary purpose of identification of an animal;

(f) practices not likely to cause pain, suffering, distress or lasting harm equivalent to, or higher than, that caused by the introduction of a needle in accordance with good veterinary practice.

6. This Directive shall apply without prejudice to Council Directive 76/768/EEC of 27 July 1976 on the approximation of the laws of the Member States relating to cosmetic products [1].

Article 2

Stricter national measures

1. Member States may, while observing the general rules laid down in the TFEU, maintain provisions in force on 9 November 2010, aimed at ensuring more extensive protection of animals falling within the scope of this Directive than those contained in this Directive.

Before 1 January 2013 Member States shall inform the Commission about such national provisions. The Commission shall bring them to the attention of other Member States.

2. When acting pursuant to paragraph 1, a Member State shall not prohibit or impede the supply or use of animals bred or kept in another Member State in accordance with this Directive, nor shall it prohibit or impede the placing on the market of products developed with the use of such animals in accordance with this Directive.

Article 3

Definitions

For the purposes of this Directive the following definitions shall apply:

1. 'procedure' means any use, invasive or non-invasive, of an animal for experimental or other scientific purposes, with known or unknown outcome, or educational purposes, which may cause the animal a level of pain, suffering, distress or lasting harm equivalent to, or higher than, that caused by the introduction of a needle in accordance with good veterinary practice.

   This includes any course of action intended, or liable, to result in the birth or hatching of an animal or the creation and maintenance of a genetically modified animal line in any such condition, but excludes the killing of animals solely for the use of their organs or tissues;

2. 'project' means a programme of work having a defined scientific objective and involving one or more procedures;

3. 'establishment' means any installation, building, group of buildings or other premises and may include a place that is not wholly enclosed or covered and mobile facilities;

4. 'breeder' means any natural or legal person breeding animals referred to in Annex I with a view to their use in procedures or for the use of their tissue or organs for scientific purposes, or breeding other animals primarily for those purposes, whether for profit or not;

5. 'supplier' means any natural or legal person, other than a breeder, supplying animals with a view to their use in procedures or for the use of their tissue or organs for scientific purposes, whether for profit or not;

6. 'user' means any natural or legal person using animals in procedures, whether for profit or not;

7. 'competent authority' means an authority or authorities or bodies designated by a Member State to carry out the obligations arising from this Directive.

Article 4

Principle of replacement, reduction and refinement

1. Member States shall ensure that, wherever possible, a scientifically satisfactory method or testing strategy, not entailing the use of live animals, shall be used instead of a procedure.

---

(1) OJ L 262, 27.9.1976, p. 169. Directive recast by Regulation (EC) No 1223/2009 of the European Parliament and the Council of 30 November 2009 on cosmetic products (OJ L 342, 22.12.2009, p. 59), which applies as from 11 July 2013.

2. Member States shall ensure that the number of animals used in projects is reduced to a minimum without compromising the objectives of the project.

3. Member States shall ensure refinement of breeding, accommodation and care, and of methods used in procedures, eliminating or reducing to the minimum any possible pain, suffering, distress or lasting harm to the animals.

4. This Article shall, in the choice of methods, be implemented in accordance with Article 13.

Article 5

Purposes of procedures

Procedures may be carried out for the following purposes only:

(a) basic research;

(b) translational or applied research with any of the following aims:

(i) the avoidance, prevention, diagnosis or treatment of disease, ill-health or other abnormality or their effects in human beings, animals or plants;

(ii) the assessment, detection, regulation or modification of physiological conditions in human beings, animals or plants; or

(iii) the welfare of animals and the improvement of the production conditions for animals reared for agricultural purposes;

(c) for any of the aims in point (b) in the development, manufacture or testing of the quality, effectiveness and safety of drugs, foodstuffs and feed-stuffs and other substances or products;

(d) protection of the natural environment in the interests of the health or welfare of human beings or animals;

(e) research aimed at preservation of the species;

(f) higher education, or training for the acquisition, maintenance or improvement of vocational skills;

(g) forensic inquiries.

Article 6

Methods of killing

1. Member States shall ensure that animals are killed with minimum pain, suffering and distress.

2. Member States shall ensure that animals are killed in the establishment of a breeder, supplier or user, by a competent person.

However, in the case of a field study an animal may be killed by a competent person outside of an establishment.

3. In relation to the animals covered by Annex IV, the appropriate method of killing as set out in that Annex shall be used.

4. Competent authorities may grant exemptions from the requirement in paragraph 3:

(a) to allow the use of another method provided that, on the basis of scientific evidence, the method is considered to be at least as humane; or

(b) when, on the basis of scientific justification, the purpose of the procedure cannot be achieved by the use of a method of killing set out in Annex IV.

5. Paragraphs 2 and 3 shall not apply where an animal has to be killed in emergency circumstances for animal-welfare, public-health, public-security, animal-health or environmental reasons.

CHAPTER II

PROVISIONS ON THE USE OF CERTAIN ANIMALS IN PROCEDURES

Article 7

Endangered species

1. Specimens of those endangered species listed in Annex A to Council Regulation (EC) No 338/97 of 9 December 1996 on the protection of species of wild fauna and flora by regulating trade therein [1], which do not fall within the scope of Article 7(1) of that Regulation, shall not be used in procedures, with the exception of those procedures meeting the following conditions:

(a) the procedure has one of the purposes referred to in points (b)(i), (c) or (e) of Article 5 of this Directive; and

(b) there is scientific justification to the effect that the purpose of the procedure cannot be achieved by the use of species other than those listed in that Annex.

2. Paragraph 1 shall not apply to any species of non-human primates.

Article 8

Non-human primates

1. Subject to paragraph 2, specimens of non-human primates shall not be used in procedures, with the exception of those procedures meeting the following conditions:

(a) the procedure has one of the purposes referred to in

(i) points (b)(i) or (c) of Article 5 of this Directive and is undertaken with a view to the avoidance, prevention, diagnosis or treatment of debilitating or potentially life-threatening clinical conditions in human beings; or

(ii) points (a) or (e) of Article 5;

and

(b) there is scientific justification to the effect that the purpose of the procedure cannot be achieved by the use of species other than non-human primates.

[1] OJ L 61, 3.3.1997, p. 1.

A debilitating clinical condition for the purposes of this Directive means a reduction in a person's normal physical or psychological ability to function.

2. Specimens of non-human primates listed in Annex A to Regulation (EC) No 338/97, which do not fall within the scope of Article 7(1) of that Regulation, shall not be used in procedures, with the exception of those procedures meeting the following conditions:

(a) the procedure has one of the purposes referred to in:

(i) points (b)(i) or (c) of Article 5 of this Directive and is undertaken with a view to the avoidance, prevention, diagnosis or treatment of debilitating or potentially life-threatening clinical conditions in human beings; or

(ii) Article 5(e);

and

(b) there is scientific justification to the effect that the purpose of the procedure cannot be achieved by the use of species other than non-human primates and by the use of species not listed in that Annex.

3. Notwithstanding paragraphs 1 and 2, great apes shall not be used in procedures, subject to the use of the safeguard clause in Article 55(2).

Article 9

Animals taken from the wild

1. Animals taken from the wild shall not be used in procedures.

2. Competent authorities may grant exemptions from paragraph 1 on the basis of scientific justification to the effect that the purpose of the procedure cannot be achieved by the use of an animal which has been bred for use in procedures.

3. The capture of animals in the wild shall be carried out only by competent persons using methods which do not cause the animals avoidable pain, suffering, distress or lasting harm.

Any animal found, at or after capture, to be injured or in poor health shall be examined by a veterinarian or another competent person and action shall be taken to minimise the suffering of the animal. Competent authorities may grant exemptions from the requirement of taking action to minimise the suffering of the animal if there is scientific justification.

Article 10

Animals bred for use in procedures

1. Member States shall ensure that animals belonging to the species listed in Annex I may only be used in procedures where those animals have been bred for use in procedures.

However, from the dates set out in Annex II, Member States shall ensure that non-human primates listed therein may be used in procedures only where they are the offspring of non-human primates which have been bred in captivity or where they are sourced from self-sustaining colonies.

For the purposes of this Article a 'self-sustaining colony' means a colony in which animals are bred only within the colony or sourced from other colonies but not taken from the wild, and where the animals are kept in a way that ensures that they are accustomed to humans.

The Commission shall, in consultation with the Member States and stakeholders, conduct a feasibility study, which shall include an animal health and welfare assessment, of the requirement laid down in the second subparagraph. The study shall be published no later than 10 November 2017. It shall be accompanied, where appropriate, by proposals for amendments to Annex II.

2. The Commission shall keep under review the use of sourcing non-human primates from self-sustaining colonies and, in consultation with the Member States and stakeholders, conduct a study to analyse the feasibility of sourcing animals only from self-sustaining colonies.

The study shall be published no later than 10 November 2022.

3. Competent authorities may grant exemptions from paragraph 1 on the basis of scientific justification.

Article 11

Stray and feral animals of domestic species

1. Stray and feral animals of domestic species shall not be used in procedures.

2. The competent authorities may only grant exemptions from paragraph 1 subject to the following conditions:

(a) there is an essential need for studies concerning the health and welfare of the animals or serious threats to the environment or to human or animal health; and

(b) there is scientific justification to the effect that the purpose of the procedure can be achieved only by the use of a stray or a feral animal.

CHAPTER III

PROCEDURES

Article 12

Procedures

1. Member States shall ensure that procedures are carried out in a user's establishment.

The competent authority may grant an exemption from the first subparagraph on the basis of scientific justification.

2. Procedures may be carried out only within the framework of a project.

Article 13

Choice of methods

1. Without prejudice to national legislation prohibiting certain types of methods, Member States shall ensure that a procedure is not carried out if another method or testing strategy for obtaining the result sought, not entailing the use of a live animal, is recognised under the legislation of the Union.

2. In choosing between procedures, those which to the greatest extent meet the following requirements shall be selected:

(a) use the minimum number of animals;

(b) involve animals with the lowest capacity to experience pain, suffering, distress or lasting harm;

(c) cause the least pain, suffering, distress or lasting harm;

and are most likely to provide satisfactory results.

3. Death as the end-point of a procedure shall be avoided as far as possible and replaced by early and humane end-points. Where death as the end-point is unavoidable, the procedure shall be designed so as to:

(a) result in the deaths of as few animals as possible; and

(b) reduce the duration and intensity of suffering to the animal to the minimum possible and, as far as possible, ensure a painless death.

Article 14

Anaesthesia

1. Member States shall ensure that, unless it is inappropriate, procedures are carried out under general or local anaesthesia, and that analgesia or another appropriate method is used to ensure that pain, suffering and distress are kept to a minimum.

Procedures that involve serious injuries that may cause severe pain shall not be carried out without anaesthesia.

2. When deciding on the appropriateness of using anaesthesia, the following shall be taken into account:

(a) whether anaesthesia is judged to be more traumatic to the animal than the procedure itself; and

(b) whether anaesthesia is incompatible with the purpose of the procedure.

3. Member States shall ensure that animals are not given any drug to stop or restrict their showing pain without an adequate level of anaesthesia or analgesia.

In these cases, a scientific justification shall be provided, accompanied by the details of the anaesthetic or analgesic regimen.

4. An animal, which may suffer pain once anaesthesia has worn off, shall be treated with pre-emptive and post-operative analgesics or other appropriate pain-relieving methods provided that it is compatible with the purpose of the procedure.

5. As soon as the purpose of the procedure has been achieved appropriate action shall be taken to minimise the suffering of the animal.

Article 15

Classification of severity of procedures

1. Member States shall ensure that all procedures are classified as 'non-recovery', 'mild', 'moderate', or 'severe' on a case-by-case basis using the assignment criteria set out in Annex VIII.

2. Subject to the use of the safeguard clause in Article 55(3), Member States shall ensure that a procedure is not performed if it involves severe pain, suffering or distress that is likely to be long-lasting and cannot be ameliorated.

Article 16

Reuse

1. Member States shall ensure that an animal already used in one or more procedures, when a different animal on which no procedure has previously been carried out could also be used, may only be reused in a new procedure provided that the following conditions are met:

(a) the actual severity of the previous procedures was 'mild' or 'moderate';

(b) it is demonstrated that the animal's general state of health and well-being has been fully restored;

(c) the further procedure is classified as 'mild', 'moderate' or 'non-recovery'; and

(d) it is in accordance with veterinary advice, taking into account the lifetime experience of the animal.

2. In exceptional circumstances, by way of derogation from point (a) of paragraph 1 and after a veterinary examination of the animal, the competent authority may allow reuse of an animal, provided the animal has not been used more than once in a procedure entailing severe pain, distress or equivalent suffering.

Article 17

End of the procedure

1. A procedure shall be deemed to end when no further observations are to be made for that procedure or, as regards new genetically modified animal lines, when the progeny are no longer observed or expected to experience pain, suffering, distress or lasting harm equivalent to, or higher than, that caused by the introduction of a needle.

2. At the end of a procedure, a decision to keep an animal alive shall be taken by a veterinarian or by another competent person. An animal shall be killed when it is likely to remain in moderate or severe pain, suffering, distress or lasting harm.

3. Where an animal is to be kept alive, it shall receive care and accommodation appropriate to its state of health.

Article 18

Sharing organs and tissues

Member States shall facilitate, where appropriate, the establishment of programmes for the sharing of organs and tissues of animals killed.

Article 19

Setting free of animals and rehoming

Member States may allow animals used or intended to be used in procedures to be rehomed, or returned to a suitable habitat or husbandry system appropriate to the species, provided that the following conditions are met:

(a) the state of health of the animal allows it;

(b) there is no danger to public health, animal health or the environment; and

(c) appropriate measures have been taken to safeguard the well-being of the animal.

CHAPTER IV

AUTHORISATION

Section 1

Requirements for breeders, suppliers and users

Article 20

Authorisation of breeders, suppliers and users

1. Member States shall ensure that all breeders, suppliers and users are authorised by, and registered with, the competent authority. Such authorisation may be granted for a limited period.

Authorisation shall be granted only if the breeder, supplier or user and its establishment is in compliance with the requirements of this Directive.

2. The authorisation shall specify the person responsible for ensuring compliance with the provisions of this Directive and the person or persons referred to in Article 24(1) and in Article 25.

3. Renewal of the authorisation shall be required for any significant change to the structure or the function of an establishment of a breeder, supplier or user that could negatively affect animal welfare.

4. Member States shall ensure that the competent authority is notified of any changes of the person or persons referred to in paragraph 2.

Article 21

Suspension and withdrawal of authorisation

1. Where a breeder, supplier or user no longer complies with the requirements set out in this Directive, the competent authority shall take appropriate remedial action, or require such action to be taken, or suspend or withdraw its authorisation.

2. Member States shall ensure that, where the authorisation is suspended or withdrawn, the welfare of the animals housed in the establishment is not adversely affected.

Article 22

Requirements for installations and equipment

1. Member States shall ensure that all establishments of a breeder, supplier or user have installations and equipment suited to the species of animals housed and, where procedures are carried out, to the performance of the procedures.

2. The design, construction and method of functioning of the installations and equipment referred to in paragraph 1 shall ensure that the procedures are carried out as effectively as possible, and aim at obtaining reliable results using the minimum number of animals and causing the minimum degree of pain, suffering, distress or lasting harm.

3. For the purposes of implementation of paragraphs 1 and 2, Member States shall ensure that the relevant requirements as set out in Annex III are complied with.

Article 23

Competence of personnel

1. Member States shall ensure that each breeder, supplier and user has sufficient staff on site.

2. The staff shall be adequately educated and trained before they perform any of the following functions:

(a) carrying out procedures on animals;

(b) designing procedures and projects;

(c) taking care of animals; or

(d) killing animals.

Persons carrying out the functions referred to in point (b) shall have received instruction in a scientific discipline relevant to the work being undertaken and shall have species-specific knowledge.

Staff carrying out functions referred to in points (a), (c) or (d) shall be supervised in the performance of their tasks until they have demonstrated the requisite competence.

Member States shall ensure, through authorisation or by other means, that the requirements laid down in this paragraph are fulfilled.

3. Member States shall publish, on the basis of the elements set out in Annex V, minimum requirements with regard to education and training and the requirements for obtaining, maintaining and demonstrating requisite competence for the functions set out in paragraph 2.

4. Non-binding guidelines at the level of the Union on the requirements laid down in paragraph 2 may be adopted in accordance with the advisory procedure referred to in Article 56(2).

Article 24

Specific requirements for personnel

1. Member States shall ensure that each breeder, supplier and user has one or several persons on site who shall:

(a) be responsible for overseeing the welfare and care of the animals in the establishment;

(b) ensure that the staff dealing with animals have access to information specific to the species housed in the establishment;

(c) be responsible for ensuring that the staff are adequately educated, competent and continuously trained and that they are supervised until they have demonstrated the requisite competence.

2. Member States shall ensure that persons specified in Article 40(2)(b) shall:

(a) ensure that any unnecessary pain, suffering, distress or lasting harm that is being inflicted on an animal in the course of a procedure is stopped; and

(b) ensure that the projects are carried out in accordance with the project authorisation or, in the cases referred to in Article 42, in accordance with the application sent to the competent authority or any decision taken by the competent authority, and ensure that in the event of non-compliance, the appropriate measures to rectify it are taken and recorded.

Article 25

Designated veterinarian

Member States shall ensure that each breeder, supplier and user has a designated veterinarian with expertise in laboratory animal medicine, or a suitably qualified expert where more appropriate, charged with advisory duties in relation to the well-being and treatment of the animals.

Article 26

Animal-welfare body

1. Member States shall ensure that each breeder, supplier and user sets up an animal-welfare body.

2. The animal-welfare body shall include at least the person or persons responsible for the welfare and care of the animals and, in the case of a user, a scientific member. The animal-welfare body shall also receive input from the designated veterinarian or the expert referred to in Article 25.

3. Member States may allow small breeders, suppliers and users to fulfil the tasks laid down in Article 27(1) by other means.

Article 27

Tasks of the animal-welfare body

1. The animal-welfare body shall, as a minimum, carry out the following tasks:

(a) advise the staff dealing with animals on matters related to the welfare of animals, in relation to their acquisition, accommodation, care and use;

(b) advise the staff on the application of the requirement of replacement, reduction and refinement, and keep it informed of technical and scientific developments concerning the application of that requirement;

(c) establish and review internal operational processes as regards monitoring, reporting and follow-up in relation to the welfare of animals housed or used in the establishment;

(d) follow the development and outcome of projects, taking into account the effect on the animals used, and identify and advise as regards elements that further contribute to replacement, reduction and refinement; and

(e) advise on rehoming schemes, including the appropriate socialisation of the animals to be rehomed.

2. Member States shall ensure that the records of any advice given by the animal-welfare body and decisions taken regarding that advice are kept for at least 3 years.

The records shall be made available to the competent authority upon request.

Article 28

Breeding strategy for non-human primates

Member States shall ensure that breeders of non-human primates have a strategy in place for increasing the proportion of animals that are the offspring of non-human primates that have been bred in captivity.

Article 29

Scheme for rehoming or setting free of animals

Where Member States allow rehoming, the breeders, suppliers and users from which animals are intended to be rehomed shall have a rehoming scheme in place that ensures socialisation of the animals that are rehomed. In the case of wild animals, where appropriate, a programme of rehabilitation shall be in place before they are returned to their habitat.

Article 30

Animal records

1. Member States shall ensure that all breeders, suppliers and users keep records of at least the following:

(a) the number and the species of animals bred, acquired, supplied, used in procedures, set-free or rehomed;

(b) the origin of the animals, including whether they are bred for use in procedures;

(c) the dates on which the animals are acquired, supplied, released or rehomed;

(d) from whom the animals are acquired;

(e) the name and address of the recipient of animals;

(f) the number and species of animals which died or were killed in each establishment. For animals that have died, the cause of death shall, when known, be noted; and

(g) in the case of users, the projects in which animals are used.

2. The records referred to in paragraph 1 shall be kept for a minimum of 5 years and made available to the competent authority upon request.

Article 31

Information on dogs, cats and non-human primates

1. Member States shall ensure that all breeders, suppliers and users keep the following information on each dog, cat and non-human primate:

(a) identity;

(b) place and date of birth, when available;

(c) whether it is bred for use in procedures; and

(d) in the case of a non-human primate, whether it is the offspring of non-human primates that have been bred in captivity.

2. Each dog, cat and non-human primate shall have an individual history file, which follows the animal as long as it is kept for the purposes of this Directive.

The file shall be established at birth or as soon as possible thereafter and shall cover any relevant reproductive, veterinary and social information on the individual animal and the projects in which it has been used.

3. The information referred to in this Article shall be kept for a minimum of 3 years after the death or rehoming of the animal and shall be made available to the competent authority upon request.

In the case of rehoming, relevant veterinary care and social information from the individual history file referred to in paragraph 2 shall accompany the animal.

Article 32

Marking and identification of dogs, cats and non-human primates

1. Each dog, cat or non-human primate shall be provided, at the latest at the time of weaning, with a permanent individual identification mark in the least painful manner possible.

2. Where a dog, cat or non-human primate is transferred from one breeder, supplier or user to another before it is weaned, and it is not practicable to mark it beforehand, a record, specifying in particular its mother, must be maintained by the receiver until it is marked.

3. Where an unmarked dog, cat or non-human primate, which is weaned, is received by a breeder, supplier or user it shall be permanently marked as soon as possible and in the least painful manner possible.

4. The breeder, supplier and user shall provide, at the request of the competent authority, reasons for which the animal is unmarked.

Article 33

Care and accommodation

1. Member States shall, as far as the care and accommodation of animals is concerned, ensure that:

(a) all animals are provided with accommodation, an environment, food, water and care which are appropriate to their health and well-being;

(b) any restrictions on the extent to which an animal can satisfy its physiological and ethological needs are kept to a minimum;

(c) the environmental conditions in which animals are bred, kept or used are checked daily;

(d) arrangements are made to ensure that any defect or avoidable pain, suffering, distress or lasting harm discovered is eliminated as quickly as possible; and

(e) animals are transported under appropriate conditions.

2. For the purposes of paragraph 1, Member States shall ensure that the care and accommodation standards set out in Annex III are applied from the dates provided for therein.

3. Member States may allow exemptions from the requirements of paragraph 1(a) or paragraph 2 for scientific, animal-welfare or animal-health reasons.

Section 2

Inspections

Article 34

Inspections by the Member States

1. Member States shall ensure that the competent authorities carry out regular inspections of all breeders, suppliers and users, including their establishments, to verify compliance with the requirements of this Directive.

2. The competent authority shall adapt the frequency of inspections on the basis of a risk analysis for each establishment, taking account of:

(a) the number and species of animals housed;

(b) the record of the breeder, supplier or user in complying with the requirements of this Directive;

(c) the number and types of projects carried out by the user in question; and

(d) any information that might indicate non-compliance.

3. Inspections shall be carried out on at least one third of the users each year in accordance with the risk analysis referred to in paragraph 2. However, breeders, suppliers and users of non-human primates shall be inspected at least once a year.

4. An appropriate proportion of the inspections shall be carried out without prior warning.

5. Records of all inspections shall be kept for at least 5 years.

Article 35

Controls of Member State inspections

1. The Commission shall, when there is due reason for concern, taking into account, inter alia, the proportion of inspections carried out without prior warning, undertake controls of the infrastructure and operation of national inspections in Member States.

2. The Member State in the territory of which the control referred to in paragraph 1 is being carried out shall give all necessary assistance to the experts of the Commission in carrying out their duties. The Commission shall inform the competent authority of the Member State concerned of the results of the control.

3. The competent authority of the Member State concerned shall take measures to take account of the results of the control referred to in paragraph 1.

Section 3

Requirements for projects

Article 36

Project authorisation

1. Member States shall ensure, without prejudice to Article 42, that projects are not carried out without prior authorisation from the competent authority, and that projects are carried out in accordance with the authorisation or, in the cases referred to in Article 42, in accordance with the application sent to the competent authority or any decision taken by the competent authority.

2. Member States shall ensure that no project is carried out unless a favourable project evaluation by the competent authority has been received in accordance with Article 38.

Article 37

Application for project authorisation

1. Member States shall ensure that an application for project authorisation is submitted by the user or the person responsible for the project. The application shall include at least the following:

(a) the project proposal;

(b) a non-technical project summary; and

(c) information on the elements set out in Annex VI.

2. Member States may waive the requirement in paragraph 1(b) for projects referred to in Article 42(1).

Article 38

Project evaluation

1. The project evaluation shall be performed with a degree of detail appropriate for the type of project and shall verify that the project meets the following criteria:

(a) the project is justified from a scientific or educational point of view or required by law;

(b) the purposes of the project justify the use of animals; and

(c) the project is designed so as to enable procedures to be carried out in the most humane and environmentally sensitive manner possible.

2. The project evaluation shall consist in particular of the following:

(a) an evaluation of the objectives of the project, the predicted scientific benefits or educational value;

(b) an assessment of the compliance of the project with the requirement of replacement, reduction and refinement;

(c) an assessment and assignment of the classification of the severity of procedures;

(d) a harm-benefit analysis of the project, to assess whether the harm to the animals in terms of suffering, pain and distress is justified by the expected outcome taking into account ethical considerations, and may ultimately benefit human beings, animals or the environment;

(e) an assessment of any justification referred to in Articles 6 to 12, 14, 16 and 33; and

(f) a determination as to whether and when the project should be assessed retrospectively.

3. The competent authority carrying out the project evaluation shall consider expertise in particular in the following areas:

(a) the areas of scientific use for which animals will be used including replacement, reduction and refinement in the respective areas;

(b) experimental design, including statistics where appropriate;

(c) veterinary practice in laboratory animal science or wildlife veterinary practice where appropriate;

(d) animal husbandry and care, in relation to the species that are intended to be used.

4. The project evaluation process shall be transparent.

Subject to safeguarding intellectual property and confidential information, the project evaluation shall be performed in an impartial manner and may integrate the opinion of independent parties.

Article 39

Retrospective assessment

1. Member States shall ensure that when determined in accordance with Article 38(2)(f), the retrospective assessment shall be carried out by the competent authority which shall, on the basis of the necessary documentation submitted by the user, evaluate the following:

(a) whether the objectives of the project were achieved;

(b) the harm inflicted on animals, including the numbers and species of animals used, and the severity of the procedures; and

(c) any elements that may contribute to the further implementation of the requirement of replacement, reduction and refinement.

2. All projects using non-human primates and projects involving procedures classified as 'severe', including those referred to in Article 15(2), shall undergo a retrospective assessment.

3. Without prejudice to paragraph 2 and by way of derogation from Article 38(2)(f), Member States may exempt projects involving only procedures classified as 'mild' or 'non-recovery' from the requirement for a retrospective assessment.

Article 40

Granting of project authorisation

1. The project authorisation shall be limited to procedures which have been subject to:

(a) a project evaluation; and

(b) the severity classifications assigned to those procedures.

2. The project authorisation shall specify the following:

(a) the user who undertakes the project;

(b) the persons responsible for the overall implementation of the project and its compliance with the project authorisation;

(c) the establishments in which the project will be undertaken, where applicable; and

(d) any specific conditions following the project evaluation, including whether and when the project shall be assessed retrospectively.

3. Project authorisations shall be granted for a period not exceeding 5 years.

4. Member States may allow the authorisation of multiple generic projects carried out by the same user if such projects are to satisfy regulatory requirements or if such projects use animals for production or diagnostic purposes with established methods.

Article 41

Authorisation decisions

1. Member States shall ensure that the decision regarding authorisation is taken and communicated to the applicant 40 working days at the latest from the receipt of the complete and correct application. This period shall include the project evaluation.

2. When justified by the complexity or the multi-disciplinary nature of the project, the competent authority may extend the period referred to in paragraph 1 once, by an additional period not exceeding 15 working days. The extension and its duration shall be duly motivated and shall be notified to the applicant before the expiry of the period referred to in paragraph 1.

3. Competent authorities shall acknowledge to the applicant all applications for authorisations as quickly as possible, and shall indicate the period referred to in paragraph 1 within which the decision is to be taken.

4. In the case of an incomplete or incorrect application, the competent authority shall, as quickly as possible, inform the applicant of the need to supply any additional documentation and of any possible effects on the running of the applicable time period.

Article 42

Simplified administrative procedure

1. Member States may decide to introduce a simplified administrative procedure for projects containing procedures classified as 'non-recovery', 'mild' or 'moderate' and not using non-human primates, that are necessary to satisfy regulatory requirements, or which use animals for production or diagnostic purposes with established methods.

2. When introducing a simplified administrative procedure, Member States shall ensure that the following provisions are met:

(a) the application specifies elements referred to in Article 40(2)(a), (b) and (c);

(b) a project evaluation is performed in accordance with Article 38; and

(c) that the period referred to in Article 41(1) is not exceeded.

3. If a project is changed in a way that may have a negative impact on animal welfare, Member States shall require an additional project evaluation with a favourable outcome.

4. Article 40(3) and (4), Article 41(3) and Article 44(3), (4) and (5) shall apply mutatis mutandis to projects that are allowed to be carried out in accordance with this Article.

Article 43

Non-technical project summaries

1. Subject to safeguarding intellectual property and confidential information, the non-technical project summary shall provide the following:

(a) information on the objectives of the project, including the predicted harm and benefits and the number and types of animals to be used;

(b) a demonstration of compliance with the requirement of replacement, reduction and refinement.

The non-technical project summary shall be anonymous and shall not contain the names and addresses of the user and its personnel.

2. Member States may require the non-technical project summary to specify whether a project is to undergo a retrospective assessment and by what deadline. In such a case, Member States shall ensure that the non-technical project summary is updated with the results of any retrospective assessment.

3. Member States shall publish the non-technical project summaries of authorised projects and any updates thereto.

Article 44

Amendment, renewal and withdrawal of a project authorisation

1. Member States shall ensure that amendment or renewal of the project authorisation is required for any change of the project that may have a negative impact on animal welfare.

2. Any amendment or renewal of a project authorisation shall be subject to a further favourable outcome of the project evaluation.

3. The competent authority may withdraw the project authorisation where the project is not carried out in accordance with the project authorisation.

4. Where a project authorisation is withdrawn, the welfare of the animals used or intended to be used in the project must not be adversely affected.

5. Member States shall establish and publish conditions for amendment and renewal of project authorisations.

Article 45

Documentation

1. Member States shall ensure that all relevant documentation, including project authorisations and the result of the project evaluation is kept for at least 3 years from the expiry date of the authorisation of the project or from the expiry of the period referred to in Article 41(1) and shall be available to the competent authority.

2. Without prejudice to paragraph 1, the documentation for projects which have to undergo retrospective assessment shall be kept until the retrospective assessment has been completed.

CHAPTER V

AVOIDANCE OF DUPLICATION AND ALTERNATIVE APPROACHES

Article 46

Avoidance of duplication of procedures

Each Member State shall accept data from other Member States that are generated by procedures recognised by the legislation of the Union, unless further procedures need to be carried out regarding that data for the protection of public health, safety or the environment.

Article 47

Alternative approaches

1. The Commission and the Member States shall contribute to the development and validation of alternative approaches which could provide the same or higher levels of information as those obtained in procedures using animals, but which do not involve the use of animals or use fewer animals or which entail less painful procedures, and they shall take such other steps as they consider appropriate to encourage research in this field.

2. Member States shall assist the Commission in identifying and nominating suitable specialised and qualified laboratories to carry out such validation studies.

3. After consulting the Member States, the Commission shall set the priorities for those validation studies and allocate the tasks between the laboratories for carrying out those studies.

4. Member States shall, at national level, ensure the promotion of alternative approaches and the dissemination of information thereon.

5. Member States shall nominate a single point of contact to provide advice on the regulatory relevance and suitability of alternative approaches proposed for validation.

6. The Commission shall take appropriate action with a view to obtaining international acceptance of alternative approaches validated in the Union.

Article 48

Union Reference Laboratory

1. The Union Reference Laboratory and its duties and tasks shall be those referred to in Annex VII.

2. The Union Reference Laboratory may collect charges for the services it provides that do not directly contribute to the further advancement of replacement, reduction and refinement.

3. Detailed rules necessary for the implementation of paragraph 2 of this Article and Annex VII may be adopted in accordance with the regulatory procedure referred to in Article 56(3).

Article 49

National committees for the protection of animals used for scientific purposes

1. Each Member State shall establish a national committee for the protection of animals used for scientific purposes. It shall advise the competent authorities and animal-welfare bodies on matters dealing with the acquisition, breeding, accommodation, care and use of animals in procedures and ensure sharing of best practice.

2. The national committees referred to in paragraph 1 shall exchange information on the operation of animal-welfare bodies and project evaluation and share best practice within the Union.

CHAPTER VI

FINAL PROVISIONS

Article 50

Adaptation of Annexes to technical progress

In order to ensure that the provisions of Annexes I and III to VIII reflect the state of technical or scientific progress, taking into account the experience gained in the implementation of this Directive, in particular through the reporting referred to in Article 54(1), the Commission may adopt, by means of delegated acts in accordance with Article 51 and subject to the conditions laid down in Articles 52 and 53, modifications of those Annexes, with the exception of provisions of Sections I and II of Annex VIII. The dates referred to in Section B of Annex III shall not be brought forward. When adopting such delegated acts, the Commission shall act in accordance with the relevant provisions of this Directive.

Article 51

Exercise of the delegation

1. The power to adopt delegated acts referred to in Article 50 shall be conferred on the Commission for a period of 8 years beginning on 9 November 2010. The Commission shall make a report in respect of the delegated power at the latest 12 months before the end of the 8-year period. The delegation of power shall be automatically extended for periods of an identical duration, unless the European Parliament or the Council revokes it in accordance with Article 52.

2. As soon as it adopts a delegated act, the Commission shall notify it simultaneously to the European Parliament and to the Council.

3. The power to adopt delegated acts is conferred on the Commission subject to the conditions laid down in Articles 52 and 53.

Article 52

Revocation of the delegation

1. The delegation of power referred to in Article 50 may be revoked at any time by the European Parliament or by the Council.

2. The institution which has commenced an internal procedure for deciding whether to revoke the delegation of power shall endeavour to inform the other institution and the Commission within a reasonable time before the final decision is taken, indicating the delegated power which could be subject to revocation and possible reasons for a revocation.

3. The decision of revocation shall put an end to the delegation of the power specified in that decision. It shall take effect immediately or at a later date specified therein. It shall not affect the validity of the delegated acts already in force. It shall be published in the *Official Journal of the European Union*.

Article 53

Objections to delegated acts

1. The European Parliament or the Council may object to a delegated act within a period of 2 months from the date of notification.

At the initiative of the European Parliament or the Council this period shall be extended by 2 months.

2. If, on expiry of that period, neither the European Parliament nor the Council has objected to the delegated act, it shall be published in the *Official Journal of the European Union* and shall enter into force at the date stated therein.

The delegated act may be published in the *Official Journal of the European Union* and enter into force before the expiry of that period if the European Parliament and the Council have both informed the Commission of their intention not to raise objections.

3. If the European Parliament or the Council objects to a delegated act, it shall not enter into force. The institution which objects shall state the reasons for objecting to the delegated act.

Article 54

Reporting

1. Member States shall by 10 November 2018, and every 5 years thereafter, send the information on the implementation of this Directive and in particular Articles 10(1), 26, 28, 34, 38, 39, 43 and 46 to the Commission.

2. Member States shall collect and make publicly available, on an annual basis, statistical information on the use of animals in procedures, including information on the actual severity of the procedures and on the origin and species of non-human primates used in procedures.

Member States shall submit that statistical information to the Commission by 10 November 2015 and every year thereafter.

3. Member States shall submit to the Commission, on annual basis, detailed information on exemptions granted under Article 6(4)(a).

4. The Commission shall by 10 May 2012 establish a common format for submitting the information referred to in paragraphs 1, 2, and 3 of this Article in accordance with the regulatory procedure referred to in Article 56(3).

Article 55

Safeguard clauses

1. Where a Member State has scientifically justifiable grounds for believing it is essential to use non-human primates for the purposes referred to in Article 8(1)(a)(i) with regard to human beings, but where the use is not undertaken with a view to the avoidance, prevention, diagnosis or treatment of debilitating or potentially life-threatening clinical conditions, it may adopt a provisional measure allowing such use, provided the purpose cannot be achieved by the use of species other than non-human primates.

2. Where a Member State has justifiable grounds for believing that action is essential for the preservation of the species or in relation to an unexpected outbreak of a life-threatening or debilitating clinical condition in human beings, it may adopt a provisional measure allowing the use of great apes in procedures having one of the purposes referred to in points (b)(i), (c) or (e) of Article 5; provided that the purpose of the procedure cannot be achieved by the use of species other than great apes or by the use of alternative methods. However, the reference to Article 5(b)(i) shall not be taken to include the reference to animals and plants.

3. Where, for exceptional and scientifically justifiable reasons, a Member State deems it necessary to allow the use of a procedure involving severe pain, suffering or distress that is likely to be long-lasting and cannot be ameliorated, as referred to in Article 15(2), it may adopt a provisional measure to allow such procedure. Member States may decide not to allow the use of non-human primates in such procedures.

4. A Member State which has adopted a provisional measure in accordance with paragraph 1, 2 or 3 shall immediately inform the Commission and the other Member States thereof, giving reasons for its decision and submitting evidence of the situation as described in paragraphs 1, 2 and 3 on which the provisional measure is based.

The Commission shall put the matter before the Committee referred to in Article 56(1) within 30 days of receipt of the information from the Member State and shall, in accordance with the regulatory procedure referred to in Article 56(3), either:

(a) authorise the provisional measure for a time period defined in the decision; or

(b) require the Member State to revoke the provisional measure.

Article 56

Committee

1. The Commission shall be assisted by a Committee.

2. Where reference is made to this paragraph, Articles 3 and 7 of Decision 1999/468/EC shall apply, having regard to the provisions of Article 8 thereof.

3. Where reference is made to this paragraph, Articles 5 and 7 of Decision 1999/468/EC shall apply, having regard to the provisions of Article 8 thereof.

The period laid down in Article 5(6) of Decision 1999/468/EC shall be set at 3 months.

Article 57

Commission report

1. By 10 November 2019 and every 5 years thereafter, the Commission shall, based on the information received from the Member States under Article 54(1), submit to the European Parliament and the Council a report on the implementation of this Directive.

2. By 10 November 2019 and every 3 years thereafter, the Commission shall, based on the statistical information submitted by Member States under Article 54(2), submit to the European Parliament and the Council a summary report on that information.

Article 58

Review

The Commission shall review this Directive by 10 November 2017, taking into account advancements in the development of alternative methods not entailing the use of animals, in particular of non-human primates, and shall propose amendments, where appropriate.

The Commission shall, where appropriate, and in consultation with the Member States and stakeholders, conduct periodic thematic reviews of the replacement, reduction and refinement of the use of animals in procedures, paying specific attention to non-human primates, technological developments, and new scientific and animal-welfare knowledge.

Article 59

Competent authorities

1. Each Member State shall designate one or more competent authorities responsible for the implementation of this Directive.

Member States may designate bodies other than public authorities for the implementation of specific tasks laid down in this Directive only if there is proof that the body:

(a) has the expertise and infrastructure required to carry out the tasks; and

(b) is free of any conflict of interests as regards the performance of the tasks.

Bodies thus designated shall be considered competent authorities for the purposes of this Directive.

2. Each Member State shall communicate details of a national authority serving as contact point for the purposes of this Directive to the Commission by 10 February 2011, as well as any update to such data.

The Commission shall make publicly available the list of those contact points.

Article 60

Penalties

Member States shall lay down the rules on penalties applicable to infringements of the national provisions adopted pursuant to this Directive and shall take all measures necessary to ensure that they are implemented. The penalties provided for must be effective, proportionate and dissuasive. The Member States shall notify those provisions to the Commission by 10 February 2013, and shall notify the Commission without delay of any subsequent amendment affecting them.

Article 61

Transposition

1. Member States shall adopt and publish, by 10 November 2012, the laws, regulations and administrative provisions necessary to comply with this Directive. They shall forthwith communicate to the Commission the text of those provisions.

They shall apply those provisions from 1 January 2013.

When Member States adopt those provisions, they shall contain a reference to this Directive or be accompanied by such reference on the occasion of their official publication. The method of making such reference shall be laid down by Member States.

2. Member States shall communicate to the Commission the text of the main provisions of national law which they adopt in the field covered by this Directive.

Article 62

Repeal

1. Directive 86/609/EEC is repealed with effect from 1 January 2013 with the exception of Article 13, which shall be repealed with effect from 10 May 2013.

2. References to the repealed Directive shall be construed as references to this Directive.

Article 63

Amendment of Regulation (EC) No 1069/2009

Point (a)(iv) of Article 8 of Regulation (EC) No 1069/2009 is replaced by the following:

‘(iv) animals used in a procedure or procedures defined in Article 3 of Directive 2010/63/EU of the European Parliament and of the Council of 22 September 2010 on the protection of animals used for scientific purposes (*), in cases where the competent authority decides that such animals or any of their body parts have the potential to pose serious health risks to humans or to other animals, as a result of that procedure or those procedures without prejudice to Article 3(2) of Regulation (EC) No 1831/2003;

(*) OJ L 276, 20.10.2010, p. 33’.

Article 64

Transitional provisions

1. Member States shall not apply laws, regulations and administrative provisions adopted in accordance with Articles 36 to 45 to projects which have been approved before 1 January 2013 and the duration of which does not extend beyond 1 January 2018.

2. Projects which have been approved before 1 January 2013 and the duration of which extends beyond 1 January 2018 shall obtain project authorisation by 1 January 2018.

Article 65

Entry into force

This Directive shall enter into force on the 20th day following its publication in the *Official Journal of the European Union*.

Article 66

Addressees

This Directive is addressed to the Member States.

Done at Strasbourg, 22 September 2010.

For the European Parliament
The President
J. BUZEK

For the Council
The President
O. CHASTEL

ANNEX I

LIST OF ANIMALS REFERRED TO IN ARTICLE 10

1. Mouse (*Mus musculus*)
2. Rat (*Rattus norvegicus*)
3. Guinea pig (*Cavia porcellus*)
4. Syrian (golden) hamster (*Mesocricetus auratus*)
5. Chinese hamster (*Cricetulus griseus*)
6. Mongolian gerbil (*Meriones unguiculatus*)
7. Rabbit (*Oryctolagus cuniculus*)
8. Dog (*Canis familiaris*)
9. Cat (*Felis catus*)
10. All species of non-human primates
11. Frog (*Xenopus* (*laevis, tropicalis*), Rana (*temporaria, pipiens*))
12. Zebra fish (*Danio rerio*)

ANNEX II

LIST OF NON-HUMAN PRIMATES AND DATES REFERRED TO IN THE SECOND SUBPARAGRAPH OF ARTICLE 10(1)

Species	Dates
Marmoset (*Callithrix jacchus*)	1 January 2013
Cynomolgus monkey (*Macaca fascicularis*)	5 years after the publication of the feasibility study referred to in Article 10(1), fourth subparagraph, provided the study does not recommend an extended period
Rhesus monkey (*Macaca mulatta*)	5 years after the publication of the feasibility study referred to in Article 10(1), fourth subparagraph, provided the study does not recommend an extended period
Other species of non-human primates	5 years after the publication of the feasibility study referred to in Article 10(1), fourth subparagraph, provided the study does not recommend an extended period

ANNEX III

REQUIREMENTS FOR ESTABLISHMENTS AND FOR THE CARE AND ACCOMMODATION OF ANIMALS

Section A: General section

1. The physical facilities

1.1. Functions and general design

(a) All facilities shall be constructed so as to provide an environment which takes into account the physiological and ethological needs of the species kept in them. Facilities shall also be designed and managed to prevent access by unauthorised persons and the ingress or escape of animals.

(b) Establishments shall have an active maintenance programme to prevent and remedy any defect in buildings or equipment.

1.2. Holding rooms

(a) Establishments shall have a regular and efficient cleaning schedule for the rooms and shall maintain satisfactory hygienic standards.

(b) Walls and floors shall be surfaced with a material resistant to the heavy wear and tear caused by the animals and the cleaning process. The material shall not be detrimental to the health of the animals and shall be such that the animals cannot hurt themselves. Additional protection shall be given to any equipment or fixtures so that they are not damaged by the animals nor do they cause injury to the animals themselves.

(c) Species that are incompatible, for example predator and prey, or animals requiring different environmental conditions, shall not be housed in the same room nor, in the case of predator and prey, within sight, smell or sound of each other.

1.3. General and special purpose procedure rooms

(a) Establishments shall, where appropriate, have available laboratory facilities for the carrying out of simple diagnostic tests, post-mortem examinations, and/or the collection of samples that are to be subjected to more extensive laboratory investigations elsewhere. General and special purpose procedure rooms shall be available for situations where it is undesirable to carry out the procedures or observations in the holding rooms.

(b) Facilities shall be provided to enable newly-acquired animals to be isolated until their health status can be determined and the potential health risk to established animals assessed and minimised.

(c) There shall be accommodation for the separate housing of sick or injured animals.

1.4. Service rooms

(a) Store-rooms shall be designed, used and maintained to safeguard the quality of food and bedding. These rooms shall be vermin and insect-proof, as far as possible. Other materials, which may be contaminated or present a hazard to animals or staff, shall be stored separately.

(b) The cleaning and washing areas shall be large enough to accommodate the installations necessary to decontaminate and clean used equipment. The cleaning process shall be arranged so as to separate the flow of clean and dirty equipment to prevent the contamination of newly-cleaned equipment.

(c) Establishments shall provide for the hygienic storage and safe disposal of carcasses and animal waste.

(d) Where surgical procedures under aseptic conditions are required there shall be provision for one or more than one suitably equipped room, and facilities provided for postoperative recovery.

2. The environment and control thereof

2.1. Ventilation and temperature

(a) Insulation, heating and ventilation of the holding room shall ensure that the air circulation, dust levels, and gas concentrations are kept within limits that are not harmful to the animals housed.

(b) Temperature and relative humidity in the holding rooms shall be adapted to the species and age groups housed. The temperature shall be measured and logged on a daily basis.

(c) Animals shall not be restricted to outdoor areas under climatic conditions which may cause them distress.

2.2. Lighting

(a) Where natural light does not provide an appropriate light/dark cycle, controlled lighting shall be provided to satisfy the biological requirements of the animals and to provide a satisfactory working environment.

(b) Illumination shall satisfy the needs for the performance of husbandry procedures and inspection of the animals.

(c) Regular photoperiods and intensity of light adapted to the species shall be provided.

(d) When keeping albino animals, the lighting shall be adjusted to take into account their sensitivity to light.

2.3. Noise

(a) Noise levels including ultrasound, shall not adversely affect animal welfare.

(b) Establishments shall have alarm systems that sound outside the sensitive hearing range of the animals, where this does not conflict with their audibility to human beings.

(c) Holding rooms shall where appropriate be provided with noise insulation and absorption materials.

2.4. Alarm systems

(a) Establishments relying on electrical or mechanical equipment for environmental control and protection, shall have a stand-by system to maintain essential services and emergency lighting systems as well as to ensure that alarm systems themselves do not fail to operate.

(b) Heating and ventilation systems shall be equipped with monitoring devices and alarms.

(c) Clear instructions on emergency procedures shall be prominently displayed.

3. Care of animals

3.1. Health

(a) Establishments shall have a strategy in place to ensure that a health status of the animals is maintained that safeguards animal welfare and meets scientific requirements. This strategy shall include regular health monitoring, a microbiological surveillance programme and plans for dealing with health breakdowns and shall define health parameters and procedures for the introduction of new animals.

(b) Animals shall be checked at least daily by a competent person. These checks shall ensure that all sick or injured animals are identified and appropriate action is taken.

3.2. Animals taken from the wild

(a) Transport containers and means of transport adapted to the species concerned shall be available at capture sites, in case animals need to be moved for examination or treatment.

(b) Special consideration shall be given and appropriate measures taken for the acclimatisation, quarantine, housing, husbandry, care of animals taken from the wild and, as appropriate, provisions for setting them free at the end of procedures.

3.3. Housing and enrichment

(a) Housing

Animals, except those which are naturally solitary, shall be socially housed in stable groups of compatible individuals. In cases where single housing is allowed in accordance with article 33(3) the duration shall be limited to the minimum period necessary and visual, auditory, olfactory and/or tactile contact shall be maintained. The introduction or re-introduction of animals to established groups shall be carefully monitored to avoid problems of incompatibility and disrupted social relationships.

(b) Enrichment

All animals shall be provided with space of sufficient complexity to allow expression of a wide range of normal behaviour. They shall be given a degree of control and choice over their environment to reduce stress-induced behaviour. Establishments shall have appropriate enrichment techniques in place, to extend the range of activities available to the animals and increase their coping activities including physical exercise, foraging, manipulative and cognitive activities, as appropriate to the species. Environmental enrichment in animal enclosures shall be adapted to the species and individual needs of the animals concerned. The enrichment strategies in establishments shall be regularly reviewed and updated.

(c) Animal enclosures

Animal enclosures shall not be made out of materials detrimental to the health of the animals. Their design and construction shall be such that no injury to the animals is caused. Unless they are disposable, they shall be made from materials that will withstand cleaning and decontamination techniques. The design of animal enclosure floors shall be adapted to the species and age of the animals and be designed to facilitate the removal of excreta.

3.4. Feeding

(a) The form, content and presentation of the diet shall meet the nutritional and behavioural needs of the animal.

(b) The animals' diet shall be palatable and non-contaminated. In the selection of raw materials, production, preparation and presentation of feed, establishments shall take measures to minimise chemical, physical and microbiological contamination.

(c) Packing, transport and storage shall be such as to avoid contamination, deterioration or destruction. All feed hoppers, troughs or other utensils used for feeding shall be regularly cleaned and, if necessary, sterilised.

(d) Each animal shall be able to access the food, with sufficient feeding space provided to limit competition.

3.5. Watering

(a) Uncontaminated drinking water shall always be available to all animals.

(b) When automatic watering systems are used, they shall be regularly checked, serviced and flushed to avoid accidents. If solid-bottomed cages are used, care shall be taken to minimise the risk of flooding.

(c) Provision shall be made to adapt the water supply for aquaria and tanks to the needs and tolerance limits of the individual fish, amphibian and reptile species.

3.6. Resting and sleeping areas

(a) Bedding materials or sleeping structures adapted to the species shall always be provided, including nesting materials or structures for breeding animals.

(b) Within the animal enclosure, as appropriate to the species, a solid, comfortable resting area for all animals shall be provided. All sleeping areas shall be kept clean and dry.

3.7. Handling

Establishments shall set up habituation and training programmes suitable for the animals, the procedures and length of the project.

Section B: Species-specific section

1. Mice, rats, gerbils, hamsters and guinea pigs

In this and subsequent tables for mice, rats, gerbils, hamsters and guinea pigs, 'enclosure height' means the vertical distance between the enclosure floor and the top of the enclosure and this height applies over more than 50 % of the minimum enclosure floor area prior to the addition of enrichment devices.

When designing procedures, consideration shall be given to the potential growth of the animals to ensure adequate space is provided (as detailed in Tables 1.1 to 1.5) for the duration of the study.

Table 1.1.

Mice

	Body weight (g)	Minimum enclosure size (cm^2)	Floor area per animal (cm^2)	Minimum enclosure height (cm)	Date referred to in Article 33(2)
In stock and during procedures	up to 20	330	60	12	1 January 2017
	over 20 to 25	330	70	12	
	over 25 to 30	330	80	12	
	over 30	330	100	12	
Breeding		330<br>For a monogamous pair (outbred/inbred) or a trio (inbred). For each additional female plus litter 180 cm^2 shall be added.		12	
Stock at breeders (*)<br>Enclosure size<br>950 cm^2	less than 20	950	40	12	
Enclosure size<br>1 500 cm^2	less than 20	1 500	30	12	

(*) Post-weaned mice may be kept at these higher stocking densities for the short period after weaning until issue, provided that the animals are housed in larger enclosures with adequate enrichment, and these housing conditions do not cause any welfare deficit such as increased levels of aggression, morbidity or mortality, stereotypes and other behavioural deficits, weight loss, or other physiological or behavioural stress responses.

Table 1.2.

Rats

	Body weight (g)	Minimum enclosure size (cm^2)	Floor area per animal (cm^2)	Minimum enclosure height (cm)	Date referred to in Article 33(2)
In stock and during procedures (*)	up to 200	800	200	18	1 January 2017
	over 200 to 300	800	250	18	
	over 300 to 400	800	350	18	
	over 400 to 600	800	450	18	
	over 600	1 500	600	18	

	Body weight (g)	Minimum enclosure size (cm^2)	Floor area per animal (cm^2)	Minimum enclosure height (cm)	Date referred to in Article 33(2)
Breeding		800<br>Mother and litter. For each additional adult animal permanently added to the enclosure add 400 cm^2		18	
Stock at breeders (**)<br>Enclosure size 1 500 cm^2	up to 50	1 500	100	18	
	over 50 to 100	1 500	125	18	
	over 100 to 150	1 500	150	18	
	over 150 to 200	1 500	175	18	
Stock at breeders (**)<br>Enclosure size 2 500 cm^2	up to 100	2 500	100	18	
	over 100 to 150	2 500	125	18	
	over 150 to 200	2 500	150	18	

(*) In long-term studies, if space allowances per individual animal fall below those indicated above towards the end of such studies, priority shall be given to maintaining stable social structures.

(**) Post-weaned rats may be kept at these higher stocking densities for the short period after weaning until issue, provided that the animals are housed in larger enclosures with adequate enrichment, and these housing conditions do not cause any welfare deficit such as increased levels of aggression, morbidity or mortality, stereotypes and other behavioural deficits, weight loss, or other physiological or behavioural stress responses.

Table 1.3.

Gerbils

	Body weight (g)	Minimum enclosure size (cm^2)	Floor area per animal (cm^2)	Minimum enclosure height (cm)	Date referred to in Article 33(2)
In stock and during procedures	up to 40	1 200	150	18	1 January 2017
	over 40	1 200	250	18	
Breeding		1 200<br>Monogamous pair or trio with offspring		18	

Table 1.4.

Hamsters

	Body weight (g)	Minimum enclosure size (cm^2)	Floor area per animal (cm^2)	Minimum enclosure height (cm)	Date referred to in Article 33(2)
In stock and during procedures	up to 60	800	150	14	1 January 2017
	over 60 to 100	800	200	14	
	over 100	800	250	14	

	Body weight (g)	Minimum enclosure size (cm^2)	Floor area per animal (cm^2)	Minimum enclosure height (cm)	Date referred to in Article 33(2)
Breeding		800<br>Mother or monogamous pair with litter		14	
Stock at breeders (*)	less than 60	1 500	100	14	

(*) Post-weaned hamsters may be kept at these higher stocking densities, for the short period after weaning until issue provided that the animals are housed in larger enclosures with adequate enrichment, and these housing conditions do not cause any welfare deficit such as increased levels of aggression, morbidity or mortality, stereotypes and other behavioural deficits, weight loss, or other physiological or behavioural stress responses.

Table 1.5.

Guinea pigs

	Body weight (g)	Minimum enclosure size (cm^2)	Floor area per animal (cm^2)	Minimumen-closure height (cm)	Date referred to in Article 33(2)
In stock and during procedures	up to 200	1 800	200	23	1 January 2017
	over 200 to 300	1 800	350	23	
	over 300 to 450	1 800	500	23	
	over 450 to 700	2 500	700	23	
	over 700	2 500	900	23	
Breeding		2 500<br>Pair with litter. For each additional breeding female add 1 000 cm^2		23	

2. Rabbits

During agricultural research, when the aim of the project requires that the animals are kept under similar conditions to those under which commercial farm animals are kept, the keeping of the animals shall at least follow the standards laid down in Directive 98/58/EC (1).

A raised area shall be provided within the enclosure. This raised area must allow the animal to lie and sit and easily move underneath, and shall not cover more than 40 % of the floor space. When for scientific or veterinary reasons a raised area cannot be used, the enclosure shall be 33 % larger for a single rabbit and 60 % larger for two rabbits. Where a raised area is provided for rabbits of less than 10 weeks of age, the size of the raised area shall be at least of 55 cm by 25 cm and the height above the floor shall be such that the animals can make use of it.

Table 2.1.

Rabbits over 10 weeks of age

Table 2.1 is to be used for both cages and pens. The additional floor area is as a minimum 3 000 cm^2 per rabbit for the third, the fourth, the fifth and the sixth rabbit, while 2 500 cm^2 as a minimum shall be added for each additional rabbit above a number of six.

(1) Council Directive 98/58/EC of 20 July 1998 concerning the protection of animals kept for farming purposes (OJ L 221, 8.8.1998, p. 23).

Final body weight (kg)	Minimum floor area for one or two socially harmonious animals (cm^2)	Minimum height (cm)	Date referred to in Article 33(2)
less than 3	3 500	45	1 January 2017
from 3 to 5	4 200	45	
over 5	5 400	60	

Table 2.2.

Doe plus litter

Doe weight (kg)	Minimum enclosure size (cm^2)	Addition for nest boxes (cm^2)	Minimum height (cm)	Date referred to in Article 33(2)
less than 3	3 500	1 000	45	1 January 2017
from 3 to 5	4 200	1 200	45	
over 5	5 400	1 400	60	

Table 2.3.

Rabbits less than 10 weeks of age

Table 2.3 is to be used for both cages and pens.

Age	Minimum enclosure size (cm^2)	Minimum floor area per animal (cm^2)	Minimum height (cm)	Date referred to in Article 33(2)
Weaning to 7 weeks	4 000	800	40	1 January 2017
From 7 to 10 weeks	4 000	1 200	40	

Table 2.4.

Rabbits: Optimal dimensions for raised areas for enclosures having the dimensions indicated in Table 2.1.

Age in weeks	Final body weight (kg)	Optimum size (cm x cm)	Optimum height from the enclosure floor (cm)	Date referred to in Article 33(2)
over 10	less than 3	55 × 25	25	1 January 2017
	from 3 to 5	55 × 30	25	
	over 5	60 × 35	30	

3. Cats

Cats shall not be single-housed for more than 24 hours at a time. Cats that are repeatedly aggressive towards other cats shall be housed singly only if a compatible companion cannot be found. Social stress in all pair- or group-housed individuals shall be monitored at least weekly. Females with kittens under four weeks of age or in the last two weeks of pregnancy may be housed singly.

Table 3.

Cats

The minimum space in which a queen and litter may be held is the space for a single cat, which shall be gradually increased so that by 4 months of age litters have been rehoused following the space requirements for adults.

Areas for feeding and for litter trays shall not be less than 0,5 metres apart and shall not be interchanged.

	Floor (*) (m^2)	Shelves (m^2)	Height (m)	Date referred to in Article 33(2)
Minimum for one adult animal	1,5	0,5	2	1 January 2017
For each additional animal add	0,75	0,25	—	

(*) Floor area excluding shelves.

4. Dogs

Dogs shall where possible be provided with outside runs. Dogs shall not be single-housed for more than 4 hours at a time.

The internal enclosure shall represent at least 50 % of the minimum space to be made available to the dogs, as detailed in Table 4.1.

The space allowances detailed below are based on the requirements of beagles, but giant breeds such as St Bernards or Irish wolfhounds shall be provided with allowances significantly in excess of those detailed in Table 4.1. For breeds other than the laboratory beagle, space allowances shall be determined in consultation with veterinary staff.

Table 4.1.

Dogs

Dogs that are pair or group housed may each be constrained to half the total space provided (2 m^2 for a dog under 20 kg, 4 m^2 for a dog over 20 kg) while they are undergoing procedures as defined in this Directive, if this separation is essential for scientific purposes. The period for which a dog is so constrained shall not exceed 4 hours at a time.

A nursing bitch and litter shall have the same space allowance as a single bitch of equivalent weight. The whelping pen shall be designed so that the bitch can move to an additional compartment or raised area away from the puppies.

Weight (kg)	Minimum enclosure size (m^2)	Minimum floor area for one or two animals (m^2)	For each additional animal add a minimum of (m^2)	Minimum height (m)	Date referred to in Article 33(2)
up to 20	4	4	2	2	1 January 2017
over 20	8	8	4	2	

Table 4.2.

Dogs — post-weaned stock

Weight of dog (kg)	Minimum enclosure size (m^2)	Minimum floor area/animal (m^2)	Minimum height (m)	Date referred to in Article 33(2)
up to 5	4	0,5	2	1 January 2017
over 5 to 10	4	1,0	2	
over 10 to 15	4	1,5	2	
over 15 to 20	4	2	2	
over 20	8	4	2	

5. Ferrets

Table 5.

Ferrets

	Minimum enclosure size (cm^2)	Minimum floor area per animal (cm^2)	Minimum height (cm)	Date referred to in Article 33(2)
Animals up to 600 g	4 500	1 500	50	1 January 2017
Animals over 600 g	4 500	3 000	50	
Adult males	6 000	6 000	50	
Jill and litter	5 400	5 400	50	

6. Non-human primates

Young non-human primates shall not be separated from their mothers until they are, depending on the species, 6 to 12 months old.

The environment shall enable non-human primates to carry out a complex daily programme of activity. The enclosure shall allow non-human primates to adopt as wide a behavioural repertoire as possible, provide it with a sense of security, and a suitably complex environment to allow the animal to run, walk, climb and jump.

Table 6.1.

Marmosets and tamarins

	Minimum floor area of enclosures for 1 (*) or 2 animals plus offspring up to 5 months old (m^2)	Minimum volume per additional animal over 5 months (m^3)	Minimum enclosure height (m) (**)	Date referred to in Article 33(2)
Marmosets	0,5	0,2	1,5	1 January 2017
Tamarins	1,5	0,2	1,5	

(*) Animals shall be kept singly only in exceptional circumstances.
(**) The top of the enclosure shall be at least 1,8 m from the floor.

For marmosets and tamarins, separation from the mother shall not take place before 8 months of age.

Table 6.2.

Squirrel monkeys

Minimum floor area for 1 (*) or 2 animals (m^2)	Minimum volume per additional animal over 6 months of age (m^3)	Minimum enclosure height (m)	Date referred to in Article 33(2)
2,0	0,5	1,8	1 January 2017

(*) Animals shall be kept singly only in exceptional circumstances.

For squirrel monkeys, separation from the mother shall not take place before 6 months of age.

Table 6.3.

Macaques and vervets (*)

	Minimum enclosure size (m^2)	Minimum enclosure volume (m^3)	Minimum volume per animal (m^3)	Minimum enclosure height (m)	Date referred to in Article 33(2)
Animals less than 3 yrs of age (**)	2,0	3,6	1,0	1,8	1 January 2017
Animals from 3 yrs of age (***)	2,0	3,6	1,8	1,8	
Animals held for breeding purposes (****)			3,5	2,0	

(*) Animals shall be kept singly only in exceptional circumstances.
(**) An enclosure of minimum dimensions may hold up to three animals.
(***) An enclosure of minimum dimensions may hold up to two animals.
(****) In breeding colonies no additional space/volume allowance is required for young animals up to 2 years of age housed with their mother.

For macaques and vervets, separation from the mother shall not take place before 8 months of age.

Table 6.4.

Baboons (*)

	Minimum enclosure size (m^2)	Minimum enclosure volume (m^3)	Minimum volume per animal (m^3)	Minimum enclosure height (m)	Date referred to in Article 33(2)
Animals less than 4 yrs of age (**)	4,0	7,2	3,0	1,8	1 January 2017
Animals from 4 yrs of age (**)	7,0	12,6	6,0	1,8	
Animals held for breeding purposes (***)			12,0	2,0	

(*) Animals shall be kept singly only in exceptional circumstances.
(**) An enclosure of minimum dimensions may hold up to 2 animals.
(***) In breeding colonies no additional space/volume allowance is required for young animals up to 2 years of age housed with their mothers.

For baboons, separation from the mother shall not take place before 8 months of age.

7. Farm animals

During agricultural research, when the aim of the project requires that the animals are kept under similar conditions to those under which commercial farm animals are kept, the keeping of the animals shall comply at least with the standards laid down in Directives 98/58/EC, 91/629/EEC ([1]) and 91/630/EEC ([2]).

([1]) Council Directive 91/629/EEC of 19 November 1991 laying down minimum standards for the protection of calves (OJ L 340, 11.12.1991, p. 28).
([2]) Council Directive 91/630/EEC of 19 November 1991 laying down minimum standards for the protection of pigs (OJ L 340, 11.12.1991, p. 33).

Table 7.1.

Cattle

Body weight (kg)	Minimum enclosure size (m^2)	Minimum floor area/animal (m^2/animal)	Trough space for ad-libitum feeding of polled cattle (m/animal)	Trough space for restricted feeding of polled cattle (m/animal)	Date referred to in Article 33(2)
up to 100	2,50	2,30	0,10	0,30	1 January 2017
over 100 to 200	4,25	3,40	0,15	0,50	
over 200 to 400	6,00	4,80	0,18	0,60	
over 400 to 600	9,00	7,50	0,21	0,70	
over 600 to 800	11,00	8,75	0,24	0,80	
over 800	16,00	10,00	0,30	1,00	

Table 7.2.

Sheep and goats

Body weight (kg)	Minimum enclosure size (m^2)	Minimum floor area/animal (m^2/animal)	Minimum partition height (m)	Trough space for ad-libitum feeding (m/animal)	Trough space for restricted feeding (m/animal)	Date referred to in Article 33(2)
less than 20	1,0	0,7	1,0	0,10	0,25	1 January 2017
over 20 to 35	1,5	1,0	1,2	0,10	0,30	
over 35 to 60	2,0	1,5	1,2	0,12	0,40	
over 60	3,0	1,8	1,5	0,12	0,50	

Table 7.3.

Pigs and minipigs

Live weight (kg)	Minimum enclosure size (*) (m^2)	Minimum floor area per animal (m^2/animal)	Minimum lying space per animal (in, thermoneutral conditions) (m^2/animal)	Date referred to in Article 33(2)
Up to 5	2,0	0,20	0,10	1 January 2017
over 5 to 10	2,0	0,25	0,11	
over 10 to 20	2,0	0,35	0,18	
over 20 to 30	2,0	0,50	0,24	
over 30 to 50	2,0	0,70	0,33	
over 50 to 70	3,0	0,80	0,41	
over 70 to 100	3,0	1,00	0,53	

Live weight (kg)	Minimum enclosure size (*) (m^2)	Minimum floor area per animal (m^2/animal)	Minimum lying space per animal (in, thermoneutral conditions) (m^2/animal)	Date referred to in Article 33(2)
over 100 to 150	4,0	1,35	0,70	
over 150	5,0	2,50	0,95	
Adult (conventional) boars	7,5		1,30	

(*) Pigs may be confined in smaller enclosures for short periods of time, for example by partitioning the main enclosure using dividers, when justified on veterinary or experimental grounds, for example where individual food consumption is required.

Table 7.4.

Equines

The shortest side shall be a minimum of 1,5 times the wither height of the animal. The height of indoor enclosures shall allow animals to rear to their full height.

Wither height (m)	Minimum floor area/animal (m^2/animal)			Minimum enclosure height (m)	Date referred to in Article 33(2)
	For each animal held singly or in groups of up to 3 animals	For each animal held in groups of 4 or more animals	Foaling box/mare with foal		
1,00 to 1,40	9,0	6,0	16	3,00	1 January 2017
over 1,40 to 1,60	12,0	9,0	20	3,00	
over 1,60	16,0	$(2 \times WH)^2$ (*)	20	3,00	

(*) To ensure adequate space is provided, space allowances for each individual animal shall be based on height to withers (WH).

8. Birds

During agricultural research, when the aim of the project requires that the animals are kept under similar conditions to those under which commercial farm animals are kept, the keeping of the animals shall comply at least with the standards laid down in Directives 98/58/EC, 1999/74/EC ([1]) and 2007/43/EC ([2]).

Table 8.1.

Domestic fowl

Where these minimum enclosure sizes cannot be provided for scientific reasons, the duration of the confinement shall be justified by the experimenter in consultation with veterinary staff. In such circumstances, birds can be housed in smaller enclosures containing appropriate enrichment and with a minimum floor area of 0,75 m^2.

Body mass (g)	Minimum enclosure size (m^2)	Minimum area per bird (m^2)	Minimum height (cm)	Minimum length of feed trough per bird (cm)	Date referred to in Article 33(2)
Up to 200	1,00	0,025	30	3	1 January 2017
over 200 to 300	1,00	0,03	30	3	
over 300 to 600	1,00	0,05	40	7	

([1]) Council Directive 1999/74/EC of 19 July 1999 laying down minimum standards for the protection of laying hens (OJ L 203, 3.8.1999, p. 53).
([2]) Council Directive 2007/43/EC of 28 June 2007 laying down minimum rules for the protection of chickens kept for meat production (OJ L 182, 12.7.2007, p. 19).

Body mass (g)	Minimum enclosure size (m^2)	Minimum area per bird (m^2)	Minimum height (cm)	Minimum length of feed trough per bird (cm)	Date referred to in Article 33(2)
over 600 to 1 200	2,00	0,09	50	15	
over 1 200 to 1 800	2,00	0,11	75	15	
over 1 800 to 2 400	2,00	0,13	75	15	
over 2 400	2,00	0,21	75	15	

Table 8.2.

Domestic turkeys

All enclosure sides shall be at least 1,5 m long. Where these minimum enclosures sizes cannot be provided for scientific reasons, the duration of the confinement shall be justified by the experimenter in consultation with veterinary staff. In such circumstances, birds can be housed in smaller enclosures containing appropriate enrichment and with a minimum floor area of 0,75 m^2 and a minimum height of 50 cm for birds below 0,6 kg, 75 cm for birds below 4 kg, and 100 cm for birds over 4 kg. These can be used to house small groups of birds in accordance with the space allowances given in table 8.2.

Body mass (kg)	Minimum enclosure size (m^2)	Minimum area per bird (m^2)	Minimum height (cm)	Minimum length of feed trough per bird (cm)	Date referred to in Article 33(2)
Up to 0,3	2,00	0,13	50	3	1 January 2017
over 0,3 to 0,6	2,00	0,17	50	7	
over 0,6 to 1	2,00	0,30	100	15	
over 1 to 4	2,00	0,35	100	15	
over 4 to 8	2,00	0,40	100	15	
over 8 to 12	2,00	0,50	150	20	
over 12 to 16	2,00	0,55	150	20	
over 16 to 20	2,00	0,60	150	20	
over 20	3,00	1,00	150	20	

Table 8.3.

Quails

Body mass (g)	Minimum enclosure size (m^2)	Area per bird pair-housed (m^2)	Area per additional bird group-housed (m^2)	Minimum height (cm)	Minimum length of trough per bird (cm)	Date referred to in Article 33(2)
Up to 150	1,00	0,5	0,10	20	4	1 January 2017
Over 150	1,00	0,6	0,15	30	4	

Table 8.4.

Ducks and geese

Where these minimum enclosures sizes cannot be provided for scientific reasons, the duration of the confinement shall be justified by the experimenter in consultation with veterinary staff. In such circumstances, birds can be housed in smaller enclosures containing appropriate enrichment and with a minimum floor area of 0,75 m^2. These can be used to house small groups of birds in accordance with the space allowances given in table 8.4.

Body mass (g)	Minimum enclosure size (m^2)	Area per bird (m^2) (*)	Minimum height (cm)	Minimum length of feed trough per bird (cm)	Date referred to in Article 33(2)
Ducks					1 January 2017
Up to 300	2,00	0,10	50	10	
Over 300 to 1 200 (**)	2,00	0,20	200	10	
Over 1 200 to 3 500	2,00	0,25	200	15	
Over 3 500	2,00	0,50	200	15	
Geese					
Up to 500	2,00	0,20	200	10	
Over 500 to 2 000	2,00	0,33	200	15	
Over 2 000	2,00	0,50	200	15	

(*) This shall include a pond of minimum area 0,5 m^2 per 2 m^2 enclosure with a minimum depth of 30 cm. The pond may contribute up to 50 % of the minimum enclosure size.
(**) Pre-fledged birds may be held in enclosures with a minimum height of 75 cm.

Table 8.5.

Ducks and geese: Minimum pond sizes (*)

	Area (m^2)	Depth (cm)
Ducks	0,5	30
Geese	0,5	from 10 to 30

(*) Pond sizes are per 2 m^2 enclosure. The pond may contribute up to 50 % of the minimum enclosure size.

Table 8.6.

Pigeons

Enclosures shall be long and narrow (for example 2 m by 1 m) rather than square to allow birds to perform short flights.

Group size	Minimum enclosure size (m^2)	Minimum height (cm)	Minimum length of food trough per bird (cm)	Minimum length of perch per bird (cm)	Date referred to in Article 33(2)
Up to 6	2	200	5	30	1 January 2017

Group size	Minimum enclosure size (m^2)	Minimum height (cm)	Minimum length of food trough per bird (cm)	Minimum length of perch per bird (cm)	Date referred to in Article 33(2)
from 7 to 12	3	200	5	30	
for each additional bird above 12	0,15		5	30	

Table 8.7.

Zebra finches

Enclosures shall be long and narrow (for example 2 m by 1 m) to enable birds to perform short flights. For breeding studies, pairs may be housed in smaller enclosures containing appropriate enrichment with a minimum floor area of 0,5 m^2 and a minimum height of 40 cm. The duration of the confinement shall be justified by the experimenter in consultation with veterinary staff.

Group size	Minimum enclosure size (m^2)	Minimum height (cm)	Minimum number of feeders	Date referred to in Article 33(2)
Up to 6	1,0	100	2	1 January 2017
7 to 12	1,5	200	2	
13 to 20	2,0	200	3	
for each additional bird above 20	0,05		1 per 6 birds	

9. Amphibians

Table 9.1.

Aquatic urodeles

Body length (*) (cm)	Minimum water surface area (cm^2)	Minimum water surface area for each additional animal in group-holding (cm^2)	Minimum water depth (cm)	Date referred to in Article 33(2)
Up to 10	262,5	50	13	1 January 2017
over 10 to 15	525	110	13	
over 15 to 20	875	200	15	
over 20 to 30	1 837,5	440	15	
Over 30	3 150	800	20	

(*) Measured from snout to vent.

Table 9.2.

Aquatic anurans (*)

Body length (**) (cm)	Minimum water surface area (cm^2)	Minimum water surface area for each additional animal in group-holding (cm^2)	Minimum water depth (cm)	Date referred to in Article 33(2)
Less than 6	160	40	6	1 January 2017
from 6 to 9	300	75	8	

Body length (**) (cm)	Minimum water surface area (cm^2)	Minimum water surface area for each additional animal in group-holding (cm^2)	Minimum water depth (cm)	Date referred to in Article 33(2)
over 9 to 12	600	150	10	
over 12	920	230	12,5	

(*) These conditions apply to holding (i.e. husbandry) tanks but not to those tanks used for natural mating and super-ovulation for reasons of efficiency, as the latter procedures require smaller individual tanks. Space requirements determined for adults in the indicated size categories; juveniles and tadpoles shall either be excluded, or dimensions altered according to the scaling principle.

(**) Measured from snout to vent.

Table 9.3.

Semi-aquatic anurans

Body length (*) (cm)	Minimum enclosure size (**) (cm^2)	Minimum area for each additional animal in group holding (cm^2)	Minimum enclosure height (***) (cm)	Minimum water depth (cm)	Date referred to in Article 33(2)
up to 5,0	1 500	200	20	10	1 January 2017
over 5,0 to 7,5	3 500	500	30	10	
Over 7,5	4 000	700	30	15	

(*) Measured from snout to vent.

(**) One-third land division, two-thirds water division sufficient for animals to submerge.

(***) Measured from the surface of the land division up to the inner part of the top of the terrarium; furthermore, the height of the enclosures shall be adapted to the interior design.

Table 9.4.

Semi-terrestrial anurans

Body length (*) (cm)	Minimum enclosure size (**) (cm^2)	Minimum area for each additional animal in group-holding (cm^2)	Minimum enclosure height (***) (cm)	Minimum water depth (cm)	Date referred to in Article 33(2)
Up to 5,0	1 500	200	20	10	1 January 2017
over 5,0 to 7,5	3 500	500	30	10	
over 7,5	4 000	700	30	15	

(*) Measured from snout to vent.

(**) Two-thirds land division, one-third water division sufficient for animals to submerge.

(***) Measured from the surface of the land division up to the inner part of the top of the terrarium; furthermore, the height of the enclosures shall be adapted to the interior design.

Table 9.5.

Arboreal anurans

Body length (*) (cm)	Minimum enclosure size (**) (cm^2)	Minimum area for each additional animal in group-holding (cm^2)	Minimum enclosure height (***) (cm)	Date referred to in Article 33(2)
up to 3,0	900	100	30	1 January 2017
Over 3,0	1 500	200	30	

(*) Measured from snout to vent.
(**) Two-thirds land division, one-third pool division sufficient for animals to submerge.
(***) Measured from the surface of the land division up to the inner part of the top of the terrarium; furthermore, the height of the enclosures shall be adapted to the interior design.

10. Reptiles

Table 10.1.

Aquatic chelonians

Body length (*) (cm)	Minimum water surface area (cm^2)	Minimum water surface area for each additional animal in group holding (cm^2)	Minimum water depth (cm)	Date referred to in Article 33(2)
up to 5	600	100	10	1 January 2017
Over 5 to 10	1 600	300	15	
Over 10 to 15	3 500	600	20	
Over 15 to 20	6 000	1 200	30	
Over 20 to 30	10 000	2 000	35	
Over 30	20 000	5 000	40	

(*) Measured in a straight line from the front edge to the back edge of the shell.

Table 10.2.

Terrestrial snakes

Body length (*) (cm)	Minimum floor area (cm^2)	Minimum area for each additional animal in group-holding (cm^2)	Minimum enclosure height (**) (cm)	Date referred to in Article 33(2)
up to 30	300	150	10	1 January 2017
Over 30 to 40	400	200	12	
Over 40 to 50	600	300	15	
Over 50 to 75	1 200	600	20	
Over 75	2 500	1 200	28	

(*) Measured from snout to tail.
(**) Measured from the surface of the land division up to the inner part of the top of the terrarium; furthermore, the height of the enclosure shall be adapted to the interior design.

11. Fish

11.1. Water supply and quality

Adequate water supply of suitable quality shall be provided at all times. Water flow in re-circulatory systems or filtration within tanks shall be sufficient to ensure that water quality parameters are maintained within acceptable levels. Water supply shall be filtered or treated to remove substances harmful to fish, where necessary. Water-quality parameters shall at all times be within the acceptable range that sustains normal activity and physiology for a given species and stage of development. The water flow shall be appropriate to enable fish to swim correctly and to maintain normal behaviour. Fish shall be given an appropriate time for acclimatisation and adaptation to changes in water-quality conditions.

11.2. Oxygen, nitrogen compounds, pH, and salinity

Oxygen concentration shall be appropriate to the species and to the context in which the fish are held. Where necessary, supplementary aeration of tank water shall be provided. The concentrations of nitrogen compounds shall be kept low.

The pH level shall be adapted to the species and kept as stable as possible. The salinity shall be adapted to the requirements of the fish species and to the life stage of the fish. Changes in salinity shall take place gradually.

11.3. Temperature, lighting, noise

Temperature shall be maintained within the optimal range for the fish species concerned and kept as stable as possible. Changes in temperature shall take place gradually. Fish shall be maintained on an appropriate photo-period. Noise levels shall be kept to a minimum and, where possible, equipment causing noise or vibration, such as power generators or filtration systems, shall be separate from the fish-holding tanks.

11.4. Stocking density and environmental complexity

The stocking density of fish shall be based on the total needs of the fish in respect of environmental conditions, health and welfare. Fish shall have sufficient water volume for normal swimming, taking account of their size, age, health and feeding method. Fish shall be provided with an appropriate environmental enrichment, such as hiding places or bottom substrate, unless behavioural traits suggest none is required.

11.5. Feeding and handling

Fish shall be fed a diet suitable for the fish at an appropriate feeding rate and frequency. Particular attention shall be given to feeding of larval fish during any transition from live to artificial diets. Handling of fish shall be kept to a minimum.

ANNEX IV

METHODS OF KILLING ANIMALS

1. In the process of killing animals, methods listed in the table below shall be used.

   Methods other than those listed in the table may be used:

   (a) on unconscious animals, providing the animal does not regain consciousness before death;

   (b) on animals used in agricultural research, when the aim of the project requires that the animals are kept under similar conditions to those under which commercial farm animals are kept; these animals may be killed in accordance with the requirements laid down in Annex I to Council Regulation (EC) No 1099/2009 of 24 September 2009 on the protection of animals at the time of killing [1].

2. The killing of animals shall be completed by one of the following methods:

   (a) confirmation of permanent cessation of the circulation;

   (b) destruction of the brain;

   (c) dislocation of the neck;

   (d) exsanguination; or

   (e) confirmation of the onset of *rigor mortis*.

3. Table

Animals-remarks/methods	Fish	Amphibians	Reptiles	Birds	Rodents	Rabbits	Dogs, cats, ferrets and foxes	Large mammals	Non-human primates
Anaesthetic overdose	(1)	(1)	(1)	(1)	(1)	(1)	(1)	(1)	(1)
Captive bolt	X	X	(2)	X	X		X		X
Carbon dioxide	X	X	X		(3)	X	X	X	X
Cervical dislocation	X	X	X	(4)	(5)	(6)	X	X	X
Concussion/percussive blow to the head				(7)	(8)	(9)	(10)	X	X
Decapitation	X	X	X	(11)	(12)	X	X	X	X
Electrical stunning	(13)	(13)	X	(13)	X	(13)	(13)	(13)	X
Inert gases (Ar, N_2)	X	X	X			X	X	(14)	X
Shooting with a free bullet with appropriate rifles, guns and ammunition	X	X	(15)	X	X	X	(16)	(15)	X

[1] OJ L 303, 18.11.2009, p. 1.

Requirements

1. Shall, where appropriate, be used with prior sedation.
2. Only to be used on large reptiles.
3. Only to be used in gradual fill. Not to be used for foetal and neonate rodents.
4. Only to be used for birds under 1 kg. Birds over 250 g shall be sedated.
5. Only to be used for rodents under 1 kg. Rodents over 150 g shall be sedated.
6. Only to be used for rabbits under 1 kg. Rabbits over 150 g shall be sedated.
7. Only to be used for birds under 5 kg.
8. Only to be used for rodents under 1 kg.
9. Only to be used for rabbits under 5 kg.
10. Only to be used on neonates.
11. Only to be used for birds under 250 g.
12. Only to be used if other methods are not possible.
13. Specialised equipment required.
14. Only to be used on pigs.
15. Only to be used in field conditions by experienced marksmen.
16. Only to be used in field conditions by experienced marksmen when other methods are not possible.

ANNEX V

LIST OF ELEMENTS REFERRED TO IN ARTICLE 23(3)

1. National legislation in force relevant to the acquisition, husbandry, care and use of animals for scientific purposes.
2. Ethics in relation to human-animal relationship, intrinsic value of life and arguments for and against the use of animals for scientific purposes.
3. Basic and appropriate species-specific biology in relation to anatomy, physiological features, breeding, genetics and genetic alteration.
4. Animal behaviour, husbandry and enrichment.
5. Species-specific methods of handling and procedures, where appropriate.
6. Animal health management and hygiene.
7. Recognition of species-specific distress, pain and suffering of most common laboratory species.
8. Anaesthesia, pain relieving methods and killing.
9. Use of humane end-points.
10. Requirement of replacement, reduction and refinement.
11. Design of procedures and projects, where appropriate.

ANNEX VI

LIST OF ELEMENTS REFERRED TO IN ARTICLE 37(1)(c)

1. Relevance and justification of the following:

   (a) use of animals including their origin, estimated numbers, species and life stages;

   (b) procedures.

2. Application of methods to replace, reduce and refine the use of animals in procedures.

3. The planned use of anaesthesia, analgesia and other pain relieving methods.

4. Reduction, avoidance and alleviation of any form of animal suffering, from birth to death where appropriate.

5. Use of humane end-points.

6. Experimental or observational strategy and statistical design to minimise animal numbers, pain, suffering, distress and environmental impact where appropriate.

7. Reuse of animals and the accumulative effect thereof on the animals.

8. The proposed severity classification of procedures.

9. Avoidance of unjustified duplication of procedures where appropriate.

10. Housing, husbandry and care conditions for the animals.

11. Methods of killing.

12. Competence of persons involved in the project.

ANNEX VII

DUTIES AND TASKS OF THE UNION REFERENCE LABORATORY

1. The Union Reference Laboratory referred to in Article 48 is the Commission's Joint Research Centre.

2. The Union Reference Laboratory shall be responsible, in particular, for:

   (a) coordinating and promoting the development and use of alternatives to procedures including in the areas of basic and applied research and regulatory testing;

   (b) coordinating the validation of alternative approaches at Union level;

   (c) acting as a focal point for the exchange of information on the development of alternative approaches;

   (d) setting up, maintaining and managing public databases and information systems on alternative approaches and their state of development;

   (e) promoting dialogue between legislators, regulators, and all relevant stakeholders, in particular, industry, biomedical scientists, consumer organisations and animal-welfare groups, with a view to the development, validation, regulatory acceptance, international recognition, and application of alternative approaches.

3. The Union Reference Laboratory shall participate in the validation of alternative approaches.

ANNEX VIII

SEVERITY CLASSIFICATION OF PROCEDURES

The severity of a procedure shall be determined by the degree of pain, suffering, distress or lasting harm expected to be experienced by an individual animal during the course of the procedure.

Section I: Severity categories

Non-recovery:

Procedures which are performed entirely under general anaesthesia from which the animal shall not recover consciousness shall be classified as 'non-recovery'.

Mild:

Procedures on animals as a result of which the animals are likely to experience short-term mild pain, suffering or distress, as well as procedures with no significant impairment of the well-being or general condition of the animals shall be classified as 'mild'.

Moderate:

Procedures on animals as a result of which the animals are likely to experience short-term moderate pain, suffering or distress, or long-lasting mild pain, suffering or distress as well as procedures that are likely to cause moderate impairment of the well-being or general condition of the animals shall be classified as 'moderate'.

Severe:

Procedures on animals as a result of which the animals are likely to experience severe pain, suffering or distress, or long-lasting moderate pain, suffering or distress as well as procedures, that are likely to cause severe impairment of the well-being or general condition of the animals shall be classified as 'severe'.

Section II: Assignment criteria

The assignment of the severity category shall take into account any intervention or manipulation of an animal within a defined procedure. It shall be based on the most severe effects likely to be experienced by an individual animal after applying all appropriate refinement techniques.

When assigning a procedure to a particular category, the type of procedure and a number of other factors shall be taken into account. All these factors shall be considered on a case-by-case basis.

The factors related to the procedure shall include:

— type of manipulation, handling,

— nature of pain, suffering, distress or lasting harm caused by (all elements of) the procedure, and its intensity, the duration, frequency and multiplicity of techniques employed,

— cumulative suffering within a procedure,

— prevention from expressing natural behaviour including restrictions on the housing, husbandry and care standards.

Examples are given in Section III of procedures assigned to each of the severity categories on the basis of factors related to the type of the procedure alone. They shall provide the first indication as to what classification would be the most appropriate for a certain type of procedure.

However, for the purposes of the final severity classification of the procedure, the following additional factors, assessed on a case-by-case basis, shall also be taken into account:

— type of species and genotype,

— maturity, age and gender of the animal,

— training experience of the animal with respect to the procedure,

— if the animal is to be reused, the actual severity of the previous procedures,

— the methods used to reduce or eliminate pain, suffering and distress, including refinement of housing, husbandry and care conditions,

— humane end-points.

Section III:

Examples of different types of procedure assigned to each of the severity categories on the basis of factors related to the type of the procedure

1. Mild:

 (a) administration of anaesthesia except for the sole purpose of killing;

 (b) pharmacokinetic study where a single dose is administered and a limited number of blood samples are taken (totalling < 10 % of circulating volume) and the substance is not expected to cause any detectable adverse effect;

 (c) non-invasive imaging of animals (e.g. MRI) with appropriate sedation or anaesthesia;

 (d) superficial procedures, e.g. ear and tail biopsies, non-surgical subcutaneous implantation of mini-pumps and transponders;

 (e) application of external telemetry devices that cause only minor impairment to the animals or minor interference with normal activity and behaviour;

 (f) administration of substances by subcutaneous, intramuscular, intraperitoneal routes, gavage and intravenously via superficial blood vessels, where the substance has no more than mild impact on the animal, and the volumes are within appropriate limits for the size and species of the animal;

 (g) induction of tumours, or spontaneous tumours, that cause no detectable clinical adverse effects (e.g. small, subcutaneous, non-invasive nodules);

 (h) breeding of genetically altered animals, which is expected to result in a phenotype with mild effects;

 (i) feeding of modified diets, that do not meet all of the animals' nutritional needs and are expected to cause mild clinical abnormality within the time-scale of the study;

 (j) short-term (< 24h) restraint in metabolic cages;

 (k) studies involving short-term deprivation of social partners, short-term solitary caging of adult rats or mice of sociable strains;

(l) models which expose animals to noxious stimuli which are briefly associated with mild pain, suffering or distress, and which the animals can successfully avoid;

(m) a combination or accumulation of the following examples may result in classification as 'mild':

(i) assessing body composition by non-invasive measures and with minimal restraint;

(ii) monitoring ECG with non-invasive techniques with minimal or no restraint of habituated animals;

(iii) application of external telemetry devices that are expected to cause no impairment to socially adapted animals and do not interfere with normal activity and behaviour;

(iv) breeding genetically altered animals which are expected to have no clinically detectable adverse phenotype;

(v) adding inert markers in the diet to follow passage of digesta;

(vi) withdrawal of food for < 24h in adult rats;

(vii) open field testing.

2. Moderate:

(a) frequent application of test substances which produce moderate clinical effects, and withdrawal of blood samples (> 10 % of circulating volume) in a conscious animal within a few days without volume replacement;

(b) acute dose-range finding studies, chronic toxicity/carcinogenicity tests, with non-lethal end-points;

(c) surgery under general anaesthesia and appropriate analgesia, associated with post surgical pain, suffering or impairment of general condition. Examples include: thoracotomy, craniotomy, laparotomy, orchidectomy, lymphadenectomy, thyroidectomy, orthopaedic surgery with effective stabilisation and wound management, organ transplantation with effective management of rejection, surgical implantation of catheters, or biomedical devices (e.g. telemetry transmitters, minipumps etc.);

(d) models of induction of tumours, or spontaneous tumours, that are expected to cause moderate pain or distress or moderate interference with normal behaviour;

(e) irradiation or chemotherapy with a sublethal dose, or with an otherwise lethal dose but with reconstitution of the immune system. Adverse effects would be expected to be mild or moderate and would be short-lived (< 5 days);

(f) breeding of genetically altered animals which are expected to result in a phenotype with moderate effects;

(g) creation of genetically altered animals through surgical procedures;

(h) use of metabolic cages involving moderate restriction of movement over a prolonged period (up to 5 days);

(i) studies with modified diets that do not meet all of the animals' nutritional needs and are expected to cause moderate clinical abnormality within the time-scale of the study;

(j) withdrawal of food for 48 hours in adult rats;

(k) evoking escape and avoidance reactions where the animal is unable to escape or avoid the stimulus, and are expected to result in moderate distress.

3. Severe:

(a) toxicity testing where death is the end-point, or fatalities are to be expected and severe pathophysiological states are induced. For example, single dose acute toxicity testing (see OECD testing guidelines);

(b) testing of device where failure may cause severe pain, distress or death of the animal (e.g. cardiac assist devices);

(c) vaccine potency testing characterised by persistent impairment of the animal's condition, progressive disease leading to death, associated with long-lasting moderate pain, distress or suffering;

(d) irradiation or chemotherapy with a lethal dose without reconstitution of the immune system, or reconstitution with production of graft versus host disease;

(e) models with induction of tumours, or with spontaneous tumours, that are expected to cause progressive lethal disease associated with long-lasting moderate pain, distress or suffering. For example tumours causing cachexia, invasive bone tumours, tumours resulting in metastatic spread, and tumours that are allowed to ulcerate;

(f) surgical and other interventions in animals under general anaesthesia which are expected to result in severe or persistent moderate postoperative pain, suffering or distress or severe and persistent impairment of the general condition of the animals. Production of unstable fractures, thoracotomy without adequate analgesia, or trauma to produce multiple organ failure;

(g) organ transplantation where organ rejection is likely to lead to severe distress or impairment of the general condition of the animals (e.g. xenotransplantation);

(h) breeding animals with genetic disorders that are expected to experience severe and persistent impairment of general condition, for example Huntington's disease, Muscular dystrophy, chronic relapsing neuritis models;

(i) use of metabolic cages involving severe restriction of movement over a prolonged period;

(j) inescapable electric shock (e.g. to produce learned helplessness);

(k) complete isolation for prolonged periods of social species e.g. dogs and non-human primates;

(l) immobilisation stress to induce gastric ulcers or cardiac failure in rats;

(m) forced swim or exercise tests with exhaustion as the end-point.
