# Supplementary material for: Compensatory Fatty Acid Metabolism and Hepatic Gene Expression in African Catfish (Clarias gariepinus) Fed Marine‐Ingredient‐Free Circular Diets Low in EPA and DHA
Source: Aquac Nutr. 2026 Jun 11;2026:4162985. doi: 10.1155/anu/4162985 (PMC13259715; doi:10.1155/anu/4162985)
Supplement: Supplementary file 3 — Supporting Information 3 Figure S1: Expression of the housekeeping gene ef1a (elongation factor 1α) over the course of the trial. Figure S2: Development of RAS water temperature over the course of the trial. Figure S3: Development of RAS water oxygen concentration over the course of the trial. Figure S4: Development of RAS water pH over the course of the trial. Figure S5: Development of RAS water electrical conductivity over the course of the trial. Figure S6: Cumulative dissolved nutrient release per kg of feed input over the course of the trial; corrected for the influence of daily water exchange and tap water nutrient introduction. (A) Total inorganic nitrogen (TIN), (B) Soluble reactive phosphorus (SRP), (C) Potassium (K), (D) Magnesium (Mg), (E) Calcium (Ca), and (F) Sulfur (S). Figure S7: Development of fillet dry matter content (% wet weight) over the course of the trial. Figure S8: Relative proportion of fatty acid (FA) groups and certain FAs as a percentage (%) of total measured FAs in the fillet of the fish. Table S1: Fillet fatty acid content for week 2, 4, and 6 of the trial (g/kg DM). Table S2: Liver fatty acid content for week 2, 4, and 6 of the trial (g/kg DM). [file ANU-2026-4162985-s003.docx]

Supplementary materials

Christopher Shaw ^a,*^, Klaus Knopf ^a,b^, Sven Wuertz ^a^, Koushik Roy ^c^, Radek Gebauer ^c^, Tobias Goldhammer ^a^, Viola Schöning ^a^, Wibke Kleiner ^a^, Marvin Sens ^a^, Christian Ulrichs ^d^ and Werner Kloas ^a,b,e^

^a^ Leibniz Institute of Freshwater Ecology and Inland Fisheries, Berlin, Germany

^b^ Albrecht Daniel Thaer Institute of Agricultural and Horticultural Sciences, Humboldt University Berlin, Berlin, Germany

^c^ University of South Bohemia in Ceske Budejovice, Faculty of Fisheries and Protection of Waters, South Bohemian Research Center of Aquaculture and Biodiversity of Hydrocenoses, Institute of Aquaculture and Protection of Waters, České Budějovice 370 05, Czech Republic

^d^ Faculty of Life Sciences, Division Urban Plant Ecophysiology, Humboldt-University of Berlin Lentzeallee 55/57, Berlin 14195, Germany

^e^ Institute of Biology, Humboldt University Berlin, Berlin, Germany

^*^ Correspondence: christopher.shaw@igb-berlin.de; Tel.: +49-160-599-8467

**Table of contents**

**Figure S1.** Expression of the housekeeping gene *ef1a* (elongation factor 1α) over the course of the trial.

**Figure S2.** Development of RAS water temperature over the course of the trial.

**Figure S3.** Development of RAS water oxygen concentration over the course of the trial.

**Figure S4.** Development of RAS water pH over the course of the trial.

**Figure S5.** Development of RAS water electrical conductivity over the course of the trial.

**Figure S6.** Cumulative dissolved nutrient release per kg of feed input over the course of the trial; corrected for the influence of daily water exchange and tap water nutrient introduction. (A) total inorganic nitrogen (TIN), (B) soluble reactive phosphorus (SRP), (C) potassium (K), (D) magnesium (Mg), (E) calcium (Ca) and (F) sulfur (S).

**Figure S7.** Development of filet dry matter content (% wet weight) over the course of the trial.

**Figure S8.** Relative proportion of fatty acid (FA) groups and certain FAs as a percentage (%) of total measured FAs in the filet of the fish.

**Table S1.** Filet fatty acid content for week 2, 4 and 6 of the trial (g/kg DM).

**Table S2.** Liver fatty acid content for week 2, 4 and 6 of the trial (g/kg DM).*
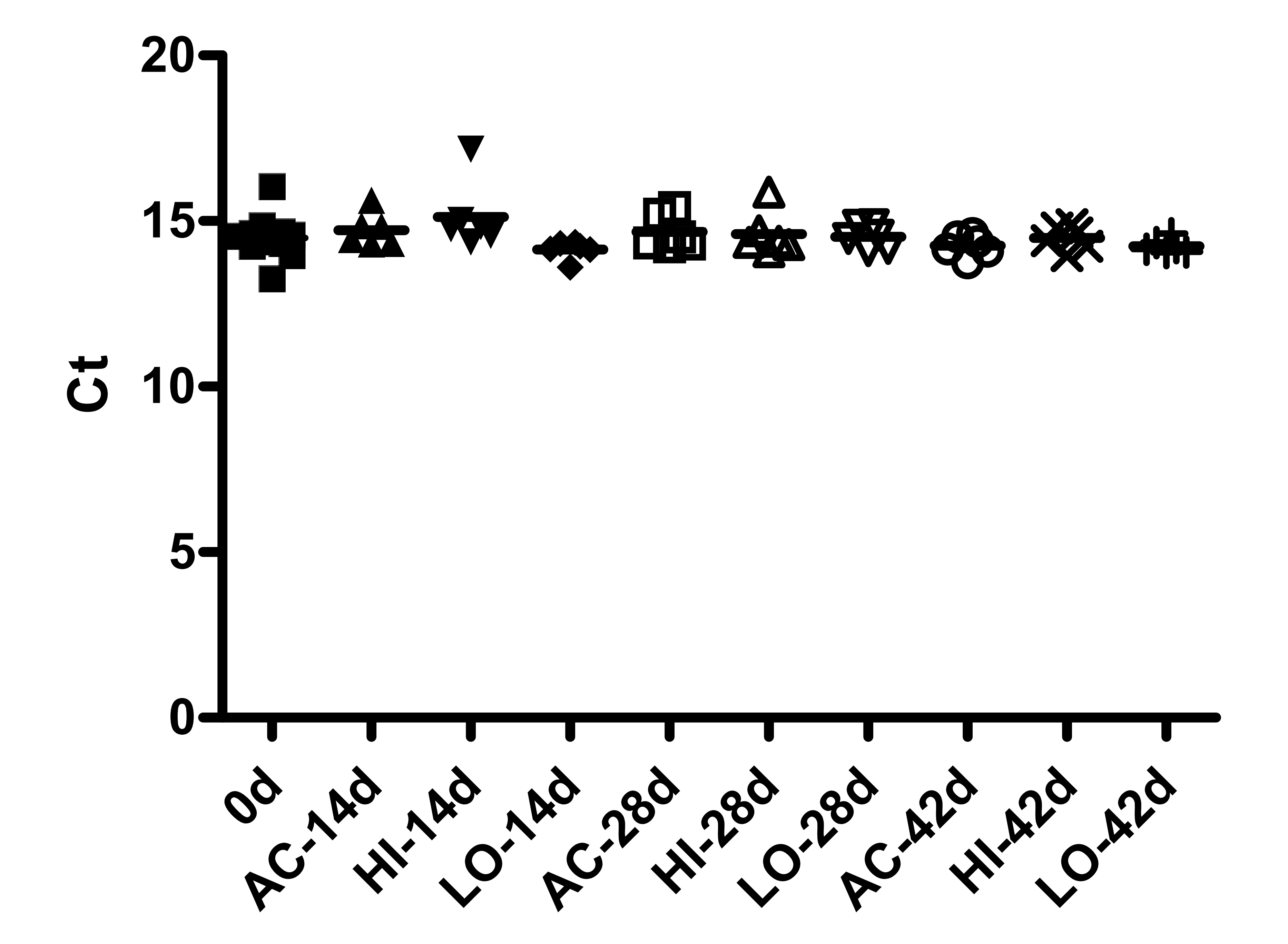
*

**Figure S1.** Expression of the housekeeping gene *ef1a* (elongation factor 1α) over the course of the trial. Tukey’s multiple comparison test showed no significant differences between any of the dietary treatments.

**Figure S2.** Development of RAS water temperature over the course of the trial. Error bars represent standard deviations; *n* = 3.

**Figure S3.** Development of RAS water oxygen concentration over the course of the trial. Error bars represent standard deviations; *n* = 3.

**Figure S4.** Development of RAS water pH over the course of the trial. Error bars represent standard deviations; *n* = 3.

**Figure S5.** Development of RAS water electrical conductivity over the course of the trial. Error bars represent standard deviations; *n* = 3.


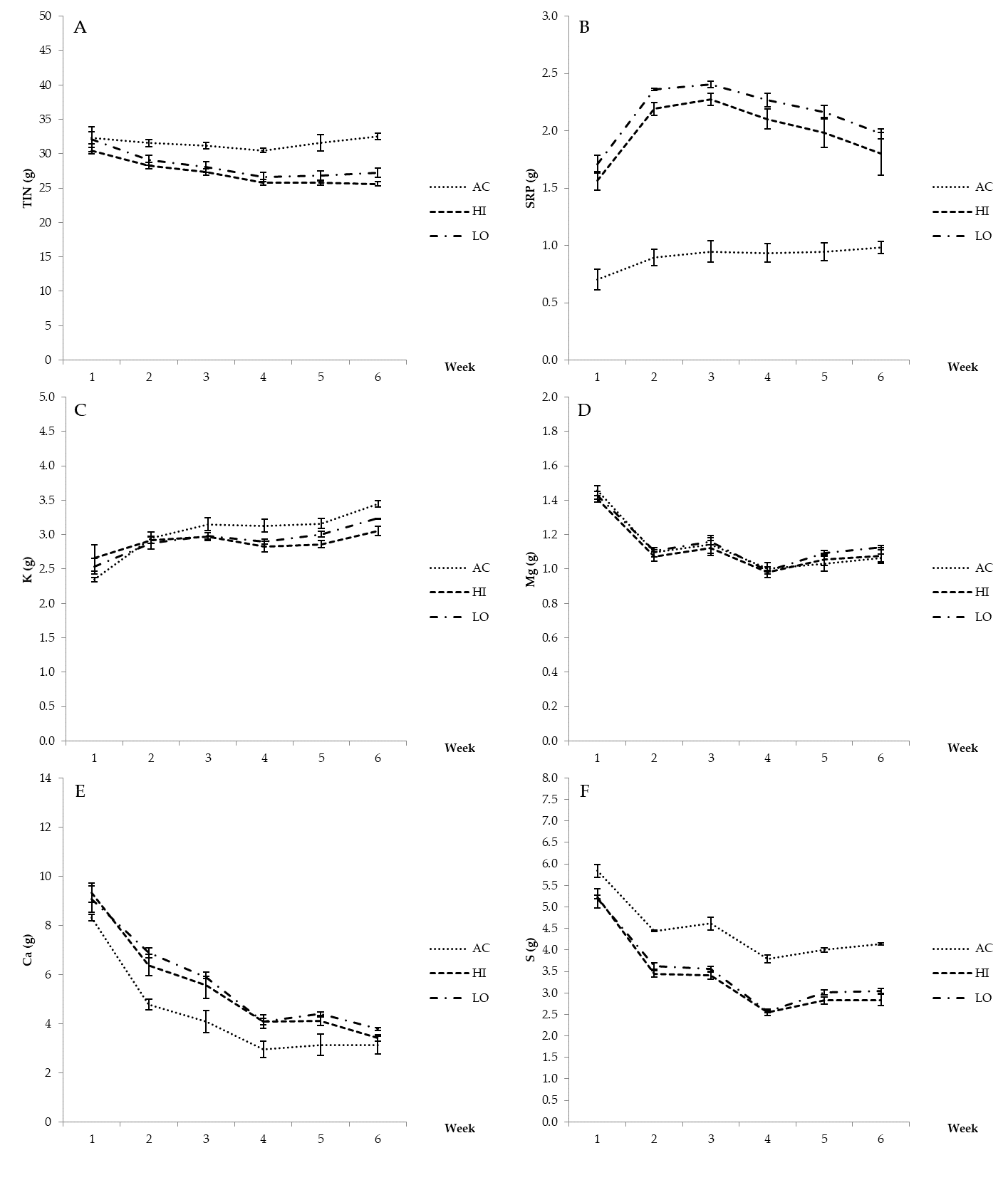
**Figure S6.** Cumulative dissolved nutrient release per kg of feed input over the course of the trial; corrected for the influence of daily water exchange and tap water nutrient introduction. (**A**) total inorganic nitrogen (TIN), (**B**) soluble reactive phosphorus (SRP), (**C**) potassium (K), (**D**) magnesium (Mg), (**E**) calcium (Ca) and (**F**) sulfur (S). Error bars represent standard deviations; *n* = 3. Note: The final data point (week 6) represents the dissolved nutrient release (g/kg of feed) over the entire trial and is given in Table 7 of the main text.

**Figure S7.** Development of filet dry matter content (% wet weight) over the course of the trial. Error bars represent standard deviations; *n* = 3. Note: Results, although very similar, do not exactly match the DM content in table 6 for the start and end of the trial, since the filet DM content presented in this figure was determined independently at the IGB.


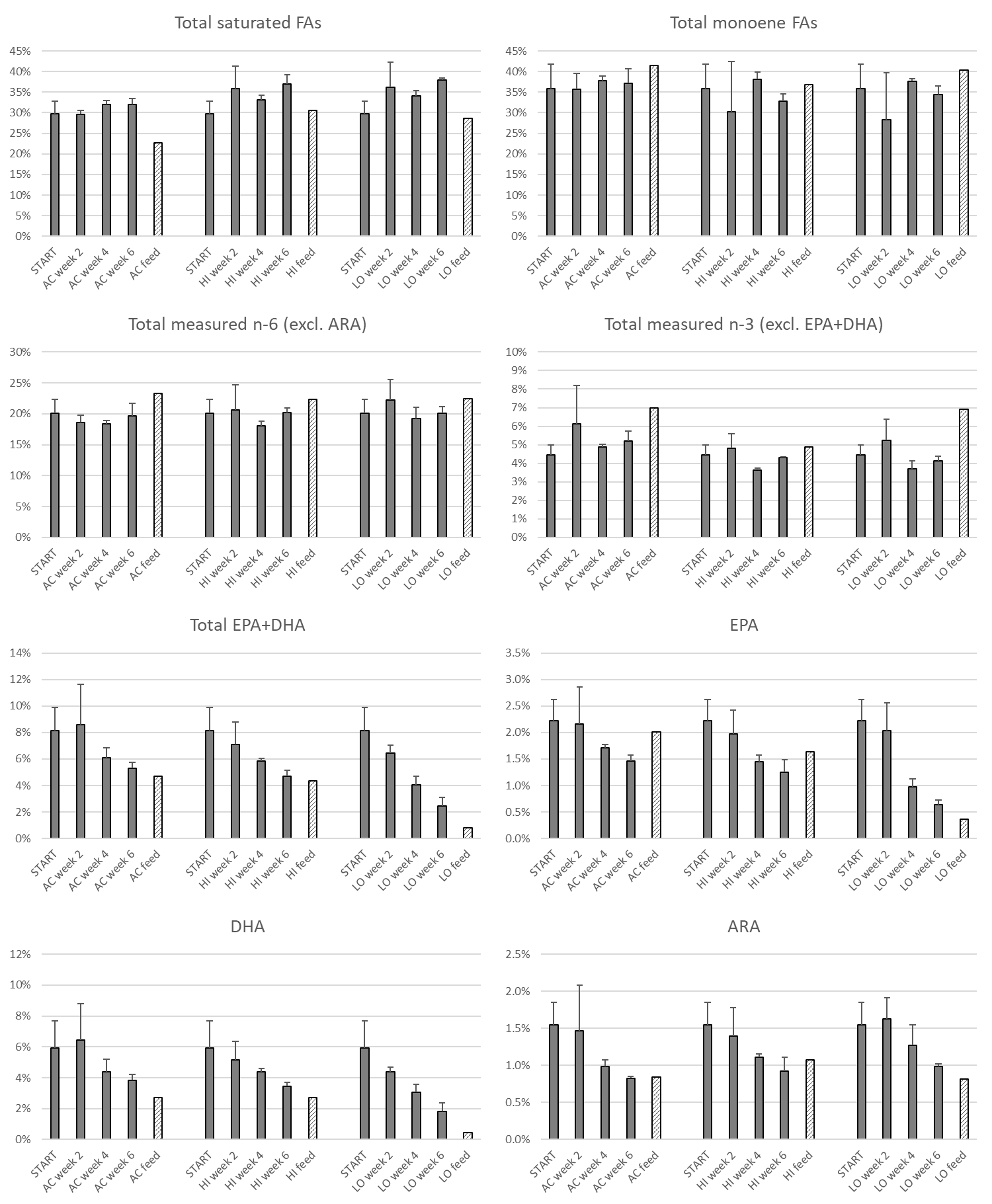


**Figure S8.** Relative proportion of fatty acid (FA) groups and certain FAs as a percentage (%) of total measured FAs in the filet of the fish. Error bars represent standard deviations; *n* = 3.

**Table S1.** Filet fatty acid content for week 2, 4 and 6 of the trial (g/kg DM).

|  |  | Week 2 | | | Week 4 | | | Week 6 | | |
| --- | --- | --- | --- | --- | --- | --- | --- | --- | --- | --- |
|  | Start | AC | HI | LO | AC | HI | LO | AC | HI | LO |
| C12:0 | 0.35 ± 0.05 | 0.55 ± 0.23 | 0.70 ± 0.04 | 0.90 ± 0.13 | 0.56 ± 0.07 ^b^ | 1.45 ± 0.13 ^a^ | 1.32 ± 0.17 ^a^ | 0.65 ± 0.05 ^b^ | 1.63 ± 0.21 ^a^ | 1.71 ± 0.25 ^a^ |
| C13:0 | 0.10 ± 0.01 | 0.11 ± 0.00 | 0.11 ± 0.01 | 0.10 ± 0.00 | 0.13 ± 0.02 | 0.15 ± 0.01 | 0.14 ± 0.02 | 0.12 ± 0.01 | 0.13 ± 0.01 | 0.14 ± 0.01 |
| C14:0 | 1.98 ± 0.39 | 2.35 ± 0.50 | 2.63 ± 0.20 | 2.70 ± 0.26 | 2.71 ± 0.43 ^ab^ | 3.47 ± 0.22 ^a^ | 2.54 ± 0.25 ^b^ | 3.26 ± 0.25 | 3.57 ± 0.63 | 2.68 ± 0.35 |
| C14:1n5 | 0.14 ± 0.02 | 0.17 ± 0.02 | 0.33 ± 0.16 | 0.17 ± 0.01 | 0.21 ± 0.03 | 0.25 ± 0.02 | 0.23 ± 0.03 | 0.21 ± 0.00 | 0.24 ± 0.03 | 0.25 ± 0.03 |
| C15:0 | 0.33 ± 0.05 | 0.36 ± 0.06 | 0.39 ± 0.01 | 0.40 ± 0.03 | 0.41 ± 0.06 | 0.50 ± 0.02 | 0.42 ± 0.05 | 0.46 ± 0.02 | 0.54 ± 0.07 | 0.45 ± 0.05 |
| C16:0 | 18.75 ± 3.42 | 24.14 ± 4.62 | 31.52 ± 4.18 | 25.93 ± 1.20 | 37.97 ± 4.90 | 39.60 ± 3.15 | 42.45 ± 8.06 | 49.06 ± 4.97 ^b^ | 60.26 ± 1.85 ^ab^ | 69.14 ± 10.25 ^a^ |
| C16:1n-9c | 4.57 ± 0.99 | 6.11 ± 1.12 | 7.88 ± 1.06 | 6.69 ± 0.50 | 9.71 ± 1.39 | 10.82 ± 1.36 | 9.95 ± 2.96 | 12.40 ± 1.78 | 16.20 ± 1.28 | 15.97 ± 3.10 |
| C17:0 | 0.37 ± 0.05 | 0.42 ± 0.08 | 0.45 ± 0.01 | 0.48 ± 0.02 | 0.49 ± 0.08 | 0.57 ± 0.03 | 0.50 ± 0.04 | 0.51 ± 0.04 | 0.58 ± 0.10 | 0.54 ± 0.06 |
| C17:1n7 | 0.13 ± 0.01 | 0.18 ± 0.04 ^b^ | 0.17 ± 0.04 ^b^ | 0.30 ± 0.03 ^a^ | 0.32 ± 0.04 | 0.38 ± 0.02 | 0.32 ± 0.08 | 0.33 ± 0.07 | 0.40 ± 0.08 | 0.36 ± 0.06 |
| C18:0 | 5.66 ± 0.99 | 7.37 ± 1.00 | 9.00 ± 1.39 | 7.91 ± 0.20 | 11.05 ± 1.82 | 11.94 ± 1.28 | 12.57 ± 2.84 | 15.15 ± 1.10 ^b^ | 19.40 ± 1.25 ^ab^ | 20.70 ± 2.75 ^a^ |
| C18:1n-9t | 0.22 ± 0.03 | 0.23 ± 0.03 | 0.30 ± 0.04 | 0.31 ± 0.08 | 0.33 ± 0.04 | 0.43 ± 0.09 | 0.39 ± 0.05 | 0.34 ± 0.07 | 0.42 ± 0.08 | 0.44 ± 0.05 |
| C18:1n-9c | 27.52 ± 9.39 | 34.17 ± 8.66 | 30.28 ± 22.15 | 23.78 ± 20.41 | 49.09 ± 5.86 | 51.97 ± 8.01 | 53.23 ± 9.74 | 64.04 ± 6.12 | 56.48 ± 7.92 | 67.72 ± 14.59 |
| C18:2n-6t | 0.55 ± 0.09 | 0.61 ± 0.01 ^a^ | 0.58 ± 0.06 ^ab^ | 0.51 ± 0.03 ^b^ | 0.67 ± 0.08 | 0.84 ± 0.06 | 0.59 ± 0.30 | 0.54 ± 0.11 | 0.68 ± 0.12 | 0.79 ± 0.07 |
| C18:2n-6c | 16.01 ± 3.36 | 18.80 ± 4.42 | 21.84 ± 2.30 | 19.57 ± 1.37 | 27.25 ± 4.23 | 26.79 ± 1.60 | 28.43 ± 7.29 | 39.07 ± 6.17 | 41.78 ± 2.51 | 43.70 ± 3.84 |
| C20:0 | 0.60 ± 0.09 | 0.74 ± 0.05 | 0.77 ± 0.06 | 0.83 ± 0.06 | 0.95 ± 0.13 | 0.90 ± 0.01 | 0.88 ± 0.15 | 0.97 ± 0.11 | 0.88 ± 0.16 | 0.95 ± 0.08 |
| C18:3n-6 | 1.08 ± 0.21 | 1.23 ± 0.24 ^b^ | 1.42 ± 0.05 ^b^ | 1.91 ± 0.07 ^a^ | 1.22 ± 0.18 ^c^ | 1.64 ± 0.08 ^b^ | 2.47 ± 0.12 ^a^ | 1.22 ± 0.07 ^b^ | 1.81 ± 0.66 ^ab^ | 2.97 ± 0.51 ^a^ |
| C20:1n-9 | 2.43 ± 0.50 | 3.28 ± 0.49 | 3.47 ± 0.36 | 3.43 ± 0.38 | 4.11 ± 0.22 ^ab^ | 4.27 ± 0.15 ^a^ | 3.30 ± 0.53 ^b^ | 4.85 ± 0.47 ^a^ | 4.31 ± 0.74 ^ab^ | 3.30 ± 0.33 ^b^ |
| C18:3n-3 | 4.02 ± 0.91 | 6.48 ± 0.71 | 5.85 ± 0.73 | 5.40 ± 0.21 | 7.93 ± 1.11 | 5.99 ± 0.40 | 6.36 ± 1.83 | 11.06 ± 1.64 | 9.65 ± 0.59 | 10.09 ± 0.92 |
| C20:2n-6 | 0.67 ± 0.13 | 0.79 ± 0.07 | 0.94 ± 0.07 | 0.84 ± 0.08 | 0.84 ± 0.03 ^b^ | 1.27 ± 0.05 ^a^ | 0.90 ± 0.15 ^b^ | 1.07 ± 0.15 | 1.29 ± 0.23 | 0.91 ± 0.10 |
| C22:0 | 0.28 ± 0.04 | 0.32 ± 0.04 | 0.32 ± 0.02 | 0.35 ± 0.02 | 0.41 ± 0.05 | 0.41 ± 0.02 | 0.43 ± 0.05 | 0.46 ± 0.04 | 0.45 ± 0.05 | 0.48 ± 0.05 |
| C20:3n-6 | 1.23 ± 0.18 | 1.51 ± 0.35 | 1.59 ± 0.10 | 1.78 ± 0.16 | 1.50 ± 0.22 ^b^ | 1.90 ± 0.14 ^ab^ | 2.35 ± 0.24 ^a^ | 1.65 ± 0.19 ^b^ | 2.28 ± 0.52 ^ab^ | 2.99 ± 0.52 ^a^ |
| C22:1n-9 | 0.05 ± 0.01 | 0.06 ± 0.01 | 0.06 ± 0.00 | 0.05 ± 0.00 | 0.07 ± 0.01 | 0.08 ± 0.01 | 0.06 ± 0.01 | 0.07 ± 0.01 | 0.07 ± 0.01 | 0.08 ± 0.01 |
| C20:3n-3 | 0.26 ± 0.03 | 0.30 ± 0.03 | 0.33 ± 0.03 | 0.29 ± 0.01 | 0.34 ± 0.01 ^b^ | 0.48 ± 0.03 ^a^ | 0.33 ± 0.06 ^b^ | 0.42 ± 0.08 ^ab^ | 0.56 ± 0.07 ^a^ | 0.35 ± 0.02 ^b^ |
| C20:4n-6 | 1.45 ± 0.16 | 1.56 ± 0.24 | 1.74 ± 0.02 | 1.79 ± 0.12 | 1.66 ± 0.23 ^b^ | 1.96 ± 0.11 ^ab^ | 2.21 ± 0.06 ^a^ | 1.81 ± 0.10 | 2.15 ± 0.50 | 2.51 ± 0.35 |
| C22:2n-6 | 0.29 ± 0.04 | 0.35 ± 0.04 | 0.39 ± 0.02 | 0.36 ± 0.03 | 0.43 ± 0.03 ^ab^ | 0.52 ± 0.04 ^a^ | 0.38 ± 0.05 ^b^ | 0.51 ± 0.04 ^ab^ | 0.59 ± 0.08 ^a^ | 0.41 ± 0.05 ^b^ |
| C20:5n-3 | 2.09 ± 0.33 | 2.37 ± 0.19 | 2.48 ± 0.13 | 2.21 ± 0.25 | 2.85 ± 0.36 ^a^ | 2.57 ± 0.07 ^a^ | 1.71 ± 0.07 ^b^ | 3.24 ± 0.39 ^a^ | 2.89 ± 0.69 ^a^ | 1.60 ± 0.14 ^b^ |
| C22:6n-3 | 5.55 ± 1.46 | 6.98 ± 0.90 ^a^ | 6.55 ± 0.36 ^ab^ | 4.85 ± 0.74 ^b^ | 7.43 ± 0.46 ^a^ | 7.80 ± 0.82 ^a^ | 5.34 ± 0.13 ^b^ | 8.42 ± 1.27 ^a^ | 8.14 ± 1.22 ^a^ | 4.53 ± 1.00 ^b^ |
| ∑ n-6 | 20.72 ± 3.79 | 24.24 ± 3.81 | 27.92 ± 2.32 | 26.24 ± 1.51 | 32.89 ± 4.78 | 34.08 ± 1.82 | 36.73 ± 7.61 | 45.31 ± 6.69 | 49.91 ± 3.89 | 53.50 ± 5.05 |
| ∑ n-3 | 11.93 ± 1.94 | 16.13 ± 1.81 | 15.21 ± 1.23 | 12.75 ± 0.58 | 18.56 ± 1.12 ^a^ | 16.84 ± 1.22 ^ab^ | 13.74 ± 2.04 ^b^ | 23.13 ± 3.36 | 21.24 ± 2.55 | 16.56 ± 1.39 |
| ∑ n-6:∑n-3 | 1.74 ± 0.25 | 1.55 ± 0.33 ^ab^ | 1.86 ± 0.05 ^b^ | 2.06 ± 0.06 ^a^ | 1.77 ± 0.17 ^b^ | 2.03 ± 0.04 ^b^ | 2.67 ± 0.27 ^a^ | 1.96 ± 0.02 ^b^ | 2.36 ± 0.14 ^ab^ | 3.24 ± 0.32 ^a^ |
| ∑ saturated FAs | 28.42 ± 4.86 | 36.35 ± 4.78 | 45.89 ± 5.71 | 39.59 ± 1.90 | 54.69 ± 7.38 | 59.01 ± 4.77 | 61.19 ± 11.55 | 70.64 ± 6.32 | 87.42 ± 1.56 | 96.79 ± 13.58 |
| ∑ monoene FAs | 35.06 ± 10.17 | 44.21 ± 9.27 | 42.49 ± 23.77 | 34.74 ± 21.30 | 63.84 ± 7.06 | 68.19 ± 9.53 | 67.45 ± 11.61 | 82.24 ± 4.47 | 78.13 ± 10.02 | 88.13 ± 17.41 |
| EPA+DHA | 7.64 ± 1.38 | 9.34 ± 1.07 ^a^ | 9.03 ± 0.48 ^ab^ | 7.06 ± 0.63 ^b^ | 10.28 ± 0.18 ^a^ | 10.38 ± 0.82 ^a^ | 7.05 ± 0.17 ^b^ | 11.66 ± 1.66 ^a^ | 11.02 ± 1.90 ^a^ | 6.13 ± 1.11 ^b^ |
| EPA:DHA | 0.40 ± 0.13 | 0.34 ± 0.03 | 0.39 ± 0.02 | 0.49 ± 0.10 | 0.41 ± 0.11 | 0.33 ± 0.04 | 0.32 ± 0.01 | 0.39 ± 0.01 | 0.36 ± 0.05 | 0.37 ± 0.09 |
| ∑ total FAs | 96.13 ± 18.76 | 120.93 ± 16.58 | 131.52 ± 32.72 | 113.32 ± 25.01 | 169.98 ±19.86 | 178.12 ± 16.92 | 179.11 ± 31.74 | 221.32 ± 10.85 | 236.70 ± 17.54 | 254.97 ± 36.64 |
| Values represent means ± standard deviations; means in rows for the respective week with different superscript letters are significantly different (p < 0.05); *n* = 3. | | | | | | | | | | |

**Table S2.** Liver fatty acid content for week 2, 4 and 6 of the trial (g/kg DM).

|  | Week 2 | | | | Week 4 | | | Week 6 | | |
| --- | --- | --- | --- | --- | --- | --- | --- | --- | --- | --- |
|  | Start | AC | HI | LO | AC | HI | LO | AC | HI | LO |
| C12:0 | - | - | - | - | - | - | - | - | - | - |
| C13:0 | - | - | - | - | - | - | - | - | - | - |
| C14:0 | 3.13 ± 0.72 | 5.36 ± 0.95 ^a^ | 3.94 ± 0.84 ^b^ | 3.78 ± 0.22 ^b^ | 4.20 ± 0.75 ^ab^ | 4.91 ± 0.97 ^a^ | 3.43 ± 1.05 ^b^ | 3.34 ± 0.47 ^ab^ | 4.40 ± 0.42 ^a^ | 2.62 ± 1.00 ^b^ |
| C14:1n5 | - | - | - | - | - | - | - | - | - | - |
| C15:0 | 0.66 ± 0.08 | 0.86 ± 0.17 ^a^ | 0.61 ± 0.19 ^ab^ | 0.53 ± 0.03 ^b^ | 0.66 ± 0.03 ^a^ | 0.75 ± 0.13 ^a^ | 0.47 ± 0.14 ^b^ | 0.59 ± 0.08 ^ab^ | 0.81 ± 0.07 ^a^ | 0.43 ± 0.15 ^b^ |
| C16:0 | 27.46 ± 6.80 | 53.77 ± 7.82 | 53.52 ± 4.59 | 54.06 ± 3.58 | 55.73 ± 21.33 | 54.46 ± 5.02 | 42.93 ± 10.63 | 45.59 ± 6.01 | 64.77 ± 14.70 | 43.75 ± 8.72 |
| C16:1n-9c | 6.18 ± 1.73 | 12.36 ± 1.89 | 12.58 ± 1.40 | 12.60 ± 1.32 | 10.79 ± 2.81 | 14.16 ± 1.43 | 10.65 ± 1.99 | 10.18 ± 2.01 | 14.65 ± 3.20 | 10.87 ± 2.80 |
| C17:0 | 1.16 ± 0.07 | 1.56 ± 0.21 ^a^ | 1.18 ± 0.18 ^b^ | 1.19 ± 0.05 ^b^ | 1.29 ± 0.13 ^ab^ | 1.33 ± 0.27 ^a^ | 0.97 ± 0.26 ^b^ | 1.11 ± 0.11 ^ab^ | 1.34 ± 0.05 ^a^ | 0.91 ± 0.24 ^b^ |
| C17:1n7 | 0.43 ± 0.13 | 1.10 ± 0.49 | 1.46 ± 0.67 | 1.06 ± 0.06 | 1.14 ± 0.13 ^ab^ | 1.37 ± 0.27 ^a^ | 0.95 ± 0.21 ^b^ | 1.01 ± 0.10 ^ab^ | 1.27 ± 0.11 ^a^ | 0.77 ± 0.20 ^b^ |
| C18:0 | 14.66 ± 3.04 | 29.56 ± 4.61 | 27.74 ± 2.25 | 31.21 ± 3.18 | 30.11 ± 14.25 | 28.69 ± 4.65 | 22.24 ± 5.92 | 24.41 ± 2.31 | 31.52 ± 5.39 | 24.49 ± 6.07 |
| C18:1n-9t | 1.13 ± 0.14 | 2.23 ± 0.14 ^a^ | 1.83 ± 0.24 ^b^ | 1.89 ± 0.04 ^b^ | 1.62 ± 0.33 | 1.75 ± 0.66 | 1.42 ± 0.23 | 1.43 ± 0.12 | 1.66 ± 0.24 | 1.30 ± 0.17 |
| C18:1n-9c | 28.66 ± 5.72 | 54.26 ± 9.63 | 51.92 ± 5.40 | 61.95 ± 4.04 | 48.78 ± 14.36 | 54.34 ± 9.70 | 43.01 ± 11.27 | 44.41 ± 5.96 ^ab^ | 57.56 ± 3.79 ^a^ | 38.76 ± 8.00 ^b^ |
| C18:2n-6t | - | - | - | - | - | - | - | - | - | - |
| C18:2n-6c | 10.21 ± 1.21 | 12.77 ± 2.51 | 12.32 ± 2.28 | 12.66 ± 0.95 | 12.17 ± 0.79 | 12.36 ± 1.02 | 9.74 ± 1.97 | 12.97 ± 0.91 | 15.97 ± 2.02 | 13.00 ± 3.08 |
| C20:0 | 1.78 ± 0.19 | 2.99 ± 0.42 ^a^ | 2.49 ± 0.16 ^b^ | 2.50 ± 0.18 ^b^ | 2.56 ± 0.56 ^a^ | 2.61 ± 0.45 ^a^ | 1.87 ± 0.25 ^b^ | 2.22 ± 0.16 ^a^ | 2.27 ± 0.20 ^a^ | 1.71 ± 0.19 ^b^ |
| C18:3n-6 | 1.56 ± 0.21 | 2.33 ± 0.20 | 2.09 ± 0.17 | 2.50 ± 0.09 | 1.58 ± 0.13 ^b^ | 1.97 ± 0.25 ^a^ | 1.91 ± 0.17 ^ab^ | 1.51 ± 0.10 ^b^ | 2.08 ± 0.19 ^a^ | 1.94 ± 0.51 ^ab^ |
| C20:1n-9 | 5.93 ± 0.82 | 9.69 ± 1.99 | 8.74 ± 0.92 | 8.59 ± 0.60 | 8.76 ± 1.24 | 8.78 ± 1.36 | 6.43 ± 1.97 | 9.34 ± 0.82 | 11.16 ± 1.97 | 8.08 ± 1.34 |
| C18:3n-3 | 2.69 ± 0.22 | 3.00 ± 0.48 ^a^ | 2.15 ± 0.31 ^b^ | 2.12 ± 0.20 ^b^ | 2.76 ± 0.24 ^a^ | 2.34 ± 0.31 ^ab^ | 1.64 ± 0.24 ^b^ | 2.52 ± 0.23 ^a^ | 2.41 ± 0.08 ^ab^ | 1.76 ± 0.39 ^b^ |
| C20:2n-6 | 2.49 ± 0.21 | 2.92 ± 0.49 | 2.49 ± 0.23 | 2.24 ± 0.18 | 2.54 ± 0.12 ^a^ | 2.59 ± 0.41 ^a^ | 1.80 ± 0.36 | 2.54 ± 0.19 ^a^ | 2.91 ± 0.06 ^a^ | 2.07 ± 0.21 ^b^ |
| C22:0 | 0.97 ± 0.42 | 1.60 ± 0.35 | 1.82 ± 0.04 | 2.01 ± 0.19 | 1.36 ± 0.05 | 1.04 ± 0.27 | 1.33 ± 0.58 | 1.02 ± 0.29 | 1.14 ± 0.37 | 0.61 ± 0.61 |
| C20:3n-6 | 6.53 ± 0.64 | 7.77 ± 0.89 | 8.29 ± 0.83 | 8.63 ± 0.54 | 6.59 ± 0.27 | 6.79 ± 0.20 | 7.61 ± 0.74 | 8.12 ± 0.89 ^b^ | 10.46 ± 1.32 ^ab^ | 12.27 ± 0.12 ^a^ |
| C22:1n-9 | 0.90 ± 0.26 | 1.42 ± 0.19 | 1.05 ± 0.29 | 1.10 ± 0.09 | 1.14 ± 0.17 ^a^ | 1.12 ± 0.12 ^a^ | 0.72 ± 0.21 ^b^ | 1.03 ± 0.24 | 1.08 ± 0.13 | 0.77 ± 0.24 |
| C20:3n-3 | 1.37 ± 0.14 | 1.75 ± 0.32 | 1.26 ± 0.33 | 1.11 ± 0.25 | 1.40 ± 0.09 ^a^ | 1.25 ± 0.16 ^ab^ | 0.50 ± 0.45 ^b^ | 1.35 ± 0.13 | 1.51 ± 0.13 | 1.21 ± 0.06 |
| C20:4n-6 | 3.73 ± 0.29 | 3.96 ± 0.26 | 3.70 ± 0.41 | 3.99 ± 0.26 | 2.86 ± 0.21 ^b^ | 2.88 ± 0.28 ^b^ | 3.66 ± 0.32 ^a^ | 2.99 ± 0.09 ^b^ | 3.46 ± 0.18 ^b^ | 3.95 ± 0.24 ^a^ |
| C22:2n-6 * | 1.36 ± 0.11 | 1.68 ± 0.28 | 1.35 ± 0.33 | 1.06 ± 0.24 | 0.97 ± 0.31 | 1.20 ± 0.10 | 1.33 ± 0.26 | - | - | - |
| C20:5n-3 | 2.38 ± 0.17 | 2.65 ± 0.14 ^a^ | 2.23 ± 0.14 ^b^ | 1.84 ± 0.07 ^c^ | 2.11 ± 0.15 ^a^ | 1.89 ± 0.04 ^a^ | 1.46 ± 0.15 ^b^ | 2.21 ± 0.16 ^a^ | 2.11 ± 0.12 ^a^ | 1.76 ± 0.02 ^b^ |
| C22:6n-3 | 11.00 ± 1.12 | 10.91 ± 1.25 | 10.17 ± 2.85 | 9.54 ± 1.02 | 9.01 ± 0.22 ^a^ | 7.88 ± 0.32 ^b^ | 6.49 ± 0.59 ^c^ | 11.30 ± 1.38 | 14.13 ± 3.00 | 13.37 ± 0.82 |
| ∑ n-6 | 25.88 ± 2.12 | 31.43 ± 4.39 | 30.24 ± 2.68 | 31.08 ± 1.29 | 26.70 ± 1.40 | 27.79 ± 2.08 | 26.05 ± 2.74 | 28.12 ± 1.72 | 34.89 ± 3.39 | 33.23 ± 3.71 |
| ∑ n-3 | 17.44 ± 1.28 | 18.31 ± 2.07 | 15.81 ± 2.30 | 14.61 ± 0.91 | 15.28 ± 0.68 ^a^ | 13.36 ± 0.57 ^b^ | 9.85 ± 0.78 ^c^ | 17.38 ± 1.44 | 20.15 ± 3.29 | 18.10 ± 0.71 |
| ∑ n-6:∑n-3 | 1.49 ± 0.10 | 1.74 ± 0.11 ^b^ | 2.00 ± 0.51 ^ab^ | 2.13 ± 0.05 ^a^ | 1.75 ± 0.05 ^b^ | 2.08 ± 0.09 ^a^ | 2.78 ± 0.57 ^ab^ | 1.62 ± 0.04 | 1.75 ± 0.12 | 1.84 ± 0.23 |
| ∑ saturated FA | 49.83 ± 10.61 | 95.69 ± 13.97 | 91.30 ± 6.23 | 95.29 ± 6.10 | 95.91 ± 36.95 | 93.79 ± 11.37 | 73.24 ± 17.46 | 78.29 ± 9.19 | 106.26 ± 19.49 | 74.52 ± 16.95 |
| ∑ monoene FA | 43.16 ± 8.35 | 81.07 ± 13.78 | 77.46 ± 7.96 | 86.80 ± 5.83 | 72.23 ± 18.95 | 81.53 ± 13.40 | 63.18 ± 15.72 | 67.41 ± 9.06 | 87.38 ± 8.77 | 60.55 ± 12.66 |
| EPA+DHA | 13.38 ± 1.14 | 13.56 ± 1.29 | 12.40 ± 2.90 | 11.38 ± 1.09 | 11.12 ± 0.37 ^a^ | 9.77 ± 0.29 ^b^ | 7.71 ± 0.62 ^c^ | 13.51 ± 1.53 | 16.23 ± 3.12 | 15.13 ± 0.80 |
| EPA:DHA | 0.22 ± 0.03 | 0.27 ± 0.06 | 0.25 ± 0.08 | 0.19 ± 0.01 | 0.23 ± 0.01 | 0.24 ± 0.01 | 0.27 ± 0.07 | 0.20 ± 0.01 ^a^ | 0.15 ± 0.02 ^b^ | 0.13 ± 0.01 ^b^ |
| ∑ total FAs | 134.95 ± 20.56 | 224.83 ± 33.36 | 213.47 ± 15.68 | 226.73 ± 13.73 | 209.15 ± 57.20 | 215.27 ± 27.11 | 170.98 ± 35.97 | 191.20 ± 21.29 | 248.68 ± 33.11 | 186.41 ± 33.31 |
| Values represent means ± standard deviations; means in rows for the respective week with different superscript letters are significantly different (p < 0.05); *n* = 3. * Not included in ∑ total FAs, ∑ n-6, and ∑ n-6:∑n-3 due to C22:2n-6 being undetectable in week 6. | | | | | | | | | | |
